# Complementary Quantitative Structure–Activity Relationship Models for the Antitrypanosomal Activity of Sesquiterpene Lactones

**DOI:** 10.3390/ijms19123721

**Published:** 2018-11-22

**Authors:** Njogu M. Kimani, Josphat C. Matasyoh, Marcel Kaiser, Mauro S. Nogueira, Gustavo H. G. Trossini, Thomas J. Schmidt

**Affiliations:** 1 Institute of Pharmaceutical Biology and Phytochemistry (IPBP), University of Muenster, PharmaCampus Corrensstrasse 48, D-48149 Muenster, Germany; m_kima01@uni-muenster.de (N.M.K.); mauro.nogueira@tu-dortmund.de (M.S.N.); 2 Department of Chemistry, Egerton University, P.O. Box 536, Egerton 20115, Kenya; josphat2001@yahoo.com; 3 Swiss Tropical and Public Health Institute (Swiss TPH), Socinstrasse 57, CH-4051 Basel, Switzerland; marcel.kaiser@unibas.ch; 4 University of Basel, Petersplatz 1, CH-4003 Basel, Switzerland; 5 Faculdade de Ciências Farmacêuticas, Universidade de São Paulo, Av. Lineu Prestes 580, 05508-000 São Paulo, Brazil; trossini@usp.br

**Keywords:** QSAR, sesquiterpene lactones, *Trypanosoma brucei*, human African trypanosomiasis, pharmacophore-based drug design

## Abstract

Three complementary quantitative structure–activity relationship (QSAR) methodologies, namely, regression modeling based on (i) “classical” molecular descriptors, (ii) 3D pharmacophore features, and (iii) 2D molecular holograms (HQSAR) were employed on the antitrypanosomal activity of sesquiterpene lactones (STLs) toward *Trypanosoma brucei rhodesiense* (*Tbr*), the causative agent of the East African form of human African trypanosomiasis. In this study, an extension of a previous QSAR study on 69 STLs, models for a much larger and more diverse set of such natural products, now comprising 130 STLs of various structural subclasses, were established. The extended data set comprises a variety of STLs isolated and tested for antitrypanosomal activity within our group and is furthermore enhanced by 12 compounds obtained from literature, which have been tested in the same laboratory under identical conditions. Detailed QSAR analyses yielded models with comparable and good internal and external predictive ability. For a set of compounds as chemically diverse as the one under study, the models exhibited good coefficients of determination (R^2^) ranging from 0.71 to 0.85, as well as internal (leave-one-out Q^2^ values ranging from 0.62 to 0.72) and external validation coefficients (*P*^2^ values ranging from 0.54 to 0.73). The contributions of the various tested descriptors to the generated models are in good agreement with the results of previous QSAR studies and corroborate the fact that the antitrypanosomal activity of STLs is very much dependent on the presence and relative position of reactive enone groups within the molecular structure but is influenced by their hydrophilic/hydrophobic properties and molecular shape.

## 1. Introduction

Human African trypanosomiasis (HAT), commonly referred to as sleeping sickness, is a protozoan neglected tropical disease, which occurs in sub-Saharan Africa where the insect vectors, tsetse flies of the *Glossina* species, are endemic. There are two forms of HAT: a chronic form endemic in central and western Africa caused by *Trypanosoma brucei gambiense* and an acute form prevalent in southern and eastern Africa caused by *Trypanosoma brucei rhodesiense* (*Tbr*) [1]. HAT has claimed millions of people’s lives since early last century and it is still a major health concern in more than 20 countries in Africa with millions of people at risk of infection [1]. The currently available chemotherapeutic options for the treatment of HAT with only a few drugs are inadequate due to high toxicity, high cost, difficulty in administration and unavailability of drugs to resource-deprived rural communities. Therefore, new safe, effective, and affordable drugs are urgently needed [2].

**Figure 1:**
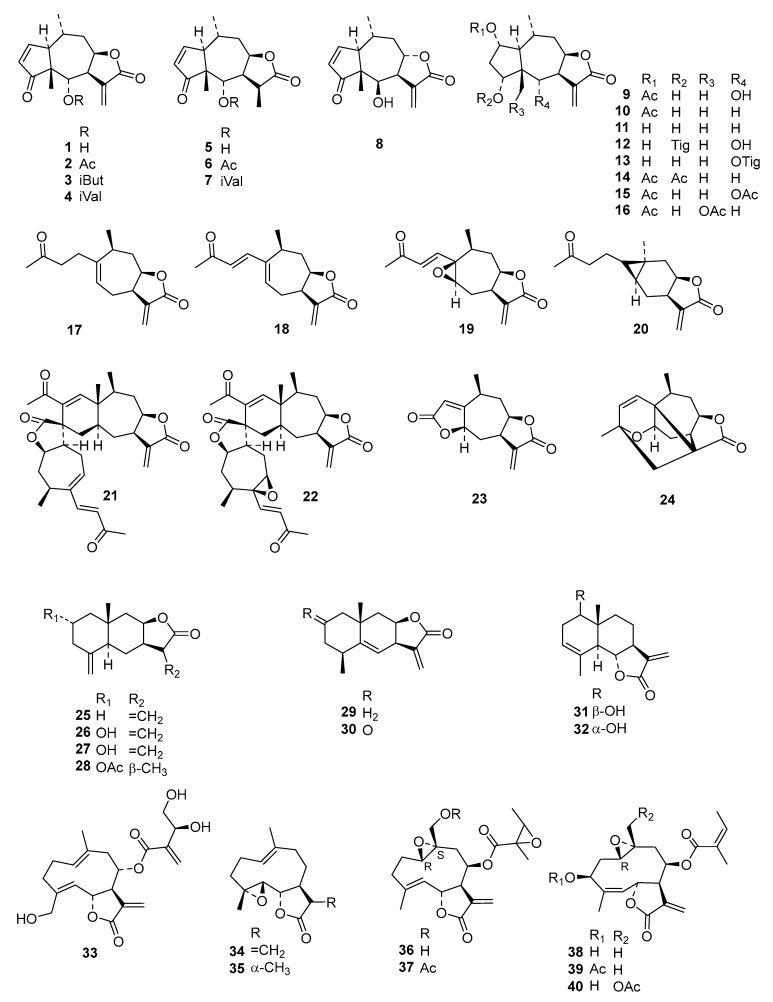

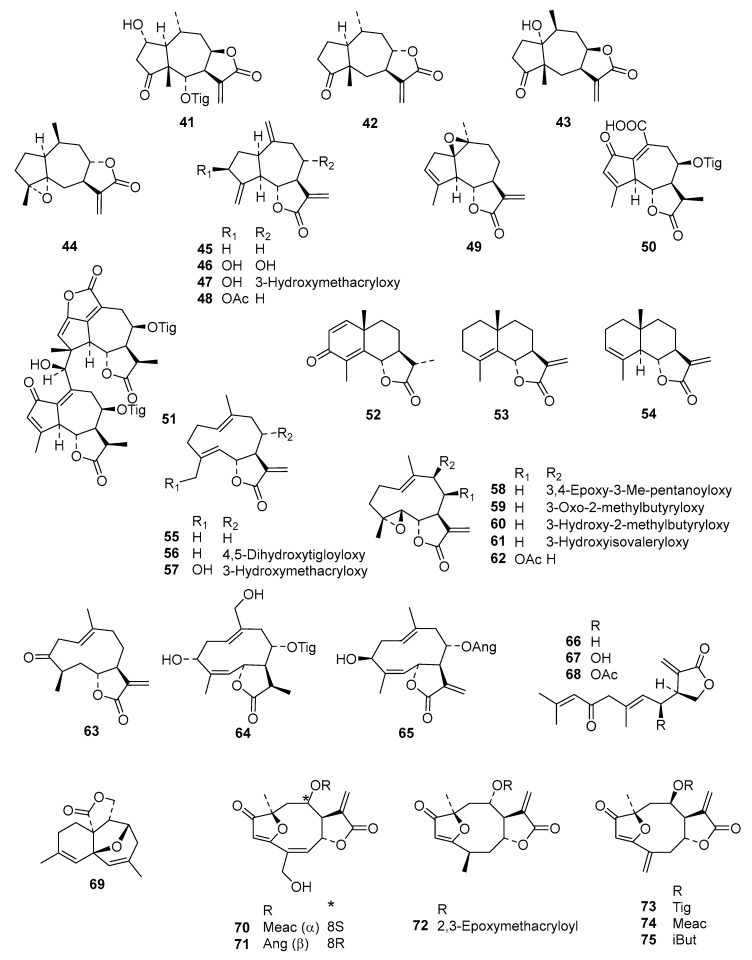

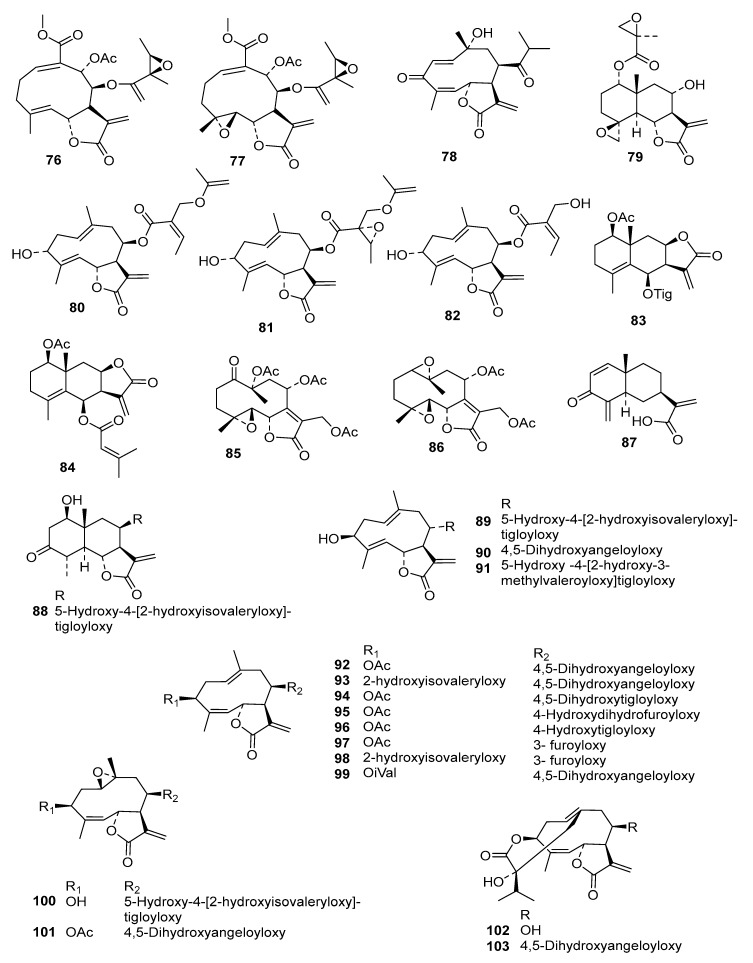

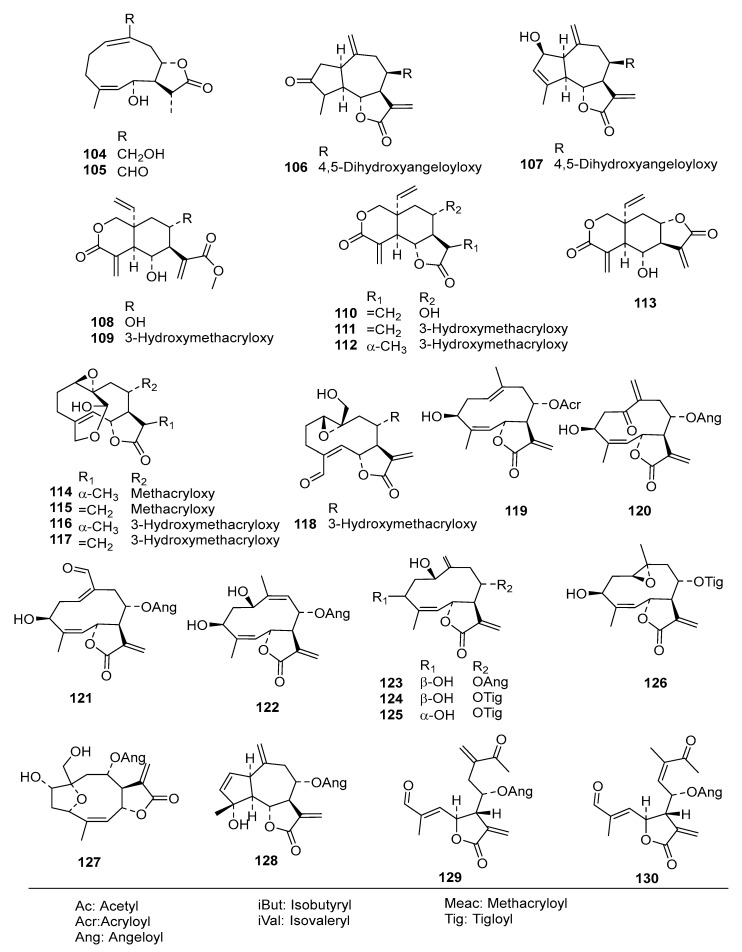
Structures of STLs used in the present QSAR analysis. Part 1 of the data set with compounds included in our first QSAR study [9]. **1**–**16**: pseudoguaianolides; **17**–**24**: xanthanolides and derivatives; **25**–**32**: eudesmanolides; **33**–**40**: germacranolides. Structures of STLs used in the present QSAR analysis. Part 2 of the data set with compounds included in our second QSAR study [9]. **41**–**43**: pseudoguaianolides; **44**–**51**: guaianolides and derivatives; **52**–**54**: eudesmanolides; **55**–**65**: germacranolides. **66**–**69** no subclass names; **70**–**75**: furanoheliangolides. Structures of STLs used in the present QSAR analysis. Part 3 of the data set with compounds newly included in this QSAR study. **76**–**78**, **80**–**82**, **85**–**86**, **89**–**103**: germacranolides; **79**, **83**–**84**, **87**–**88**: eudesmanolides. Structures of STLs used in the present QSAR analysis. Part 3 of the data set with compounds newly included in this QSAR study. **104**–**105**, **114**–**126**: germacranolides; **106**–**107**, **128**: guaianolides; **79**, **83**–**84**, **87**–**88**: eudesmanolides; **108**–**113**: elemanolides; **127**: furanoheliangolide; **129**–**130**: no subclass name.

Natural products have been shown in many instances to be an excellent source for drugs and drug leads [3]. Against African trypanosomal infections, interesting leads from natural products have frequently been reported [4]. Moreover, through a sustained study within our group, it has been shown that certain sesquiterpene lactones (STLs) are potent antitrypanosomal agents [5,6,7,8,9,10,11]. Quantitative structure–activity relationship (QSAR) studies of STLs and their antitrypanosomal activity have been carried out in our group [8,9,12]. With the last of these studies, based on 69 STLs, having been carried out in 2014 [9], to date we have expanded the number of STLs to 130 (see Figure 1). With the increased number of compounds, the chemical diversity of the data set has been greatly extended, now including almost twice as many compounds as before and some classes of STLs not represented in the previous analyses, such as elemanolides and melampolides. Three different QSAR approaches, all using linear regression modeling, were applied to this chemically diverse data set: (1) “Classical” descriptor-based QSAR using a genetic algorithm to select the most relevant variables, i.e., the same approach as in our previous study [9], (2) indicator variables deduced from pharmacophore features obtained from a 3D alignment of the most active molecules as applied in [13] and (3) hologram QSAR (HQSAR) based on molecular fingerprints of fragments extracted from the 2D molecular structure as used, e.g., in [12].

## 2. Results and Discussion

Even though the previously developed model [9] was used for external prediction of a set of about 1700 STLs, which led to the discovery of furanoheliangolides as strong trypanocides, it was found that activity predictions of the newly tested molecules with this model yielded only a relatively poor correlation between the predicted and experimental data (coefficient of determination (R^2^) = 0.20, see Figure S1). In order to obtain a more comprehensive QSAR model to be used for even more reliable external predictions, the in vitro anti-*Tbr* data recently obtained was combined with those of earlier studies (for a full list of pIC_50_ values, see Table S2) into an even more diverse chemical data set than in the former studies, consisting of 130 STLs (Figure 1) with pIC_50_ values ranging from 3.5 to 7.8, which was then used for extended QSAR studies using three different complementary approaches.

“*Classical*” *descriptor-based QSAR approach—Models 1 and 2*: A set of 123 molecular descriptors (see Table S1) were calculated for each of the 130 compounds based on its 3D-molecular structures. For Model 1, only the lowest energy conformer found in a conformational search was used for this purpose. STLs in many cases show a high degree of conformational flexibility. It has been shown in a previous quantitative structure–property relationship study aiming at prediction of HPLC retention times of such compounds, that models taking this into account by application of Boltzmann-weighted descriptors performed significantly better than such based on a single low-energy conformer [14]. In order to test whether this might also be the case with QSAR, the descriptors for Model 2 were generated for a conformational ensemble of each compound and their Boltzmann weighted averages calculated. The data set was then randomly divided into training (*n* = 87 and 90 for Models 1 and 2, respectively) and test (*n* = 43 and 40 for Models 1 and 2, respectively; see Table S3) sets. Subsequently, QSAR analysis was performed on the training sets by means of multiple linear regression (MLR) modeling using a genetic algorithm (GA) for the selection of the most useful descriptors (GA/MLR). The GA was operated at a fixed model length (= model size, i.e., the same number of descriptors used for each model of the population) and model sizes of five, six and seven descriptor variables were explored. Maximization of the coefficient of determination R^2^ for the correlation between experimental versus predicted data of the training set was used as optimization criterion for the GA. The GA/MLR procedure results in a predefined size of population or family of QSAR equations ranked by their R^2^ values. The optimized population of regression models (*n* = 100) was then validated in each case through a leave-one-out cross-validation (LOO-CV) resulting in a Q^2^ for each equation. The models were finally ranked by their Q^2^ values.

In Model 1, when the number of variables was increased from 5 to 6, the R^2^ and Q^2^ values of the best model equation registered a significant increase (R^2^_max_ and Q^2^_max_ rose from 0.66 to 0.72 and 0.61 to 0.67, respectively). A further increase of the number of descriptor variables to 7 only registered a marginal increase in the R^2^ and Q^2^ values from 0.72 to 0.73 and from 0.67 to 0.68, respectively. In Model 2, developed with a weighted Boltzmann descriptor averages, a similar trend was observed with the R^2^ and Q^2^ values increasing from 0.68 to 0.71 and from 0.64 to 0.67, respectively, when the number of descriptor variables was increased from 5 to 6. Additional increase of model size to 7 variables yielded no increase in Q^2^ values. Hence, six descriptor variables were found to be the optimum model size in both QSAR models. The 10 best 6-variable equations for Models 1 and 2 are listed in Table 1 and Table 2, respectively (see Tables S4 and S5 for a full list of 100 equations for each model). It becomes clear that Model 2 using Boltzmann-weighted average descriptors for each compound’s conformational ensemble did not perform better than Model 1 based on the lowest energy conformers.

**Figure 2:**
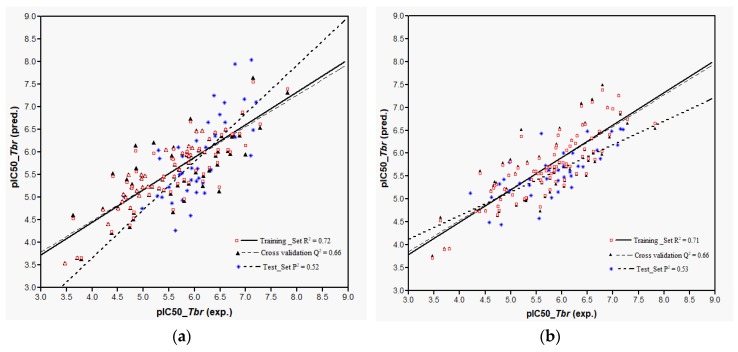
Plot of predicted versus experimental pIC_50_ values as obtained from the QSAR: (**a**) Model 1 represented by the first equation in Table 1; (**b**) Model 2 represented by first equation in Table 2. Note that three outliers were removed from the test set.

Plots of training and test sets of the best equations of Models 1 and 2, respectively, are shown in Figure 2a,b. Both models perform reasonably well in the prediction of the activity of most of the external test molecules. However, three compounds (**32**, **107**, and **119**, deviation of predicted from experimental pIC_50_ > 1.5 in each case) were not predicted very well and therefore considered outliers not well represented by the “classical” QSAR method used here. Thus, the coefficients of determination, *P*^2^, for the prediction using the best equations from Models 1 and 2 on all test set molecules were only 0.32 and 0.33, respectively. Compound **32**, which differs from **31** only in stereochemistry i.e., 1α-OH and 1β-OH, respectively, had already been found to be an outlier in the former model [9]. The model’s failure to predict the activity of this compound correctly can readily be attributed to the fact that its structural properties are not well represented in the training set. The poor prediction of Compounds **107** and **119** is not easily explained and the overestimation of their activity by the models may be due to several factors. Elimination of these outliers led to an increase of *P*^2^ to 0.52 in Model 1 and to 0.53 in Model 2. In any case, it becomes clear that Model 2 using Boltzmann-weighted average descriptors for each compound’s conformational ensemble did not perform better than Model 1 based on the lowest energy conformers.

Comparing the QSAR equations of the two models shows that two descriptors, ENONCS and ASAP4 appear in all equations. These descriptors were already found to be of high importance in the former QSAR model [9] so that their high relevance is confirmed on a much larger statistical basis. The descriptor ENONCS refers to the accessible surface area of reactive (electrophilic) carbon atoms in conjugated enone systems and represents here a measure of the potential of an STL to react with nucleophilic groups of a biological target. ASAP4 is the fractional accessible surface area [15] attributable to atoms with a partial charge between +0.15 and +0.2 e (electrons). This charge interval is populated by hydrogen atoms attached to the double-bond carbons in α,β-unsaturated ketone groups, so that this feature is also related to the presence of electrophilic Michael acceptor structures.

The descriptors FASA^−^ or FASA^+^ also contribute to all the equations, except Equation (10) in Model 1. These two descriptors are highly collinear (R^2^ = 0.99) and represent the ratio of accessible surface area contributed by atoms with negative and positive partial charges, respectively, over the total molecular surface. The descriptor FASA^−^, which was already found influential in the previous model [9], has a positive regression coefficient indicating an enhancing effect of negatively charged surface on activity. The negative partial charge, in this series of compounds, is mostly attributable to oxygen atoms, i.e., that this contribution is due to their tendency to accept H bonds. The descriptor FASA^+^ had a negative regression coefficient and just inversely reflects the same property as FASA^−^. This is consistent with the previous observation that descriptors of positive surface area had a negative effect [8].

Either the descriptor vsurf_CW1 or vsurf_CW2 was present in the 10 best equations in Model 1, while vsurf_CW2 appeared in the 10 best equations in Model 2. These descriptors, which show some collinearity (R^2^ = 0.51), correspond to properties of capacity factor, the ratio of the hydrophilic surface over the entire molecular surface, calculated at interaction energy levels with the water probe at −0.2 and −0.5 kcal/mol for vsurf_CW1 and vsurf_CW2, respectively [16]. Similar to the descriptor vsurf_CW1 and vsurf_CW2, the descriptor vsurf_ID1 or vsurf_ID2 appeared in the 10 best equations in Model 1 with only vsurf_ID2 occurring sparingly in Model 2. These descriptors, which are highly collinear (R^2^ = 0.98), correspond to the hydrophobic interaction energy (integy) moment calculated at −0.2 or −0.4 kcal/mol, respectively, from DRY probe 3D interaction maps. They are a measure of the unbalance between the center of mass of a molecule and the position (barycenter) of the hydrophobic region around it [16]. Thus, the presence and position of hydrophobic/hydrophilic groups around the molecule also appear to play a role in this bioactivity. It is interesting to note that the CW descriptors representing polar interactions with a water molecule display a negative regression coefficient throughout, whereas the ID descriptors, which reflect interactions with a hydrophobic probe, consistently have a positive impact on the overall regression.

**Figure 3:**
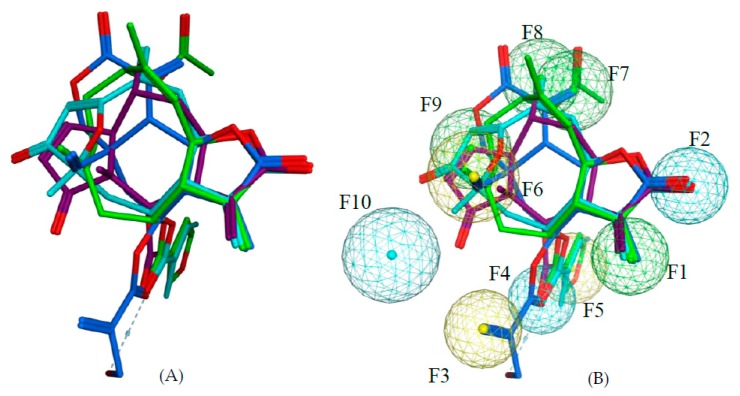
(**A**) Alignment as obtained by the flexible alignment function of MOE based on pharmacophoric atom properties; violet: **2**, light blue: **73**, green: **97**, and blue: **111**. (**B**) Common pharmacophore features of **2**, **73**, **97**, and **111**. Pharmacophore spheres: blue: hydrogen bond acceptor; yellow: hydrophobe; green: reactive carbon of α,β or α,β,γ,δ-unsaturated carbonyl groups. The features F1–F10 are numbered as used in the QSAR modeling.

**Figure 4:**
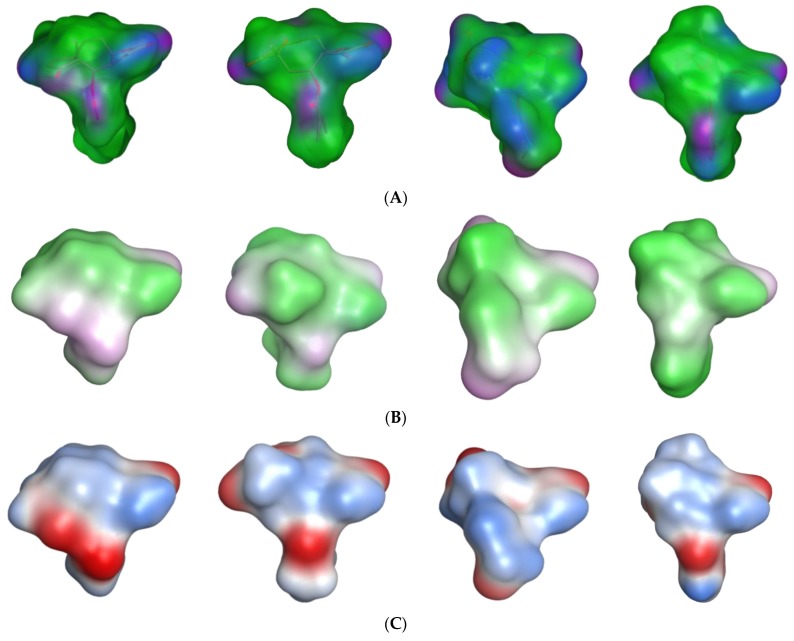
The molecular surfaces of **2**, **73**, **111**, and **97** (from left to right), the most active representative compounds of the different classes of STLs in the current series. Color schemes with regard to different properties are (**A**) active lone pair/hydrophobicity (pink: hydrogen bonding feature, green: hydrophobic, blue: polar), (**B**) lipophilicity (green: lipophilic, white: neutral, pink: hydrophilic), and (**C**) electrostatic potential (blue: positive, red: negative).

**Figure 5:**
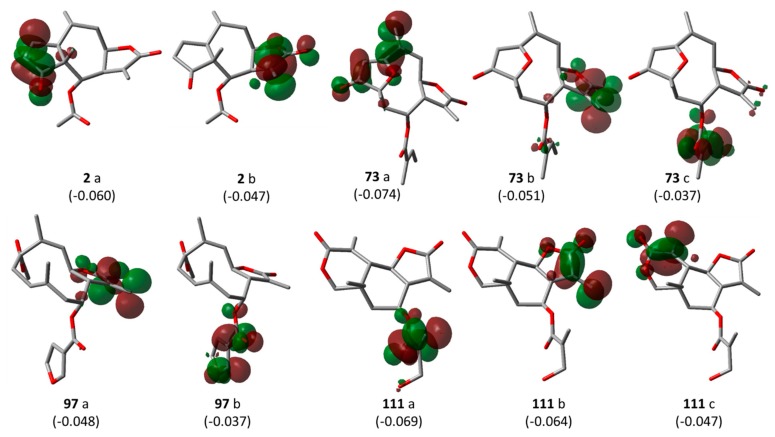
Localization of LUMO frontier molecular orbitals for **2**, **73**, **111**, and **97** (a = LUMO, b = LUMO + 1 and c = LUMO + 2 (isocontour value = 0.045 e); MO eigenvalues reported in brackets).

*Pharmacophore feature-based QSAR approach—Model 3*: In a previous study on the inhibition of tumor relevant transcription associated with transcription factor c-Myb by STLs, we applied a different type of QSAR approach which is based on the correlation of bioactivity with descriptors encoding pharmacophore features of the most active compounds in a series. We investigated whether this approach would also lead to useful QSAR models for the present data set, complementing the “classical” QSAR models described above. Compounds **2**, **73**, **97**, and **111** are all highly potent trypanocides with different molecular scaffolds. They were chosen to generate a common pharmacophore model, which was then mapped on the other compounds in the data set in an attempt to explain their diverse levels of activity with their degree of match with the presumable pharmacophore features. To this end, the four compounds were superposed using the pharmacophore-based flexible alignment feature in MOE yielding the alignment shown in Figure 3 and Figure 4. The comparison of their molecular surfaces reveals a rather high degree of resemblance in spite of the differences in their structural diagrams (Figure 4). Furthermore, a comparison of the localization of their lowest unoccupied molecular orbitals (LUMO, LUMO+1, and LUMO+2, Figure 5) reveals that these orbitals, the most susceptible points for nucleophilic attack, are localized around the reactive Michael acceptors in a very similar way in the four molecules. The MO eigenvalues of these orbitals are of similar magnitude, also indicating a comparable reactivity toward nucleophiles. The LUMO in Molecules **2** and **73** and LUMO+2 in **111** occupy largely the same region in the proposed pharmacophore alignment. Interestingly, in three of the four molecules (**2**, **73**, **111**), the LUMO+1 occupies essentially the same position. In **97**, this same region is occupied by LUMO. The four molecules hence would be able to interact via electrophilic/nucleophilic interactions with a hypothetical protein target in a very similar manner. It is thus obvious that these compounds share a similar shape and some properties that can be relevant for binding to a common hypothetical target (Figure 4 and Figure 5). Four of the features in the joint pharmacophore model (F1, F2, F4, and F6, representing the reactive β-methylene group of the α-methylene-γ-lactone ring and its corresponding carbonyl oxygen, the ester oxygen, and a hydrophobic feature for one of the methyl substituents, respectively) are identical in the four molecules (Figure 3). Only F9 and F3 were uniquely associated to Molecules **2** and **111**, respectively. The features representing the reactive carbons of the α,β,γ,δ-unsaturated ketone (F8) and of the α,β-unsaturated methylene pyranone structure (F7) features of **73** and **111**, respectively, occupy a similar position. The position of F9, representing the β-position of the cyclopentenone moiety of **2** on one hand, and F7 and F8 on the other is such that these electrophilic sites could react with the same nucleophilic structure element of a common hypothetical protein target. The pharmacophore feature F10, an H-bond acceptor, was identical in **2** and **73**. It corresponds to the carbonyl oxygens in the conjugated Michael acceptor systems encoded in F9 and F8 in **2** and **73**, respectively. Interaction of F10 with an H-bond donor of the receptor would enhance electrophilicity at F8/F9 and could thus increase the activity. Except in Molecule **111**, in which the ester moiety adopts a different orientation (feature F3), the hydrophobic ester sidechains in **2**, **73**, and **97** adopt the same position in the pharmacophoric alignment (feature F5). However, the positions of these moieties are in a similar region and could be envisaged to interact with a larger hydrophobic pocket of the hypothetical target.

Given this pharmacophore model, all other STLs in this study were aligned with the ensemble structural template of the four most active compounds using the flexible alignment feature as before. Each aligned structure was then visually inspected for the presence or absence of each of the 10 pharmacophoric features in the aligned structure and a value of 1 associated with the presence, 0 with the absence of each feature. Consequently, a matrix of 10 of such indicator variables was generated for the 130 STLs. The global alignment score (S), a measure for the quality of each molecule’s match with the template, was also included in the descriptor matrix. Partial least squares (PLS) regression was used to correlate the biological activity (pIC_50_) and the descriptors using the QSAR function implemented in MOE.

Prior to regression analysis, the data set was randomly divided into a training set (n = 86) and a test set (n = 44) as before ensuring that the range of biological data was distributed as evenly as possible in the two sets. In the PLS regression analysis performed with the training set and the 10 pharmacophore descriptors, the resulting *R*^2^ and Q^2^ values were 0.74 and 0.65, respectively. Addition of the global alignment score S to the descriptor matrix yielded a further slight increase of *R*^2^ value to 0.76 and Q^2^ to 0.66. Subsequently, variables of statistically insignificant contributions to the general regression were excluded by stepwise elimination optimizing the Q^2^ value (elimination of a variable should lead to an increase or at least no significant decrease of Q^2^). Thereby, the following multiple linear regression equation describing QSAR Model 3 was obtained:

**Table:** 

pIC_50_ (*Tbr*) = 1.8693 + 0.5940 F1 − 0.1728 F2 + 0.1552 F3 + 0.2672 F5 + 0.2714 F7 + 0.3563 F8 + 0.4939 F9 − 0.2190 S	(Model 3)

(*n* = 86; R^2^ = 0.73, RMSE = 0.42; Q^2^ = 0.66, RMSEP = 0.48; data were standardized to unit variance).

As already mentioned, the pharmacophore features encoding the reactive centers of the α,β-unsaturated γ-lactone (F7) in **111** and α,β,γ,δ-unsaturated ketone (F8) in **73** occupy a similar region in the biophore. They were therefore combined into a new feature, F7–8. Similarly, the hydrophobic features F3 in **111** and F5 in **73** and **110** were combined into a new feature F3–5. Replacement of the original features by these combinations did not lead to a dramatic decrease of statistical quality, which shows that the assumption of equivalence of these features is reasonable. This Model 4 with only five instead of eight variables is represented in the following QSAR equation:

**Table:** 

pIC_50_ (*Tbr*) = 1.46744 + 1.4336 F1 − 0.6970 F2 + 0.4715 F3−5 + 1.0902 F7−8 + 1.3065 F9 − 0.0233 S	(Model 4)

(*n* = 86; R^2^ = 0.71, RMSE = 0.44; Q^2^ = 0.66, RMSEP = 0.48; data were standardized to unit variance).

It is noteworthy that the statistical quality of this model based on the common pharmacophore was similarly high as that of the “classical” QSAR models. However, it is clear that some information encoded in the “classical” molecular descriptors may not be captured by this approach (and vice versa). It was therefore straightforward to make an attempt to combine the two approaches and thereby increase the overall quality of the model.

To this end, the pharmacophore descriptors were combined with the molecular descriptors used for Model 1 and subjected to a GA/MLR analysis as described above. The best QSAR equation (Model 5) obtained from this combined approach shows somewhat improved R^2^ and Q^2^ values in comparison with the models described so far. It consists of 6 variables as follows (see Table S6 for all the 100 equations obtained):

**Table:** 

pIC_50_ (*Tbr*) = 5.3407 + 1.1855 F1 + 0.6010 F3−5 + 0.8498 F7−8 + 1.3077 F9 − 6.3704 FASA^+^ + 3.6760 FASA_H	(Model 5)

(*n* = 86; R^2^ = 0.76, RMSE = 0.40; Q^2^ = 0.72, RMSEP = 0.44; data were standardized to unit variance).

**Figure 6:**
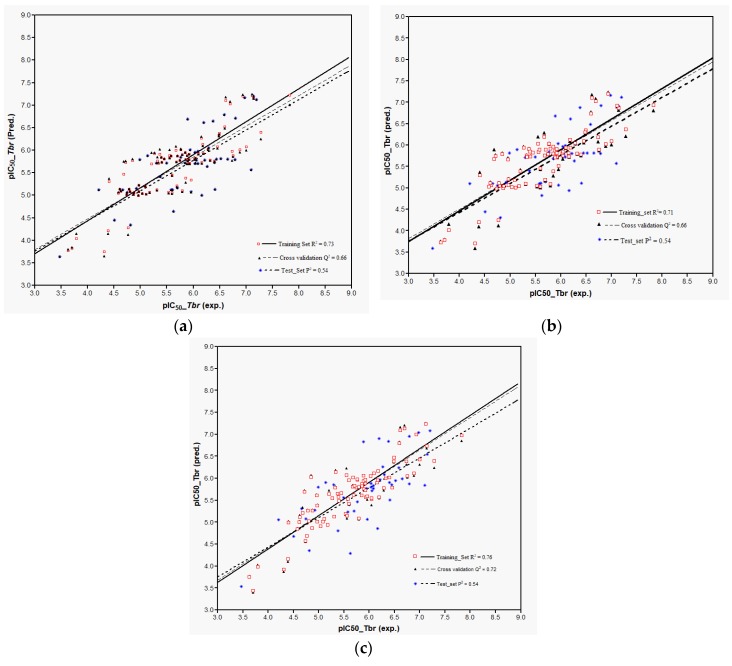
Plot of predicted versus experimental pIC_50_ values as obtained from the QSAR Models 3 (**a**), 4 (**b**), and 5 (**c**).

Models 3–5 were then used to predict the activity of the test set (*n* = 44) STLs, all resulting in a squared correlation coefficient (*P*^2^) of 0.54 for the predicted vs. the experimental data. This value may appear relatively low but it should be kept in mind that the predictions were made for a structurally very diverse set of compounds. The similar performance of all these models confirms their comparability and indicates that reasonable external predictions are possible. Plots of the experimental vs. predicted activity values are shown in Figure 6a–c for Models 3, 4, and 5, respectively.

Four of the pharmacophore descriptors found relevant account for three potentially reactive structure elements, namely, the reactive carbons of α-methylene-γ-lactone (F1), α-methylene-δ-lactone (F7), α,β,γ,δ-unsaturated ketone (F8), and cyclopentenone (F9) moieties. The presence of such structural features, each enhancing the bioactivity, has often been shown to be important for the biological effects of STLs, including antitrypanosomal activity [15,17,18,19]. Features F3 and F5 represent favorable hydrophobic interactions in the region occupied by the ester moieties of various compounds. Feature F2 encodes the hydrogen bond acceptor properties related with the carbonyl oxygen that would be expected to activate F1. It may appear paradoxical that this feature displays a negative regression coefficient in the QSAR equation. This can, however, be explained by the fact that some compounds have a saturated γ-lactone structure in this position so that the presence of the carbonyl oxygen *alone*, i.e., without the α,β-unsaturation, would indeed be associated with a negative effect on activity.

The statistically significant contributions of the mentioned pharmacophore descriptors to these QSAR models, explaining over 70% of the total variance in the activity data, confirm the relevance of the pharmacophore hypothesis based on the alignment of **2**, **73**, **97**, and **111**. This is further complemented by the significant contribution of the global alignment score S to Models 3 and 4, which accounts for the ability of each molecule to take on a conformation in which the pharmacophoric structure elements (as far as they are present) are oriented in the same way as in **2**, **73**, **97**, and **111**. The more negative the value of S, the better the fit, i.e., a higher pharmacophoric structural similarity and/or a lower strain energy related to the alignment. This descriptor consistently has a negative regression coefficient as expected in consideration of the general pharmacophore hypothesis. The descriptor FASA^+^, as in the other models demonstrates a negative influence on activity in Model 5 as in the “classical” QSAR models. FASA_H, which accounts for the fractional hydrophobic surface area, has a positive regression coefficient in Model 5, once more emphasizing the importance of lipophilicity.

*Hologram-QSAR (HQSAR) approach—Models 6–8*: HQSAR is a 2D-QSAR method based on molecular fingerprints of fragments extracted from the molecular graphs of the investigated compounds, and can hence be considered a fragment-based drug design approach [20,21,22,23]. It has been effectively used in a previous QSAR study of 40 antiprotozoal STLs where good models were obtained for antitrypanosomal, antileishmanial, and antiplasmodial activities as well as cytotoxicity [12]. To complement the QSAR methods described above, this method was therefore also applied to the present set of data. To this end, the training and test sets used in “classical” descriptor-based QSAR Model 1 were adopted. Firstly, the development of HQSAR models was performed with the default SYBYL fragment size default settings (4–7 atoms), various fragment distinction schemes, and all 12 standard options of hologram length. The three best PLS models obtained with these settings, resulting with three different fragment distinction schemes, are highlighted in bold characters in Table 3. The fragment distinction was then maintained and the fragment size was varied to determine the influence of this parameter on the statistical quality of the resulting models. For the three best fragment distinctions (Table 3), the fragment sizes: 2–5 atoms, 3–6 atoms, 5–8 atoms, and 6–9 atoms were evaluated, besides the 4–7 atoms scheme already mentioned.

In all cases, however, the fragment size setting of 4–7 atoms was found to represent an optimum yielding models with similar statistical characteristics. The model employing fragment distinction based on atoms, connections, hydrogen atoms, and chirality (A/C/H/Ch) provides the best description for the anti-*Tbr* activity with Q^2^ and R^2^ values of 0.63 and 0.85, respectively. However, the model based only on atoms, connections, and chirality (A/C/Ch) fragment distinction performs only slightly worse but is less complex since it employs one less PC in comparison with the others. It is also noteworthy that this model is easier to interpret and is hence described in more detail below.

**Figure 7:**
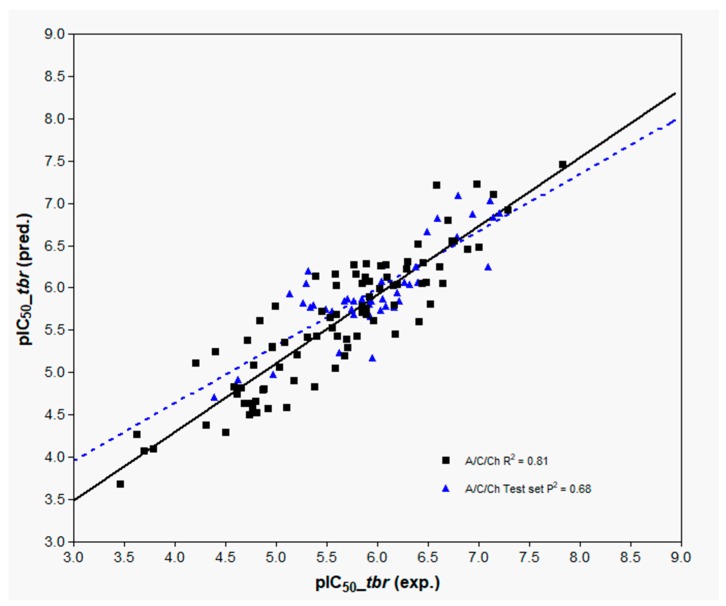
Experimental versus predicted pIC_50_ values of training and test sets of the A/C/Ch HQSAR model (Model 8).

The three best models, 6–8, subsequent to evaluation of the influence of fragment distinction, fragment size, hologram length, and the number of PCs, were then subjected to external validation by predicting the activity values for the external test set. The results are also reported in Table 3 and represented graphically in Figure 7 (A/C/Ch, Model 8) and Figures S2 and S3 (A/C/H/Ch and A/C Models 6 and 7, respectively).

**Figure 8:**
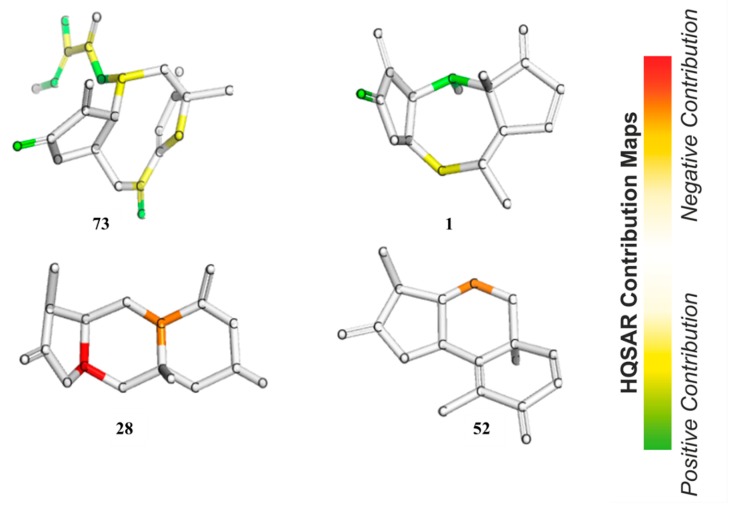
HQSAR maps of positive and negative contribution of the most active (**73**, **1**) and least active (**28**, **52**) compounds for the HQSAR model with A/C/Ch fragment distinction (Model 8).

In HQSAR, the PLS coefficients of individual fragments to an overall QSAR model can be mapped back into the molecular structures in order to investigate the positive or negative influences of structure elements on the bioactivity. The contribution maps of the two most and the two least active compounds obtained from Model 8 are shown in Figure 8. In Compound **73**, the most potent compound against *Tbr* in this series, the γ,δ-double bond containing the reactive δ-carbon of the α,β,γ,δ-unsaturated ketone structure (corresponding to pharmacophore feature F7–8 in Models 3–5) was assigned a strong positive contribution to anti-*Tbr* activity, which is in very good agreement with the models obtained by the other QSAR approaches. Interestingly, the carbonyl oxygen atom of the butyrolactone and the ring oxygen atom of the furanone moiety also display positive contributions to bioactivity so that the impact of these two partial structures is reflected in the HQSAR model as in the other models. The former, representing a potential H-bond acceptor activating the reactive β-carbon of the α,β-unsaturated enone system (and corresponding to pharmacophore feature F2 above), appears to be of positive influence on activity, which is in line with its potential influence on the reactivity of the conjugated Michael acceptor structure. However, this appears to contradict its negative contribution to the pharmacophore QSAR models discussed above. A strong positive contribution is also noted for the tigloyloxy moiety of **73**. This might be due to the presence of an additional α,β-unsaturated carbonyl group, or on the possibility for steric/hydrophobic interactions in this part of the molecule, which would be in line with the result of the pharmacophore-based QSAR where this moiety is represented by feature F3–5 (Models 3–5, see above). In Compound **1**, the second most active compound, as in **73**, the carbonyl oxygen atom in the enone system of the butyrolactone ring is also assigned a positive contribution. Besides this, atoms in the cycloheptane ring show a positive contribution that might simply reflect the fact that many compounds of the pseudoguaianolide series have a relatively high activity. The contribution maps of the two least active compounds, eudesmanolides **28** and **52** in this model, quite interestingly, do not indicate an influence of their lactone carbonyl oxygens, as opposed to the highly active compounds. This is in agreement with the fact that they have saturated γ-lactone moieties with methyl groups instead of the reactive exocyclic methylene. This observation is interesting in comparison with the apparent negative effect of this oxygen in the pharmacophore-based QSAR models. Obviously, the HQSAR model differentiates more easily between the α,β-unsaturated lactones and the saturated congeners, which is also straightforward to explain since the method is based on fragments of the molecular graph consisting of 4–7 atoms and not so much on the properties of particular atoms/groups. It is also interesting to note that atoms of the 6-membered rings of the weak trypanocides **28** and **52** appear to contribute negatively to anti-*Tbr* activity, which might reflect the fact that the eudesmanolides among the set of investigated compounds with their decalin or related core structures are generally weaker in this activity than most other subclasses of STLs.

**Figure 9:**
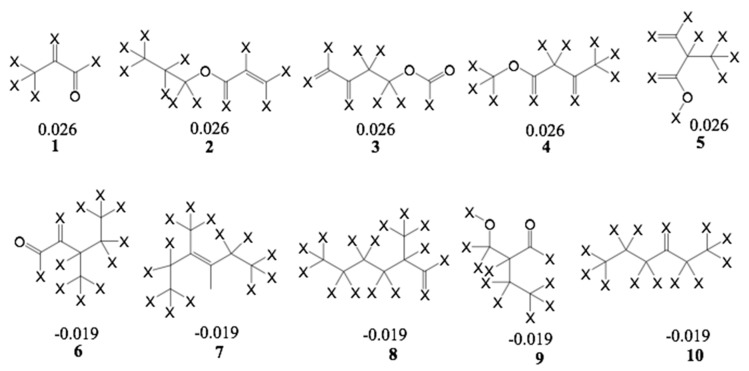
List of fragments with highest positive (**1**–**5**) and negative contribution (**6**–**10**) to HQSAR Model 8; X atoms are the connectivity flag and are not considered part of fragment. Note that the depicted double bond geometries do not indicate absolute (E/Z) configuration.

Furthermore, an analysis of statistical influence of some of the fragments with the highest positive and negative contributions on bioactivity was performed. The fragments are shown in Figure 9 for the A/C/Ch HQSAR Model 8.

From Fragments 1–3, it is evident that there are two adjacent sp^2^ carbon atoms, which is characteristic of an α,β-unsaturated carbonyl system. Fragments 1 and 2 encode part of the butyrolactone moiety. These fragments, together with some others yielded the highest positive contribution to the model. Surprisingly, Fragment 6 contains an α,β-unsaturated carbonyl system but shows a negative contribution to the model. It is not quite clear which structural element this fragment represents but since some compounds of low activity, e.g., **52**, also contain enone systems (probably of low reactivity), this finding is also not altogether contradictory to the rest of the models. However, among the other fragments with negative impact on activity, α,β-unsaturated carbonyl systems are absent.

Overall, the HQSAR method provided models of similar internal predictive quality as the other two QSAR approaches. They are more difficult to interpret, due to the complex nature of their generation but yield significantly better external predictions. It may be worth mentioning that HQSAR is a 2D QSAR method that does not require 3D optimization and is hence faster than the other methods applied.

## 3. Materials and Methods

Compounds **1**–**73** were as used in a previous QSAR study [9], and **74**–**118** were recently obtained, identified, and their activity determined within our working group [5,6,7,24]. Compound **79** was isolated from *Dimerostemma* sp. [24], Compounds **80**–**82** from *Calea clausseniana* [24], Compounds **83** and **84** from *Aspilia* sp. [24], Compounds **85** and **86** from *Lepidaploa rufogrisea* [24], Compound **87** from *Tarchonanthus camphoratus* [5], Compounds **88**–**107** from *Schkuhria pinnata* [5], Compounds **108**–**113** from *Vernonia lasiopus* [7], and **114**–**118** from *V. cinerascens* [6]. The activity data of **119**–**130**, all isolated from *Anthemis nobilis*, was extracted from literature having been determined under the same conditions at the Swiss Tropical and Public Health Institute (STPH) laboratory [25] (see Figure 1 for all structures). Three-dimensional (3D) molecular models of the 130 STLs were generated with Molecular Operating Environment (MOE; version 2016.8; Chemical Computing Group, Montreal, QC., Canada). The resulting geometries were then optimized using the MMFF94x force field, and MOE default settings were used to perform a stochastic conformational search for each compound. A maximum of 15 conformers within an energy window of 5.0 kcal/mol above the global minimum were retained for further study. The IC_50_ values of each of the compounds’ biological activity against *Tbr* and L6 cells, expressed in molar concentration, were converted to negative decadic logarithms (pIC_50_; Table S2).

“*Classical*” *descriptor-based QSAR approach—Models 1 and 2*: The resulting conformers with the lowest force field energy were minimized using the AM1 Hamiltonian (MOPAC module of MOE), and the minimized geometries thus obtained were used in the QSAR study for Model 1. The QSAR module of MOE was then used to calculate molecular descriptors (123) for each of the 130 energy minimized structures (Table S1). The data set was divided into a training (*n* = 87) and a test (*n* = 43) set by randomly selecting 43 molecules to constitute the test set (see Table S3).

In order to include the aspect of molecular flexibility in the QSAR modeling, a second model, herein referred to as Model 2, was developed with Boltzmann-weighted descriptor averages of the molecules. Here, the conformations of all the 130 molecules previously obtained through a stochastic conformational search were energy-minimized using the AM1 Hamiltonian as formerly explained. The same 123 molecular descriptors used in Model 1 were then calculated for each of the conformers. Boltzmann-weighted descriptor values were then calculated for each compound by calculating an average for the various conformers weighted by their ΔH_f_ values using the Boltzmann equation [26]:

(1) Pi=e−ΔEiRT∑i=1te−ΔEiRT

where *P* is the probability of finding a compound in the particular state *i* in an equilibrium of *t* states (conformations) at a given absolute temperature T, which is related to the energy difference of state *i* from the global minimum ΔE. R is the universal gas constant. The STL structures were then divided into a training set (*n* = 90) and a test set (*n* = 40) in a random manner.

The QSAR modeling was then done using the genetic algorithm-driven variable selection combined with multiple linear regression (GA-MLR, MOE script GA.svl available at the CGC/MOE svl exchange website [http://www.chemcomp.com/Support-SVL_Exchange.htm]). This algorithm optimizes the performance in MLR for a family or population of descriptor combinations. In a given combination, each descriptor represents a “gene” within the model. “Mutation” of models is simulated by randomly exchanging individual genes for new ones in a predefined number of models. “Crossing over” events, that is, the exchange of more than one descriptor (gene groups) among already existing models, also occur. A large number of “mutation” or “crossing over” progressions lead to the evolution of a population. After each series of evolution, re-evaluation is done and models with poor performance are eliminated, i.e., become “extinct.” This leads to an optimized population with respect to performance, measured by a parameter of choice. The optimization criterion chosen in this study was a minimization in the lack of fit (LOF) in the MLR of the descriptors versus the pIC_50_. The number of variables in each model was fixed to values of 5, 6, and 7 variables; i.e., a GA run was performed with each of these model sizes. The population size in all GA runs was set to 100 equations and the full descriptor matrix. The termination criteria for a GA run were either that a predefined LOF or that a maximum of 1000 evolution cycles had been reached. All the populations optimized were cross-validated using the leave-one-out method, and the resulting equations were ranked by their cross-validated coefficient of determination (Q^2^).

*Pharmacophore feature-based QSAR approach—Models 3–5*: For the pharmacophore based Model 3, the method described by Schomburg et al. [13] was adopted. Briefly, the structural comparison of Compounds **2**, **73**, **97**, and **111** (Figure 3a), representing the most active compounds of the different classes of STLs in this series, was carried out using the default settings of the flexible alignment function in MOE [27]. Thereafter, the MOE pharmacophore query editor was used to generate the pharmacophore model consisting of the pharmacophore features shown in Figure 3b. For the quantum mechanical calculations and visualization of the lowest unoccupied frontier molecular orbitals (LUMO, LUMO+1, and LUMO+2) in the lowest energy conformers of the most active Compounds **2**, **73**, **97**, and **111**, the Gaussian 03W software [28] was used. First, the structures were optimized using the semi-empirical AM1 Hamiltonian and the resulting geometries were then optimized further using density functional theory (DFT) at the B3LYP [29] (combination of Becke’s three parameter exchange functional (B3) [30] and Lee–Yang–Parr (LYP) correlation functional [31]) level using the 6-31G (d,p) basis set [32].

The force field potential of the superposed ensemble of **2**, **73**, **97**, and **111** was fixed (i.e., no further change of coordinates allowed for these molecules), and each of the other structures was then aligned with this template using the flexible alignment function. Each overlaid structure was visually matched with the pharmacophore model and binary indicator variables were generated, which were assigned a value of 0 or 1 if the molecule lacked or possessed a particular feature, respectively. Consequently, 10 indicator variables were obtained: *F1*: Michael acceptor carbon corresponding to reactive β-exomethylene of methylene lactone group; *F2*: H-bond acceptor corresponding to carbonyl oxygen of methylene lactone group; *F3*: hydrophobic feature corresponding to methacryloyl moiety in **111**; *F4*: H-bond acceptor corresponding to ester carbonyl moiety; *F5*: hydrophobic feature corresponding to acetyl, methacryloyl, and 3-furoloxy moieties in **2**, **73**, and **97**, respectively; *F6*: hydrophobic feature corresponding to CH_3_-15 in **73** and **97**, exomethylene in **111**, and CH_3_-14 in **2**; *F7*: Michael acceptor carbon corresponding to reactive β-exomethylene of methylene pyranone structure in **111**; *F8*: Michael acceptor carbon corresponding to the reactive δ-position of α,β,γ,δ-unsaturated ketone structure in **73**; *F9*: Michael acceptor carbon corresponding to the reactive β-position of cyclopentenone in **2**; *F10*: H-bond acceptor corresponding to the carbonyl oxygen of cyclopentenone (**2**) and furanone (**73**) moiety. These indicator variables and the global alignment score S, a measure of the fit of the molecular structure to the alignment of **2**, **73**, **97**, and **111**, were used as the QSAR descriptors. The molecules were randomly divided into a training set (*n* = 86) and a test set (*n* = 44), ensuring that both sets of compounds covered evenly the entire range of biological activities. Partial least squares (PLS) regression as implemented in MOE [27] was used to analyze the correlation of the descriptor matrix of the training set with biological activity, starting with the pharmacophore descriptors (F1–F10,S).

Finally, Model 5 was developed by the combination of the pharmacophore descriptors with the 123 molecular descriptors obtained with the lowest force field energy structures. Before this, the number of pharmacophore descriptors was reduced by combining features encoding closely related structural elements. F3 and F5 into a new feature F3–5 and the features F7 and F8 into F7–8. This reduction in descriptor variables did not have a significant influence on the statistics (see results of Model 4). Then GA/MLR analysis was used for QSAR modeling of this combined descriptor matrix using a fixed length of 6 descriptor variables.

*Hologram-QSAR (HQSAR) approach—Model 6*: The HQSAR method was applied using the Sybyl X 2.0 software package from Tripos Inc., St. Louis, Mo, USA. In HQSAR, each molecule in the dataset is broken down into all possible linear, branched, cyclic, and overlapping fragments. Each of the fragments is then assigned a pseudo-random (positive integer value) using a cyclic redundancy check algorithm. Subsequently, the generated fragments are arranged to form a molecular hologram, a linear array of integers containing counts of molecular fragments, which is consequently divided into fixed-length arrays (53 to 401 bins, yielding various holograms of different lengths). The bin occupancies simply represent counts of fragments in each bin. The bins are then used as molecular descriptors encoding topological and compositional molecular information. The generation of hologram and the subsequent analysis of HQSAR models constructed is influenced by hologram length, fragment size, and fragment distinction, specifically atoms (A), bonds (B), connections (C), hydrogen atoms (H), chirality (Ch), and H-bond donor/acceptor groups (DA). The partial least squares (PLS) regression method is then used to generate models, which are validated by leave-one-out cross validation as in the other QSAR approaches.

In the present case, the set of compounds was divided into the same training (*n* = 87) and test set (*n* = 43) as in Model 1 described above. Twelve default hologram lengths (53, 59, 61, 71, 83, 97, 151, 199, 257, 307, 353, and 401) were evaluated. After initial analysis with a fragment size of 4–7 atoms, the impact of fragment size was examined also for other fragment sizes (see Table 3). The leave-one-out (LOO) method was used for internal cross-validation of each model. Thereafter, an external validation of the models was performed, as above, by predicting the activity of the test set molecules.

Models 1–8 were subjected to statistical tests as proposed by Golbraikh and Tropsha [33,34] (see Table S7). Each of these models was furthermore subjected to five independent Y-scrambling tests; i.e., the biological activity data were randomly re-assigned to the compounds of the training sets and the GA/MLR variable selection and regression analyses repeated (Models 1–5). None of the QSAR equations resulting from these tests showed a significant correlation in terms of R^2^, Q^2^, or RMSE (see Table S8). In case of Models 6–8, Y-scrambling validation was performed by 50 Y-scrambling experiments with the same experimental parameters as explained above. In all cases low values of R^2^ and Q^2^ were obtained, with average of 0.656–0.485 (A/C/H/Ch; Model 6), 0.646–0.440 (A/C; Model 7), and 0.746–0.575 (A/C/Ch; Model 8), respectively (see Figure S4). Additionally, applicability domain (AD) calculations for Models 1–5 were carried out employing the basic theory of the standardization approach using the standalone application tool developed by Roy et al. [35]. The test set compounds were all within the applicability domains of these models, with the exception of Compound **90** in Model 1 and Compound **91** in Models 3 and 4 (see Table S9). AD calculations for Models 6–8 were performed with the HQSAR module of Sybyl. The results are shown in graphic form in Figure S5, where it becomes evident that test set compounds, in all cases, are distributed well within the range of the training set.

## 4. Conclusions

The present study provides QSAR models obtained with three different modeling approaches but of quite comparable statistical quality in terms of calibration, cross validation, and external predictions. Certainly, the models presented here will be applicable only within the wide chemical space of STLs, a class of natural products with more than 5000 known representatives. It is interesting to note that each of these approaches in its own way points toward the prime importance of reactive structure elements for the antitrypanosomal activity of STLs. Since this study is based on a structurally much more diverse set of compounds than previous studies, it may be expected that predictions of this activity for untested compounds may be even more reliable than those obtained with previous QSAR models. With regard to predictions for large virtual libraries, it is clear that the HQSAR approach, based only on molecular graph information and yielding a higher level of external predictivity, will be superior in terms of computational effort. The pharmacophore-based QSAR approach, however, has the great advantage of being more straightforward to interpret. By the models obtained with this methodology, it is very clearly shown that not only the presence but also the relative position and orientation of reactive enone structure elements is of high importance for the antitrypanosomal activity of STLs.

## Figures and Tables

**Table 1:** The 10 best GA/MLR QSAR equations obtained in Model 1.

No.	QSAR Equations	R^2^	Q^2^	RMSE	RMSEP
1	pIC_50_(*Tbr*) = 11.9040 + 0.0255 ASAP4 + 0.0270 ENONCS − 9.1834 FASA^+^ + 2.1450 npr1 − 1.6691 vsurf_CW2 + 0.6144 vsurf_ID2	0.72	0.67	0.46	0.51
2	pIC_50_(*Tbr*) = 12.0792 + 0.0255 ASAP4 + 0.02719 ENONCS − 9.3959 FASA^+^ + 2.1827 npr1 − 1.6946 vsurf_CW2 + 0.5937 vsurf_ID1	0.72	0.67	0.46	0.51
3	pIC_50_(*Tbr*) = 27.8764 + 0.0259 ASAP4 + 0.0249 ENONCS − 10.5589 FASA^+^ − 0.7813 std_dim1 − 5.7580 vsurf_CW1 + 0.6569 vsurf_ID2	0.72	0.67	0.47	0.51
4	pIC_50_(*Tbr*) = 31.6187 + 0.0262 ASAP4 + 0.0238 ENONCS − 12.7113 FASA^+^ − 0.8864 rgyr − 6.3527 vsurf_CW1 + 0.6624 vsurf_ID2	0.71	0.67	0.47	0.51
5	pIC_50_(*Tbr*) = 17.7687 + 0.0265 ASAP4 + 0.0248 ENONCS + 10.6761 FASA^−^ − 0.7938 std_dim1 − 5.9248 vsurf_CW1 + 0.6439 vsurf_ID2	0.71	0.67	0.47	0.51
6	pIC_50_(*Tbr*) = 2.7606 + 0.0261 ASAP4 + 0.0273 ENONCS + 9.0020 FASA^−^+ 2.2131 npr1 − 1.6664 vsurf_CW2 + 0.6031 vsurf_ID2	0.71	0.66	0.47	0.51
7	pIC_50_(*Tbr*) = 13.0177 + 0.0254 ASAP4 + 0.0253 ENONCS −9.8955 FASA^+^ + 2.4896 glob − 1.8135 vsurf_CW2 + 0.6452 vsurf_ID2	0.71	0.66	0.47	0.52
8	pIC_50_(*Tbr*) = 17.9407 + 0.0263 ASAP4 + 0.0247 ENONCS + 10.8244 FASA^−^ − 0.7663 std_dim1 − 6.0230 vsurf_CW1 + 0.5935 vsurf_ID1	0.71	0.66	0.47	0.52
9	pIC_50_(*Tbr*) = 19.2251 + 0.0268 ASAP4 + 0.0238 ENONCS + 12.6357 FASA^−^ − 0.8739 rgyr − 6.4677 vsurf_CW1 + 0.6436 vsurf_ID2	0.71	0.66	0.47	0.52
10	pIC_50_(*Tbr*) = 7.0547 + 0.0299 ASAP4 − 0.0083 DASA + 0.0313 ENONCS + 2.6955 npr1 − 1.8094 vsurf_CW2 + 0.6920 vsurf_ID2	0.71	0.66	0.47	0.52

*n* = 87, Data were standardized to unit variance.

**Table 2:** The 10 best GA/MLR QSAR equations obtained in Model 2.

No.	QSAR Equations	R^2^	Q^2^	RMSE	RMSEP
1	pIC_50_(*Tbr*) = −1.4803 + 0.0169 ASAP4 + 0.0135 ENONCS + 12.3791 FASA^−^ − 2.1775 vsurf_CW2 − 1.1349 vsurf_EWmin3 − 1.5861 vsurf_HB8	0.71	0.67	0.47	0.50
2	pIC_50_(*Tbr*) = −1.4801 + 0.0169 ASAP4 + 0.0135 ENONCS + 12.3791 FASA^−^ − 2.1773 vsurf_CW2 − 1.1348 vsurf_EWmin3 − 1.5859 vsurf_W8	0.71	0.67	0.47	0.50
3	pIC_50_(*Tbr*) = 3.3263 + 0.0171 ASAP4 − 0.0227 ASAP6 + 0.0109 ENONCS + 13.9402 FASA^−^ − 2.0114 vsurf_CW2 + 0.5432 vsurf_ID2	0.70	0.66	0.47	0.51
4	pIC_50_(*Tbr*) = 3.8321 + 0.0168 ASAP4 − 0.0178 ASAP6 + 0.0127 ENONCS + 13.4613 FASA^−^ − 2.1945 vsurf_CW2 + 0.3399 vsurf_ID7	0.70	0.66	0.47	0.51
5	pIC_50_(*Tbr*) = 10.9 + 0.0171 ASAP4 + 0.0136 ENONCS − 12.2378 FASA^+^ − 2.2036 vsurf_CW2 − 1.1269 vsurf_EWmin3 − 1.5749 vsurf_HB8	0.70	0.66	0.47	0.51
6	pIC_50_(*Tbr*) = 17.2454 + 0.0173 ASAP4 −0.0224 ASAP6 + 0.0110 ENONCS − 13.813 FASA^+^ − 2.0431 vsurf_CW2 + 0.5418 vsurf_ID2	0.70	0.66	0.47	0.51
7	pIC_50_(*Tbr*) = 3.9667 + 0.0154 ASAP4 + 0.0155 ENONCS + 12.5953 FASA^−^ − 2.1772 vsurf_CW2 − 0.211 vsurf_HB8 + 0.3704 vsurf_ID7	0.70	0.65	0.47	0.51
8	pIC_50_(*Tbr*) = 3.9667 + 0.0154 ASAP4 + 0.0155 ENONCS + 12.5954 FASA^−^ − 2.1771 vsurf_CW2 + 0.3704 vsurf_ID7 − 0.2107 vsurf_W8	0.70	0.65	0.47	0.51
9	pIC_50_(*Tbr*) = 17.264 + 0.0170 ASAP4 − 0.0176 ASAP6 + 0.0129 ENONCS−13.3265 FASA^+^ − 2.22422 vsurf_CW2 + 0.339 vsurf_ID7	0.70	0.65	0.47	0.51
10	pIC50(*Tbr*) = 3.52157 + 0.0168 ASAP4 − 0.0197 ASAP6 + 0.0109 ENONCS + 14.0233 * FASA^−^ − 2.1072 vsurf_CW2 + 0.3390 vsurf_ID6	0.70	0.65	0.47	0.51

*n* = 90, Data were standardized to unit variance.

**Table 3:** Statistical characteristics of HQSAR models with different fragment distinction schemes and fragment size variations.

F_dist_	F_size_ (Atoms)	Q^2^	SEV	R^2^	SEE	HL	PC	SEEP	*P* ^2^
A/C/H/Ch (Model 6)	2–5	0.55	0.61	0.74	0.46	199	5	-	-
	3–6	0.59	0.58	0.80	0.40	71	6	-	-
	**4**–**7**	**0.63**	**0.55**	**0.85**	**0.36**	**71**	**6**	**0.39**	**0.66**
	5–8	0.59	0.58	0.85	0.35	199	6	-	-
	6–9	0.52	0.62	0.89	0.30	401	6	-	-
A/C (Model 7)	2–5	0.56	0.60	0.75	0.46	199	5	-	-
	3–6	0.59	0.58	0.81	0.39	401	6	-	-
	**4**–**7**	**0.62**	**0.56**	**0.84**	**0.37**	**401**	**6**	**0.36**	**0.73**
	5–8	0.57	0.59	0.82	0.38	401	5	-	-
	6–9	0.57	0.58	0.83	0.38	257	5	-	-
A/C/Ch (Model 8)	2–5	0.59	0.58	0.76	0.44	199	5	-	-
	3–6	0.62	0.56	0.81	0.40	71	6	-	-
	**4**–**7**	**0.62**	**0.56**	**0.81**	**0.39**	**199**	**5**	**0.38**	**0.68**
	5–8	0.58	0.56	0.84	0.36	199	5	-	-
	6–9	0.60	0.58	0.84	0.36	353	5	-	-

F_dist_: fragment distinction; F_size_: fragment size; HL: hologram length; PC: number of PLS principal components; SEV: standard error of validation; SEE: standard error of estimation; SEEP: standard error of estimation of test set. Abbreviations in fragment distinction schemes: A = Atoms; C = Connectivity; H = Hydrogens; Ch = Chirality. Bold: The best PLS models obtained with A/C/H/Ch, A/C, and A/C/Ch F_dist_ schemes and F_size_ of 4–7 atoms.

## Supplementary Materials

Supplementary materials can be found at http://www.mdpi.com/1422-0067/19/12/3721/s1.
