# Supplementary material for: Complementary Quantitative Structure–Activity Relationship Models for the Antitrypanosomal Activity of Sesquiterpene Lactones
Source: Int J Mol Sci. 2018 Nov 22;19(12):3721. doi: 10.3390/ijms19123721 (PMC6321053; doi:10.3390/ijms19123721)
Supplement: Supplementary file 1 [file ijms-19-03721-s001.pdf]

# Complementary quantitative structure-activity relationship models for the antitrypanosomal activity of sesquiterpene lactones

**Njogu M. Kimani [1], Josphat C. Matasyoh [2], Marcel Kaiser [3,4], Gustavo H. G. Trossini [5] and Thomas J. Schmidt [1,] ***

[1] [1]Institute of Pharmaceutical Biology and Phytochemistry (IPBP), University of Muenster, PharmaCampus Corrensstrasse 48, Muenster D-48149, Germany; Email: m_kima01@uni-muenster.de
[2] Department of Chemistry, Egerton University, P.O. Box 536, Egerton 20115, Kenya; Email: josphat2001@yahoo.com
[3] Swiss Tropical and Public Health Institute (Swiss TPH), Socinstrasse 57, Basel CH-4051, Switzerland
[4] University of Basel, Petersplatz 1, Basel CH-4003, Switzerland; Email: marcel.kaiser@unibas.ch
[5] Faculdade de Ciências Farmacêuticas, Universidade de São Paulo, Av. Lineu Prestes, 580, 05508-000 São Paulo, Brazil; Email: trossini@usp.br
* Correspondence: thomschm@uni-muenster.de; Tel.: +49-251-83-33378

Received: date; Accepted: date; Published: date

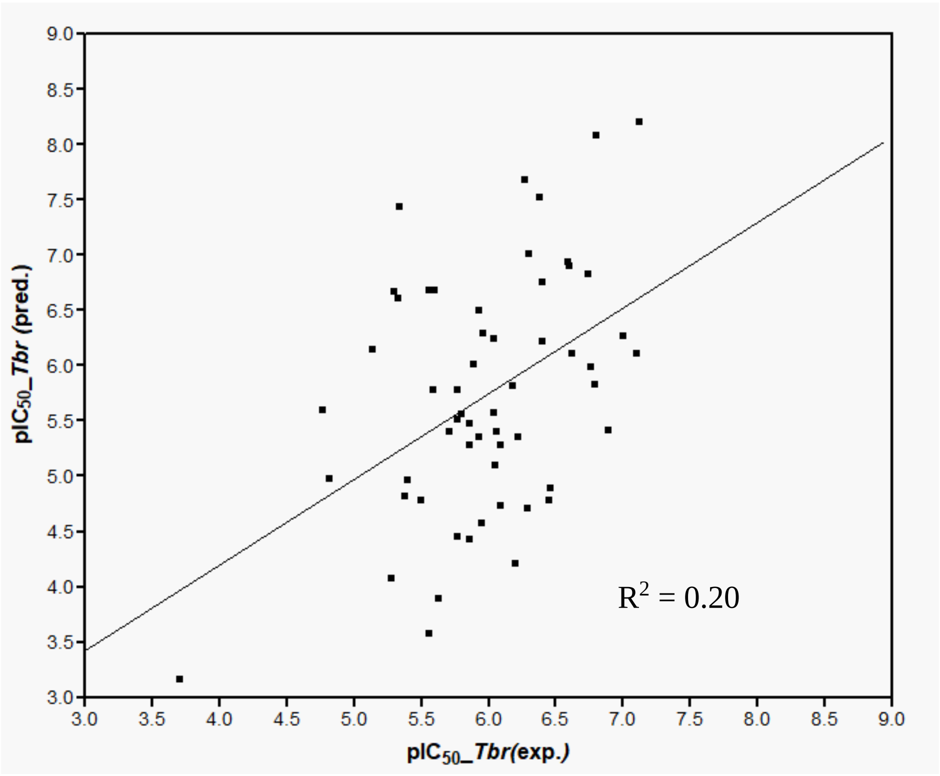


Figure S1: Plot of predicted versus experimental $pIC_{50}$ values on the newly tested molecules as obtained from the QSAR model developed in 2014 [9]

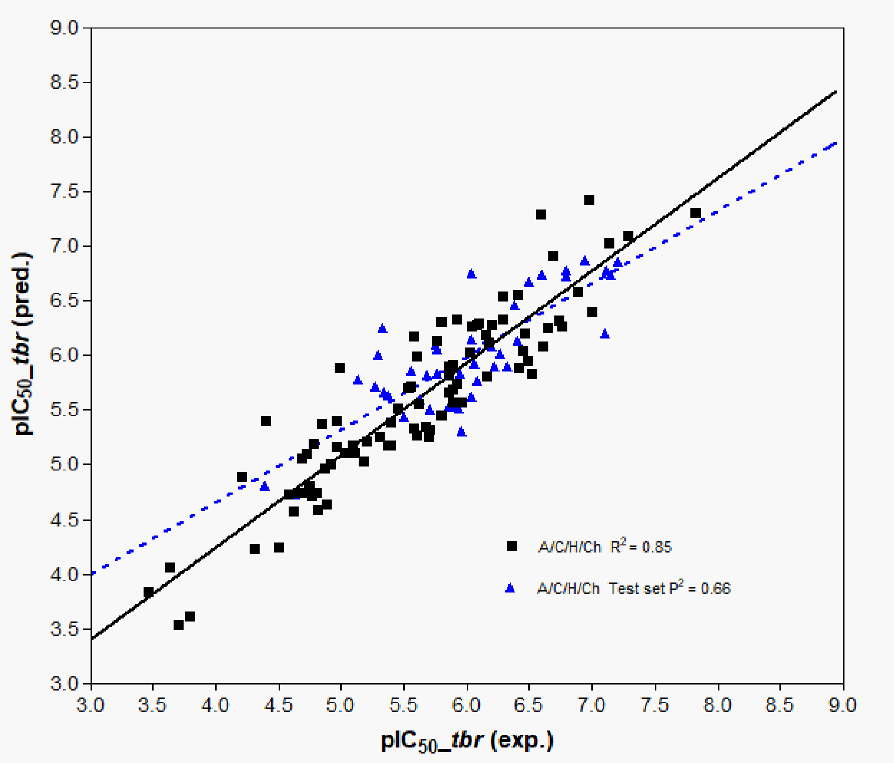


Figure S2: Experimental versus predicted $pIC_{50}$ values of training and test sets of the A/C/H/Ch HQSAR model (model 6)

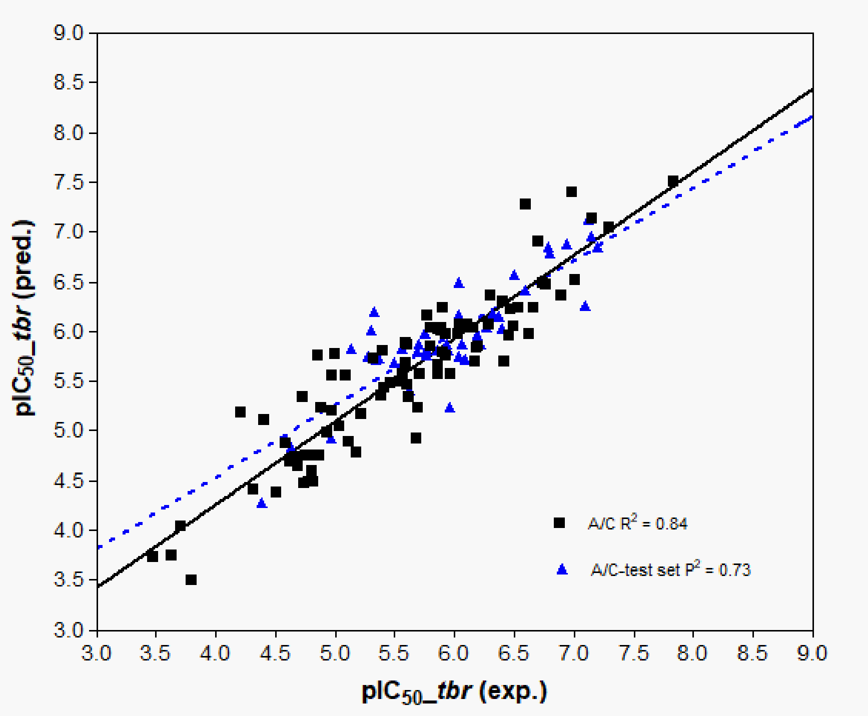


Figure S3: Experimental versus predicted $pIC_{50}$ values of training and test sets of the A/C HQSAR model (model 7)

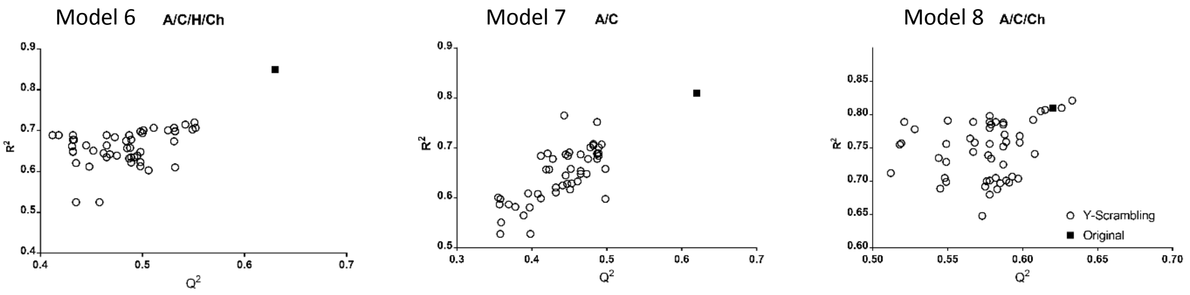


Figure S4: Plots of $Q^2$ versus $R^2$ values of the 50 Y-scrambling experiments for HQSAR models 6-8

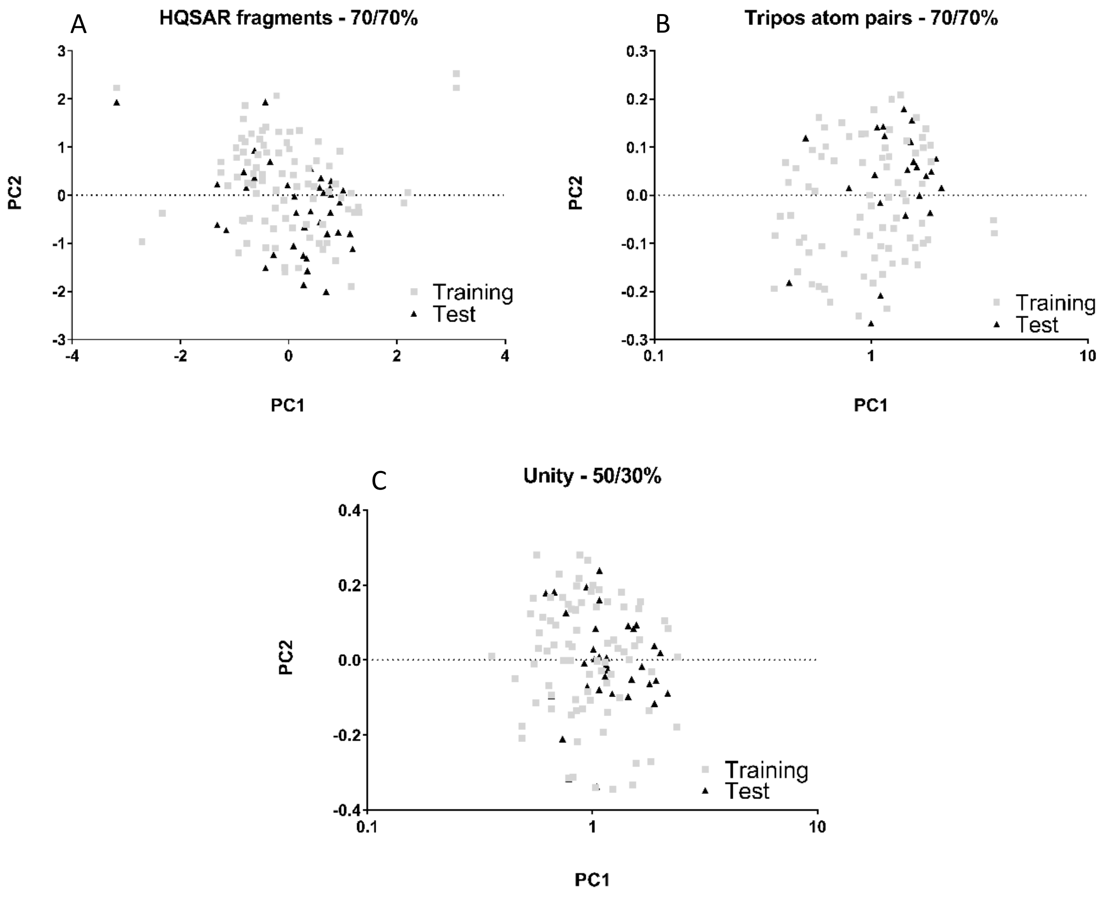


Figure S5: Applicability domain (AD) calculations for the HQSAR models 6-8. The plots show results of principal component analyses for three different descriptor schemes used by HQSAR to describe the molecules: A: Based on fingerprints from HQSAR models; B: Tripos atom pairs – based on Sybyl atom types used to generate fingerprints; C "Unity" – related to functional groups). In each case, the test set compounds are distributed well within the range of the training set. This suggests the correctness of the applicability domain and that training and test set separation for the models are reasonable.

Table S1: Molecular descriptors used in the QSAR studies

| CODE | CLASS | DESCRIPTION |
|---|---|---|
| AM1_dipole | i3D | Dipole moment |
| AM1_HOMO | i3D | HOMO energy (eV) |
| AM1_LUMO | i3D | LUMO energy (eV) |
| ASA | i3D | Water accessible surface area |
| ASA+ | i3D | Positive accessible surface area |
| ASA- | i3D | Negative accessible surface area |
| ASAN1 | i3D | Fract. neg accessible surface area 1 |
| ASAN2 | i3D | Fract. neg accessible surface area 2 |
| ASAN3 | i3D | Fract. neg accessible surface area 3 |
| ASAN4 | i3D | Fract. neg accessible surface area 4 |
| ASAN5 | i3D | Fract. neg accessible surface area 5 |
| ASAN6 | i3D | Fract. neg accessible surface area 6 |
| ASAN7 | i3D | Fract. neg accessible surface area 7 |
| ASAP1 | i3D | Fract. pos accessible surface area 1 |
| ASAP2 | i3D | Fract. pos accessible surface area 2 |
| ASAP3 | i3D | Fract. pos accessible surface area 3 |
| ASAP4 | i3D | Fract. pos accessible surface area 4 |
| ASAP5 | i3D | Fract. pos accessible surface area 5 |
| ASAP6 | i3D | Fract. pos accessible surface area 6 |
| ASAP7 | i3D | Fract. pos accessible surface area 7 |
| ASA_H | i3D | Total hydrophobic surface area |
| ASA_P | i3D | Total polar surface area |
| CASA+ | i3D | Charge-weighted positive surface area |
| CASA- | i3D | Charge-weighted negative surface area |
| DASA | i3D | Absolute difference in surface area |
| DCASA | i3D | Absolute difference in charge-weighted areas |
| dens | i3D | Mass density (AMU/A^3) |
| dipole | i3D | Dipole moment |
| ENONCS | 2D | ASA of enon beta carbon |
| ENONS | 2D | ASA of enon groups |
| FASA+ | i3D | Fractional positive accessible surface area |
| FASA- | i3D | Fractional negative accessible surface area |
| FASA_H | i3D | Fractional hydrophobic surface area |
| FASA_P | i3D | Fractional polar surface area |
| FCASA+ | i3D | Fractional charge-weighted positive surface area |
| FCASA- | i3D | Fractional charge-weighted negative surface area |
| glob | i3D | Molecular globularity |
| logP(o/w) | 2D | Log octanol/water partition coefficient |

| | | |
|---|---|---|
| npr1 | i3D | Normalized PMI ratio (1) (pmi1 / pmi3) |
| npr2 | i3D | Normalized PMI ratio (2) (pmi2 / pmi3) |
| rgyr | i3D | Radius of gyration |
| std_dim1 | i3D | Standard dimension 1 |
| std_dim2 | i3D | Standard dimension 2 |
| std_dim3 | i3D | Standard dimension 3 |
| vol | i3D | Van der Waals volume |
| VSA | i3D | Van der Waals surface area |
| vsurf_A | i3D | Amphiphilic moment |
| vsurf_CP | i3D | Critical packing parameter |
| vsurf_CW1 | i3D | Capacity factor at -0.2 |
| vsurf_CW2 | i3D | Capacity factor at -0.5 |
| vsurf_CW3 | i3D | Capacity factor at -1.0 |
| vsurf_CW4 | i3D | Capacity factor at -2.0 |
| vsurf_CW5 | i3D | Capacity factor at -3.0 |
| vsurf_CW6 | i3D | Capacity factor at -4.0 |
| vsurf_CW7 | i3D | Capacity factor at -5.0 |
| vsurf_CW8 | i3D | Capacity factor at -6.0 |
| vsurf_D1 | i3D | Hydrophobic volume at -0.2 |
| vsurf_D2 | i3D | Hydrophobic volume at -0.4 |
| vsurf_D3 | i3D | Hydrophobic volume at -0.6 |
| vsurf_D4 | i3D | Hydrophobic volume at -0.8 |
| vsurf_D5 | i3D | Hydrophobic volume at -1.0 |
| vsurf_D6 | i3D | Hydrophobic volume at -1.2 |
| vsurf_D7 | i3D | Hydrophobic volume at -1.4 |
| vsurf_D8 | i3D | Hydrophobic volume at -1.6 |
| vsurf_DD12 | i3D | vsurf_EDmin1, vsurf_EDmin2 distance |
| vsurf_DD13 | i3D | vsurf_EDmin1, vsurf_EDmin3 distance |
| vsurf_DD23 | i3D | vsurf_EDmin2, vsurf_EDmin3 distance |
| vsurf_DW12 | i3D | vsurf_EWmin1, vsurf_EWmin2 distance |
| vsurf_DW13 | i3D | vsurf_EWmin1, vsurf_EWmin3 distance |
| vsurf_DW23 | i3D | vsurf_EWmin2, vsurf_EWmin3 distance |
| vsurf_EDmin1 | i3D | Lowest hydrophobic energy |
| vsurf_EDmin2 | i3D | 2nd lowest hydrophobic energy |
| vsurf_EDmin3 | i3D | 3rd lowest hydrophobic energy |
| vsurf_EWmin1 | i3D | Lowest hydrophilic energy |
| vsurf_EWmin2 | i3D | 2nd lowest hydrophilic energy |
| vsurf_EWmin3 | i3D | 3rd lowest hydrophilic energy |
| vsurf_G | i3D | Surface globularity |
| vsurf_HB1 | i3D | H-bond donor capacity at -0.2 |
| vsurf_HB2 | i3D | H-bond donor capacity at -0.5 |

| | | |
|---|---|---|
| vsurf_HB3 | i3D | H-bond donor capacity at -1.0 |
| vsurf_HB4 | i3D | H-bond donor capacity at -2.0 |
| vsurf_HB5 | i3D | H-bond donor capacity at -3.0 |
| vsurf_HB6 | i3D | H-bond donor capacity at -4.0 |
| vsurf_HB7 | i3D | H-bond donor capacity at -5.0 |
| vsurf_HB8 | i3D | H-bond donor capacity at -6.0 |
| vsurf_HL1 | i3D | First hydrophilic-lipophilic balance |
| vsurf_HL2 | i3D | Second hydrophilic-lipophilic balance |
| vsurf_ID1 | i3D | Hydrophobic integy moment at -0.2 |
| vsurf_ID2 | i3D | Hydrophobic integy moment at -0.4 |
| vsurf_ID3 | i3D | Hydrophobic integy moment at -0.6 |
| vsurf_ID4 | i3D | Hydrophobic integy moment at -0.8 |
| vsurf_ID5 | i3D | Hydrophobic integy moment at -1.0 |
| vsurf_ID6 | i3D | Hydrophobic integy moment at -1.2 |
| vsurf_ID7 | i3D | Hydrophobic integy moment at -1.4 |
| vsurf_ID8 | i3D | Hydrophobic integy moment at -1.6 |
| vsurf_IW1 | i3D | Hydrophilic integy moment at -0.2 |
| vsurf_IW2 | i3D | Hydrophilic integy moment at -0.5 |
| vsurf_IW3 | i3D | Hydrophilic integy moment at -1.0 |
| vsurf_IW4 | i3D | Hydrophilic integy moment at -2.0 |
| vsurf_IW5 | i3D | Hydrophilic integy moment at -3.0 |
| vsurf_IW6 | i3D | Hydrophilic integy moment at -4.0 |
| vsurf_IW7 | i3D | Hydrophilic integy moment at -5.0 |
| vsurf_IW8 | i3D | Hydrophilic integy moment at -6.0 |
| vsurf_R | i3D | Surface rugosity |
| vsurf_S | i3D | Interaction field area |
| vsurf_V | i3D | Interaction field volume |
| vsurf_W1 | i3D | Hydrophilic volume at -0.2 |
| vsurf_W2 | i3D | Hydrophilic volume at -0.5 |
| vsurf_W3 | i3D | Hydrophilic volume at -1.0 |
| vsurf_W4 | i3D | Hydrophilic volume at -2.0 |
| vsurf_W5 | i3D | Hydrophilic volume at -3.0 |
| vsurf_W6 | i3D | Hydrophilic volume at -4.0 |
| vsurf_W7 | i3D | Hydrophilic volume at -5.0 |
| vsurf_W8 | i3D | Hydrophilic volume at -6.0 |
| vsurf_Wp1 | i3D | Polar volume at -0.2 |
| vsurf_Wp2 | i3D | Polar volume at -0.5 |
| vsurf_Wp3 | i3D | Polar volume at -1.0 |
| vsurf_Wp4 | i3D | Polar volume at -2.0 |
| vsurf_Wp5 | i3D | Polar volume at -3.0 |
| vsurf_Wp6 | i3D | Polar volume at -4.0 |

| | | |
|---|---|---|
| vsurf_Wp7 | i3D | Polar volume at -5.0 |
| vsurf_Wp8 | i3D | Polar volume at -6.0 |
| Weight | 2D | Molecular weight (CRC) |

Table S2: Bio-activity data of STLs used in QSAR modelling

| No. | pIC50_Tbr | pIC50_L6 | No. | pIC50_Tbr | pIC50_L6 | No. | pIC50_Tbr | pIC50_L6 |
|---|---|---|---|---|---|---|---|---|
| **1** | 7.2840 | 6.0030 | **45** | 4.6840 | 4.0610 | **89** | 5.5817 | 4.4675 |
| **2** | 7.2010 | 6.0920 | **46** | 6.5230 | 5.6780 | **90** | 5.2688 | 4.2865 |
| **3** | 6.9790 | 5.9870 | **47** | 4.5820 | 4.2010 | **91** | 5.3936 | 4.3735 |
| **4** | 6.9360 | 5.8870 | **48** | 3.6300 | 3.5230 | **92** | 5.9431 | 5.1073 |
| **5** | 6.1640 | 5.0510 | **49** | 5.3680 | 5.2750 | **93** | 6.0862 | 4.9602 |
| **6** | 5.8490 | 5.5150 | **50** | 5.4490 | 4.9710 | **94** | 5.7645 | 4.2626 |
| **7** | 6.0400 | 5.3390 | **51** | 6.1640 | 5.2080 | **95** | 6.4559 | 5.3904 |
| **8** | 6.4960 | 5.6120 | **52** | 4.9890 | 3.8490 | **96** | 5.7670 | 5.1002 |
| **9** | 5.0330 | 4.9110 | **53** | 5.8930 | 5.0000 | **97** | 7.0000 | 5.6882 |
| **10** | 5.1740 | 5.3570 | **54** | 5.7460 | 4.8300 | **98** | 6.8861 | 5.4112 |
| **11** | 4.7360 | 4.4960 | **55** | 4.8770 | 4.6470 | **99** | 6.2840 | 5.1701 |
| **12** | 4.9610 | 4.7780 | **56** | 4.6130 | 4.2740 | **100** | 6.0410 | 4.8425 |
| **13** | 4.7160 | 4.4980 | **57** | 6.6480 | 5.3270 | **101** | 6.0362 | 4.8368 |
| **14** | 5.9300 | 5.0740 | **58** | 5.5980 | 5.2090 | **102** | 5.8539 | 5.1524 |
| **15** | 5.4020 | 5.1380 | **59** | 5.6090 | 5.1180 | **103** | 6.0362 | 5.4202 |
| **16** | 4.8660 | 4.6660 | **60** | 5.6810 | 5.3110 | **104** | 3.7029 | 3.4251 |
| **17** | 4.2070 | 3.7480 | **61** | 5.3080 | 4.7170 | **105** | 5.6216 | 4.5792 |
| **18** | 5.5350 | 4.5200 | **62** | 4.9210 | 5.2080 | **106** | 6.1938 | 4.9115 |
| **19** | 6.4810 | 4.6550 | **63** | 4.6600 | 4.7120 | **107** | 5.1373 | 4.3686 |
| **20** | 4.7990 | 4.7020 | **64** | 4.9670 | 4.8070 | **108** | 5.5970 | 4.4610 |
| **21** | 6.1950 | 5.0260 | **65** | 5.9210 | 5.7960 | **109** | 6.5930 | 5.4350 |
| **22** | 5.8900 | 4.8770 | **66** | 4.7810 | 4.6830 | **110** | 6.7330 | 5.5720 |
| **23** | 3.7900 | 3.5230 | **67** | 5.6730 | 4.8630 | **111** | 6.7959 | 5.2518 |
| **24** | 5.0860 | 3.3690 | **68** | 4.4030 | 3.8970 | **112** | 5.9586 | 5.3279 |
| **25** | 4.6270 | 5.3480 | **69** | 4.3870 | 3.3980 | **113** | 6.2940 | 5.6460 |
| **26** | 5.1080 | 6.0230 | **70** | 7.1410 | 6.3060 | **114** | 4.7662 | 4.8831 |
| **27** | 4.9700 | 5.0500 | **71** | 7.1420 | 6.1450 | **115** | 6.2676 | 5.1612 |
| **28** | 3.4670 | 3.7280 | **72** | 6.6940 | 5.3660 | **116** | 4.8116 | 4.3088 |
| **29** | 5.5810 | 5.2230 | **73** | 7.8239 | 5.9393 | **117** | 5.2976 | 4.6619 |
| **30** | 5.7950 | 4.9920 | **74** | 7.1140 | 6.2830 | **118** | 5.3224 | 3.8941 |
| **31** | 4.7490 | 4.4790 | **75** | 6.5880 | 6.0570 | **119** | 5.3372 | 4.5498 |
| **32** | 5.9560 | 4.5920 | **76** | 6.7550 | 6.2170 | **120** | 6.3979 | 5.8239 |
| **33** | 5.8850 | 4.9910 | **77** | 6.7880 | 6.1930 | **121** | 6.3979 | 5.7447 |
| **34** | 6.4110 | 5.1400 | **78** | 6.6120 | 6.4850 | **122** | 5.8538 | 5.2757 |

| | | | | | | | | |
|---|---|---|---|---|---|---|---|---|
| **35** | 4.3100 | 3.8770 | **79** | 6.1780 | 5.8510 | **123** | 6.2146 | 5.2924 |
| **36** | 4.8450 | 4.8660 | **80** | 5.8500 | 5.9340 | **124** | 6.4437 | 5.2924 |
| **37** | 6.3210 | 5.1110 | **81** | 5.7040 | 5.1490 | **125** | 6.0555 | 5.1366 |
| **38** | 6.0260 | 4.8870 | **82** | 5.7930 | 5.2940 | **126** | 5.9208 | 5.2757 |
| **39** | 6.0950 | 4.9960 | **83** | 6.0840 | 5.9770 | **127** | 7.0969 | 5.2924 |
| **40** | 6.1560 | 5.1760 | **84** | 5.4960 | 5.5300 | **128** | 5.8860 | 5.2365 |
| **41** | 5.6930 | 4.8090 | **85** | 5.3770 | 4.8180 | **129** | 6.3767 | 5.7447 |
| **42** | 5.7030 | 5.1410 | **86** | 5.7670 | 4.8010 | **130** | 5.9208 | 5.2840 |
| **43** | 5.2050 | 4.9360 | **87** | 5.5528 | 4.7632 | | | |
| **44** | 4.5070 | 4.0530 | **88** | 5.5560 | 4.4016 | | | |

Table S3: STLs used as Test set in the QSAR models

| **Model 1** | **Model 2** | **Model 3-5** |
|---|---|---|
| 2 | 2 | 2 |
| 3 | 3 | 3 |
| 7 | 7 | 5 |
| 8 | 8 | 6 |
| 14 | 10 | 17 |
| 25 | 14 | 21 |
| 27 | 16 | 22 |
| 32 | 17 | 28 |
| 37 | 25 | 29 |
| 49 | 27 | 31 |
| 51 | 30 | 32 |
| 54 | 32 | 34 |
| 60 | 34 | 42 |
| 65 | 37 | 44 |
| 71 | 47 | 46 |
| 74 | 49 | 52 |
| 75 | 51 | 57 |
| 77 | 54 | 59 |
| 80 | 60 | 62 |
| 81 | 64 | 63 |
| 83 | 65 | 71 |
| 84 | 66 | 75 |
| 86 | 71 | 77 |
| 88 | 72 | 83 |
| 90 | 76 | 84 |
| 92 | 79 | 85 |
| 94 | 81 | 86 |

| | | |
|---|---|---|
| 100 | 85 | 92 |
| 103 | 88 | 93 |
| 105 | 91 | 101 |
| 106 | 101 | 103 |
| 107 | 103 | 105 |
| 109 | 105 | 107 |
| 111 | 106 | 111 |
| 115 | 108 | 113 |
| 117 | 116 | 115 |
| 118 | 119 | 116 |
| 119 | 123 | 117 |
| 121 | 126 | 121 |
| 123 | 127 | 123 |
| 125 | | 124 |
| 127 | | 125 |
| 129 | | 127 |
| | | 129 |

Table S4: GA/MLR equations of model 1

| Equation | RMSE | $R^2$ | $Q^2$ | adj$R^2$ | x_adj $R^2$ | AIC | LOF | F |
|---|---|---|---|---|---|---|---|---|
| 11.9036 + 0.0255 ASAP4 + 0.0270 ENONCS + -9.1834 FASA$^+$ + 2.150 npr1 + -1.669 vsurf_CW2 + 0.6144 vsurf_ID2 | 0.46 | 0.72 | 0.67 | 0.70 | 0.65 | 128.79 | 0.29 | 34.05 |
| 12.0792 + 0.0255 ASAP4 + 0.0272 ENONCS + -9.3959 FASA$^+$ + 2.1827 npr1 + -1.6946 vsurf_CW2 + 0.5937 vsurf_ID1 | 0.46 | 0.72 | 0.67 | 0.69 | 0.64 | 129.62 | 0.29 | 33.60 |
| 27.8764 + 0.0259 ASAP4 + 0.0248 ENONCS + -10.5589 FASA$^+$ + -0.7814 std_dim1 + -5.758 vsurf_CW1 + 0.6569 vsurf_ID2 | 0.46 | 0.72 | 0.67 | 0.69 | 0.64 | 129.64 | 0.29 | 33.59 |
| 31.6187 + 0.0262 ASAP4 + 0.0238 ENONCS + -12.7113 FASA$^+$ + -0.8864 rgyr + -6.3527 vsurf_CW1 + 0.6624 vsurf_ID2 | 0.47 | 0.71 | 0.67 | 0.69 | 0.64 | 130.08 | 0.29 | 33.35 |
| 17.7687 + 0.0265 ASAP4 + 0.0248 ENONCS + 10.6761 FASA$^-$ + -0.7937 std_dim1 + -5.9248 vsurf_CW1 + 0.6439 vsurf_ID2 | 0.47 | 0.71 | 0.67 | 0.69 | 0.64 | 130.36 | 0.29 | 33.20 |
| 2.7606 + 0.0261 ASAP4 + 0.0273 ENONCS + 9.0020 FASA$^-$ + 2.2131 npr1 + -1.6664 vsurf_CW2 + 0.6031 vsurf_ID2 | 0.47 | 0.71 | 0.66 | 0.69 | 0.64 | 130.81 | 0.29 | 32.96 |
| 13.0177 + 0.0254 ASAP4 + 0.0253 ENONCS + -9.8955 FASA$^+$ + 2.4895 glob + -1.8135 vsurf_CW2 + 0.6452 vsurf_ID2 | 0.47 | 0.71 | 0.66 | 0.69 | 0.63 | 131.90 | 0.30 | 32.38 |
| 17.9407 + 0.0263 ASAP4 + 0.0247 ENONCS + 10.8244 FASA$^-$ + -0.7663 std_dim1 + -6.0230 vsurf_CW1 + 0.5936 vsurf_ID1 | 0.47 | 0.71 | 0.66 | 0.68 | 0.63 | 132.50 | 0.30 | 32.07 |
| 19.2251 + 0.0268 ASAP4 + 0.0238 ENONCS + 12.6357 FASA- + -0.8739 rgyr + -6.4677 vsurf_CW1 + 0.6436 vsurf_ID2 | 0.47 | 0.71 | 0.66 | 0.68 | 0.64 | 132.65 | 0.30 | 31.99 |
| 7.0547 + 0.0299 ASAP4 + -0.0083 DASA + 0.0313 ENONCS + 2.6955 npr1 + -1.8094 vsurf_CW2 + 0.6920 vsurf_ID2 | 0.47 | 0.71 | 0.66 | 0.68 | 0.63 | 132.74 | 0.30 | 31.94 |
| 14.3828 + 0.0233 ASAP4 + -0.0099 DASA + 0.0262 ENONCS + 1.9684 npr1 + -3.8334 vsurf_CW1 + 0.6373 vsurf_ID2 | 0.48 | 0.70 | 0.65 | 0.68 | 0.62 | 133.61 | 0.30 | 31.49 |
| 11.9796 + 0.0250 ASAP4 + 0.0264 ENONCS + -9.2486 FASA$^+$ + 2.1631 npr1 + -1.6354 vsurf_CW2 + 0.4718 vsurf_ID3 | 0.48 | 0.70 | 0.65 | 0.68 | 0.63 | 133.90 | 0.31 | 31.35 |
| 3.1811 + 0.0260 ASAP4 + 0.0255 ENONCS + 9.7293 FASA- + 2.5566 glob + -1.8160 vsurf_CW2 + 0.6339 vsurf_ID2 | 0.48 | 0.70 | 0.65 | 0.68 | 0.63 | 134.22 | 0.31 | 31.18 |
| 39.8856 + -0.0203 ASA+ + 0.0307 ASAP4 + 0.0296 ENONCS + -11.5589 vsurf_CW1 + 0.0315 vsurf_HB5 + 0.5284 vsurf_ID2 | 0.48 | 0.70 | 0.65 | 0.68 | 0.63 | 134.26 | 0.31 | 31.16 |

| | | | | | | | | |
|---|---|---|---|---|---|---|---|---|
| 12.65 + 0.0264 ASAP4 + 0.0263 ENONCS + -9.4162 FASA+ + 2.0175 npr1 + -1.8488 vsurf_CW2 + 0.2970 vsurf_ID6 | 0.48 | 0.70 | 0.64 | 0.67 | 0.62 | 135.74 | 0.31 | 30.41 |
| 12.1653 + 0.0253 ASAP4 + 0.0260 ENONCS + -9.2558 FASA+ + 2.1489 npr1 + -1.7002 vsurf_CW2 + 0.3749 vsurf_ID4 | 0.48 | 0.70 | 0.65 | 0.67 | 0.62 | 135.79 | 0.31 | 30.39 |
| 2.7887 + 0.0257 ASAP4 + 0.0267 ENONCS + 9.0252 FASA- + 2.2315 npr1 + -1.6324 vsurf_CW2 + 0.4546 vsurf_ID3 | 0.48 | 0.69 | 0.65 | 0.67 | 0.62 | 135.98 | 0.31 | 30.29 |
| 12.2963 + 0.0258 ASAP4 + 0.0259 ENONCS + -9.1341 FASA+ + 2.1039 npr1 + -1.7873 vsurf_CW2 + 0.3199 vsurf_ID5 | 0.48 | 0.69 | 0.64 | 0.67 | 0.62 | 136.17 | 0.31 | 30.20 |
| 41.2157 + 0.0263 ASAP4 + 0.0234 ENONCS + -13.3411 FASA+ + -6.996 vsurf_CW1 + -8.3835 vsurf_G + 0.6403 vsurf_ID2 | 0.48 | 0.69 | 0.64 | 0.67 | 0.62 | 136.18 | 0.31 | 30.19 |
| 7.3986 + 0.0239 ASAP4 + 0.0235 ENONCS + -11.3888 FASA+ + 3.5354 FASA_H + 2.3993 npr1 + 0.6330 vsurf_ID2 | 0.48 | 0.69 | 0.64 | 0.67 | 0.62 | 136.33 | 0.31 | 30.11 |
| 10.934 + 0.0239 ASAP4 + 0.0235 ENONCS + -11.3888 FASA+ + -3.5354 FASA_P + 2.3993 npr1 + 0.6330 vsurf_ID2 | 0.48 | 0.69 | 0.64 | 0.67 | 0.62 | 136.33 | 0.31 | 30.11 |
| 13.0605 + 0.0248 ASAP4 + 0.0247 ENONCS + -9.9478 FASA+ + 2.5311 glob + -1.7763 vsurf_CW2 + 0.5133 vsurf_ID3 | 0.48 | 0.69 | 0.64 | 0.67 | 0.61 | 136.49 | 0.31 | 30.03 |
| 28.7838 + 0.0272 ASAP4 + 0.0233 ENONCS + 13.6265 FASA- + -7.2601 vsurf_CW1 + -8.6186 vsurf_G + 0.6243 vsurf_ID2 | 0.48 | 0.69 | 0.64 | 0.67 | 0.61 | 136.77 | 0.32 | 29.90 |
| 12.0562 + 0.0275 ASAP4 + 0.0257 ENONCS + -10.4784 FASA+ + 2.4097 npr1 + 0.1224 vsurf_A + -1.3174 vsurf_CW2 | 0.49 | 0.69 | 0.64 | 0.67 | 0.61 | 137.24 | 0.32 | 29.66 |
| 18.3066 + 0.0271 ASAP4 + 0.0210 ENONCS + -13.9749 FASA+ + -0.4016 std_dim1 + -2.3029 vsurf_CW2 + 0.7284 vsurf_ID2 | 0.49 | 0.69 | 0.64 | 0.67 | 0.61 | 137.30 | 0.32 | 29.63 |
| 40.0793 + -0.0205 ASA+ + 0.0302 ASAP4 + 0.0291 ENONCS + -11.5867 vsurf_CW1 + 0.0324 vsurf_HB5 + 0.4153 vsurf_ID3 | 0.49 | 0.69 | 0.64 | 0.67 | 0.61 | 137.36 | 0.32 | 29.61 |
| 9.4266 + 0.0250 ASAP4 + 0.0265 ENONCS + -10.0586 FASA+ + 3.1714 npr1 + -1.4912 vsurf_CW4 + 0.6932 vsurf_ID2 | 0.49 | 0.69 | 0.64 | 0.67 | 0.61 | 137.49 | 0.32 | 29.54 |
| 12.3535 + 0.0271 ASAP4 + 0.02729 ENONCS + -8.8024 FASA+ + 2.3172 npr1 + -1.7337 vsurf_CW2 + -0.0503 vsurf_DD12 | 0.49 | 0.69 | 0.64 | 0.66 | 0.61 | 137.83 | 0.32 | 29.37 |
| 3.2076 + 0.0265 ASAP4 + 0.0262 ENONCS + 8.9032 FASA- + 2.1764 npr1 + -1.7769 vsurf_CW2 + 0.3051 vsurf_ID5 | 0.49 | 0.69 | 0.64 | 0.66 | 0.61 | 138.20 | 0.32 | 29.19 |
| 13.2815 + 0.0252 ASAP4 + 0.0242 ENONCS + -9.9792 FASA+ + 2.4914 glob + -1.8477 vsurf_CW2 + 0.4095 vsurf_ID4 | 0.49 | 0.69 | 0.63 | 0.66 | 0.61 | 138.56 | 0.32 | 29.01 |

| | | | | | | | | |
|---|---|---|---|---|---|---|---|---|
| 11.3189 + 0.0236 ASAP4 + 0.0227 ENONCS + -11.7846 FASA+ + -3.7680* FASA_P + 2.3791 npr1 + 0.5441 vsurf_ID3 | 0.49 | 0.68 | 0.63 | 0.66 | 0.61 | 138.79 | 0.32 | 28.90 |
| 12.5805 + 0.0260 ASAP4 + 0.0258 ENONCS + -9.3743 FASA+ + 1.8744 npr1 + -1.7193 vsurf_CW2 + 0.1785 vsurf_ID8 | 0.49 | 0.68 | 0.63 | 0.66 | 0.60 | 139.03 | 0.32 | 28.79 |
| 40.1871 + -0.0200 ASA+ + 0.0303 ASAP4 + 0.0285 ENONCS + -11.649 vsurf_CW1 + 0.0315 vsurf_HB5 + 0.2707 vsurf_ID5 | 0.49 | 0.68 | 0.63 | 0.66 | 0.61 | 139.36 | 0.33 | 28.63 |
| 26.4442 + -0.0247 ASA+ + 0.0265 ASAP4 + 0.0245 ENONCS + -7.1306 vsurf_CW1 + 0.7186 vsurf_ID2 + 0.0061 vsurf_V | 0.49 | 0.68 | 0.61 | 0.66 | 0.58 | 139.36 | 0.33 | 28.63 |
| 31.3854 + 0.0261 ASAP4 + 0.0231 ENONCS + -12.0691 FASA+ + -0.7786* rgyr + -6.4577 vsurf_CW1 + 0.2821 vsurf_ID6 | 0.49 | 0.68 | 0.63 | 0.66 | 0.60 | 139.64 | 0.33 | 28.49 |
| 35.6512 + -0.0051 ASA + 0.0272 ASAP4 + 0.0203 ENONCS + -13.7209 FASA+ + -7.6750 vsurf_CW1 + 0.6231 vsurf_ID2 | 0.49 | 0.68 | 0.63 | 0.66 | 0.60 | 139.76 | 0.33 | 28.44 |
| 39.4199 + -0.0158 ASA+ + 0.0314 ASAP4 + 0.0307 ENONCS + -11.9394 vsurf_CW1 + 4.9469 vsurf_CW5 + 0.5025 vsurf_ID2 | 0.49 | 0.68 | 0.63 | 0.66 | 0.60 | 140.07 | 0.33 | 28.28 |
| 11.1832 + 0.0081 ASAN3 + 0.0270 ASAP4 + 0.0228 ENONCS + -8.0275 FASA+ + 2.3996 npr1 + -1.5247 vsurf_CW2 | 0.49 | 0.68 | 0.63 | 0.66 | 0.60 | 140.13 | 0.33 | 28.26 |
| 12.0543 + 0.0279 ASAP4 + 0.0258 ENONCS + -10.2572 FASA+ + 2.4103 npr1 + -1.3305 vsurf_CW2 + 0.1189 vsurf_IW5 | 0.49 | 0.68 | 0.62 | 0.65 | 0.60 | 140.23 | 0.33 | 28.21 |
| 6.79621 + 0.0227 ASAP4 + 0.0295 ENONCS + -6.8726 FASA+ + 2.8084 npr1 + 0.6837 vsurf_ID2 + -0.0449 vsurf_Wp4 | 0.49 | 0.68 | 0.63 | 0.65 | 0.60 | 140.33 | 0.33 | 28.16 |
| 12.1497 + 0.0272 ASAP4 + 0.0257 ENONCS + -10.2139 FASA+ + 2.3902* npr1 + -1.3615 vsurf_CW2 + 0.1092 vsurf_IW4 | 0.49 | 0.68 | 0.62 | 0.65 | 0.60 | 140.33 | 0.33 | 28.16 |
| 3.2112 + 0.0265 ASAP4 + 0.0259 ENONCS + 9.3083 FASA- + 1.9264 npr1 + -1.7205 vsurf_CW2 + 0.1775 vsurf_ID8 | 0.49 | 0.68 | 0.62 | 0.65 | 0.59 | 140.37 | 0.33 | 28.14 |
| 3.1070 + 0.0089 ASAN3 + 0.0274 ASAP4 + 0.0223 ENONCS + 8.0600 FASA- + 2.4387 npr1 + -1.5200 vsurf_CW2 | 0.49 | 0.68 | 0.63 | 0.65 | 0.60 | 140.49 | 0.33 | 28.09 |
| 32.9205 + 0.0277 ASAP4 + 0.0212 ENONCS + -12.9948 FASA+ + -6.9665 vsurf_CW1 + 0.6286 vsurf_ID2 + -0.0029 vsurf_Wp1 | 0.50 | 0.68 | 0.63 | 0.65 | 0.60 | 140.60 | 0.33 | 28.03 |
| 8.3474 + 0.0221 ASAP4 + 0.0257 ENONCS + -8.9461 FASA+ + 3.0156 npr1 + -7.7290 vsurf_HL2 + 0.6945 vsurf_ID2 | 0.50 | 0.68 | 0.63 | 0.65 | 0.60 | 140.66 | 0.33 | 28.00 |
| 32.5678 + -0.0081 ASA+ + 0.0266 ASAP4 + 0.0206 ENONCS + -9.5172 FASA+ + -7.5155 vsurf_CW1 + 0.6459 vsurf_ID2 | 0.50 | 0.68 | 0.63 | 0.65 | 0.60 | 140.67 | 0.33 | 28.00 |

| | | | | | | | | |
|---|---|---|---|---|---|---|---|---|
| 12.2136 + 0.0268 ASAP4 + 0.0258 ENONCS + -10.0868 FASA+ + 2.3925 npr1 + -1.4158 vsurf_CW2 + 0.1076 vsurf_IW3 | 0.50 | 0.68 | 0.62 | 0.65 | 0.59 | 140.75 | 0.33 | 27.96 |
| 18.3791 + 0.0267 ASAP4 + 0.0207 ENONCS + -14.158 FASA+ + -0.3593 std_dim1 + -2.3057 vsurf_CW2 + 0.6536 vsurf_ID1 | 0.50 | 0.68 | 0.63 | 0.65 | 0.60 | 140.77 | 0.33 | 27.95 |
| 17.3908 + 0.0253 ASAP4 + 0.0218 ENONCS + -13.5262 FASA+ + -2.2498* vsurf_CW2 + -0.0130 vsurf_D8 + 0.6365 vsurf_ID2 | 0.50 | 0.68 | 0.62 | 0.65 | 0.59 | 140.86 | 0.33 | 27.91 |
| 8.7841 + 0.0238 ASAP4 + 0.0260 ENONCS + -9.2646 FASA+ + 3.0940 npr1 + -2.4822 vsurf_CW5 + 0.6866 vsurf_ID2 | 0.50 | 0.68 | 0.63 | 0.65 | 0.60 | 141.10 | 0.33 | 27.80 |
| 30.4707 + 0.0262 ASAP4 + 0.0231 ENONCS + -12.3005 FASA+ + -0.8212 rgyr + -5.9704 vsurf_CW1 + 0.1920 vsurf_ID8 | 0.50 | 0.68 | 0.61 | 0.65 | 0.59 | 141.13 | 0.33 | 27.78 |
| 11.7309 + 0.02647 ASAP4 + 0.0264 ENONCS + -8.2401 FASA+ + 2.2773 npr1 + -1.6530 vsurf_CW2 + 0.0615 vsurf_DW12 | 0.50 | 0.68 | 0.62 | 0.65 | 0.60 | 141.15 | 0.33 | 27.77 |
| 9.8740 + 0.0234 ASAP4 + 0.0258 ENONCS + -10.5716 FASA+ + 2.6705 npr1 + -0.0024 vsurf_HB1 + 0.6622 vsurf_ID2 | 0.50 | 0.67 | 0.62 | 0.65 | 0.60 | 141.54 | 0.33 | 27.59 |
| 13.3166 + 0.0282 ASAP4 + -0.0022 ASA_P + 0.0257 ENONCS + -10.4294 FASA+ + 2.3018 npr1 + -1.5605 vsurf_CW2 | 0.50 | 0.67 | 0.62 | 0.65 | 0.59 | 141.55 | 0.33 | 27.59 |
| 44.3663 + -0.0102 ASA + 0.0290 ASAP4 + 0.0242 ENONCS + -12.3609 FASA+ + -10.4551 vsurf_CW1 + 0.0244 vsurf_HB5 | 0.50 | 0.67 | 0.62 | 0.65 | 0.59 | 141.56 | 0.33 | 27.58 |
| 27.2116 + -0.0123 ASA+ + 0.0308 ASAP4 + 0.0300 ENONCS + 7.5611 dens + -10.2629 vsurf_CW1 + 0.7012 vsurf_ID2 | 0.50 | 0.67 | 0.63 | 0.65 | 0.60 | 141.59 | 0.33 | 27.56 |
| 31.5231 + -0.0104 ASA- + 0.0285 ASAP4 + 0.0255 ENONCS + -15.2992 FASA+ + 1.755 npr1 + -6.1274 vsurf_CW1 | 0.50 | 0.67 | 0.62 | 0.65 | 0.59 | 141.63 | 0.33 | 27.54 |
| 12.0586 + 0.0288 ASAP4 + 0.0261 ENONCS + -10.0287 FASA+ + 2.4420* npr1 + -1.4265 vsurf_CW2 + 0.1033 vsurf_IW6 | 0.50 | 0.67 | 0.62 | 0.65 | 0.59 | 141.65 | 0.33 | 27.54 |
| 26.4197 + 0.0284 ASAP4 + 0.0252 ENONCS + -12.5807 FASA+ + -0.9286* FCASA- + 2.2860 npr1 + -5.2595 vsurf_CW1 | 0.50 | 0.67 | 0.62 | 0.65 | 0.59 | 141.65 | 0.33 | 27.54 |
| 16.9053 + 0.0253 ASAP4 + 0.0214 ENONCS + -12.3058 FASA+ + -2.3004* vsurf_CW2 + -0.0079 vsurf_D7 + 0.6468 vsurf_ID2 | 0.50 | 0.67 | 0.61 | 0.65 | 0.59 | 141.67 | 0.33 | 27.53 |
| 12.4116 + 0.0267 ASAP4 + 0.0259 ENONCS + -9.4830 FASA+ + 2.1491 npr1 + -1.6487 vsurf_CW2 + 0.4390 vsurf_IW1 | 0.50 | 0.67 | 0.62 | 0.65 | 0.59 | 141.70 | 0.33 | 27.52 |
| 13.259 + 0.0305 ASAP4 + 0.0319 ENONCS + -0.0025 ENONS + -9.9401 FASA+ + 2.1316 npr1 + -1.7831 vsurf_CW2 | 0.50 | 0.67 | 0.62 | 0.65 | 0.59 | 141.72 | 0.33 | 27.50 |

| | | | | | | | | |
|---|---|---|---|---|---|---|---|---|
| 23.5349 + -0.0082 ASA+ + 0.0273 ASAP4 + 0.0206 ENONCS + 9.4980 FASA- + -7.6793 vsurf_CW1 + 0.6334 vsurf_ID2 | 0.50 | 0.67 | 0.62 | 0.65 | 0.59 | 141.82 | 0.33 | 27.46 |
| 19.1585 + 0.0305 ASAP4 + -0.0017 DCASA + 0.0312 ENONCS + 2.9188 glob + -5.8225 vsurf_CW1 + 0.6523 vsurf_ID2 | 0.50 | 0.67 | 0.62 | 0.65 | 0.59 | 141.83 | 0.33 | 27.45 |
| 28.1035 + 0.0274 ASAP4 + 0.0223 ENONCS + -11.9733 FASA+ + -0.6757 std_dim1 + 0.0996 vsurf_A + -5.5009 vsurf_CW1 | 0.50 | 0.67 | 0.62 | 0.65 | 0.59 | 141.86 | 0.33 | 27.44 |
| 0.0069 + 0.0233 ASAP4 + 0.0299 ENONCS + 6.5661 FASA- + 2.8738 npr1 + 0.6754 vsurf_ID2 + -0.0453 vsurf_Wp4 | 0.50 | 0.67 | 0.62 | 0.65 | 0.59 | 141.88 | 0.33 | 27.43 |
| 26.988 + 0.0259 ASAP4 + 0.0238 ENONCS + -10.2918 FASA+ + -0.7095 std_dim1 + -5.4193 vsurf_CW1 + 0.1682 vsurf_ID8 | 0.50 | 0.67 | 0.61 | 0.65 | 0.58 | 141.92 | 0.33 | 27.41 |
| 16.3853 + 0.0207 ASAP4 + 0.0189 ENONCS + -12.6755 FASA+ + -0.5040 dipole + -2.1027 vsurf_CW2 + 0.8969 vsurf_ID2 | 0.50 | 0.67 | 0.62 | 0.65 | 0.59 | 142.03 | 0.34 | 27.36 |
| 17.0927 + 0.0264 ASAP4 + 0.0237 ENONCS + 10.4901 FASA- + -0.7229 std_dim1 + -5.5787 vsurf_CW1 + 0.1665 vsurf_ID8 | 0.50 | 0.67 | 0.61 | 0.65 | 0.58 | 142.03 | 0.34 | 27.36 |
| 15.9558 + 0.0235 ASAP4 + 0.01917 ENONCS + -12.8864 FASA+ + -2.0338 vsurf_CW2 + 0.5289 vsurf_ID2 + 0.1992 vsurf_ID8 | 0.50 | 0.67 | 0.61 | 0.65 | 0.58 | 142.04 | 0.34 | 27.36 |
| 13.6659 + 0.0269 ASAP4 + 0.0251 ENONCS + -9.645 FASA+ + 2.6147 glob + -1.8948 vsurf_CW2 + -0.0505 vsurf_DD12 | 0.50 | 0.67 | 0.62 | 0.65 | 0.59 | 142.08 | 0.34 | 27.33 |
| 9.5374 + 0.0243 ASAP4 + 0.0257 ENONCS + -10.1073 FASA+ + 3.1506 npr1 + -1.4383 vsurf_CW4 + 0.5550 vsurf_ID3 | 0.50 | 0.67 | 0.62 | 0.65 | 0.60 | 142.12 | 0.34 | 27.32 |
| 18.4127 + 0.0264 ASAP4 + 0.0202 ENONCS + -14.1029 FASA+ + -0.4026 std_dim1 + -2.2650 vsurf_CW2 + 0.5914 vsurf_ID3 | 0.50 | 0.67 | 0.62 | 0.65 | 0.59 | 142.17 | 0.34 | 27.29 |
| 22.0811 + -0.0049 ASA + 0.0277 ASAP4 + 0.0201 ENONCS + 13.6227 FASA- + -7.7326 vsurf_CW1 + 0.6031 vsurf_ID2 | 0.50 | 0.67 | 0.62 | 0.65 | 0.59 | 142.20 | 0.34 | 27.28 |
| 6.9714 + 0.0218 ASAP4 + 0.0284 ENONCS + -7.0455 FASA+ + 2.7393 npr1 + 0.6525 vsurf_ID2 + -0.128 vsurf_Wp5 | 0.50 | 0.67 | 0.62 | 0.65 | 0.59 | 142.20 | 0.34 | 27.28 |
| 21.5241 + -0.0072 ASA+ + 0.0335 ASAP4 + 0.0356 ENONCS + 2.5005 npr1 + -6.289 vsurf_CW1 + 0.5935 vsurf_ID2 | 0.50 | 0.67 | 0.63 | 0.65 | 0.60 | 142.23 | 0.34 | 27.26 |
| 19.122 + 0.0264 ASAP4 + 0.0205 ENONCS + -14.1019 FASA+ + -2.6207 vsurf_CW2 + -0.0018 vsurf_D1 + 0.6840 vsurf_ID2 | 0.50 | 0.67 | 0.61 | 0.65 | 0.59 | 142.25 | 0.34 | 27.25 |
| 44.5294 + -0.0101 ASA + 0.0289 ASAP4 + 0.0237 ENONCS + -12.6245 FASA+ + -10.4557 vsurf_CW1 + 0.0230 vsurf_W5 | 0.50 | 0.67 | 0.61 | 0.65 | 0.59 | 142.30 | 0.34 | 27.23 |

| | | | | | | | | |
|---|---|---|---|---|---|---|---|---|
| 9.37255 + 0.0231 ASAP4 + 0.0257 ENONCS + -10.1759 FASA+ + 2.8337 npr1 + -0.0015 vsurf_HB2 + 0.6455 vsurf_ID2 | 0.50 | 0.67 | 0.62 | 0.65 | 0.59 | 142.30 | 0.34 | 27.23 |
| 12.5791 + 0.0209 ASAP4 + 0.0260 ENONCS + -7.4100 FASA+ + 2.1557 npr1 + -1.8989 vsurf_CW1 + 0.5645 vsurf_ID2 | 0.50 | 0.67 | 0.61 | 0.65 | 0.58 | 142.33 | 0.34 | 27.22 |
| 7.32746 + 0.01967 ASAP4 + 0.0267 ENONCS + -8.1221 FASA+ + 0.1918 logP(o/w) + 2.6672 npr1 + 0.6496 vsurf_ID2 | 0.50 | 0.67 | 0.62 | 0.65 | 0.59 | 142.35 | 0.34 | 27.21 |
| 35.6228 + -0.0172* ASA+ + 0.0304 ASAP4 + 0.0303 ENONCS + 1.1596 glob + -10.2078 vsurf_CW1 + 0.0274 vsurf_HB5 | 0.50 | 0.67 | 0.62 | 0.65 | 0.59 | 142.36 | 0.34 | 27.21 |
| 25.7998 + 0.0291 ASAP4 + 0.0271 ENONCS + -9.4251 FASA+ + 1.8992 npr1 + -5.4808 vsurf_CW1 + -0.0023 vsurf_Wp1 | 0.50 | 0.67 | 0.62 | 0.65 | 0.59 | 142.38 | 0.34 | 27.20 |
| 11.3123 + 0.0271 ASAP4 + 0.0253 ENONCS + -10.0816 FASA+ + 1.5040 FASA_H + 2.2519 npr1 + -1.3550 vsurf_CW2 | 0.50 | 0.67 | 0.61 | 0.65 | 0.58 | 142.39 | 0.34 | 27.19 |
| 12.8163 + 0.0271 ASAP4 + 0.0253 ENONCS + -10.0816 FASA+ + -1.504 FASA_P + 2.2519 npr1 + -1.3550 vsurf_CW2 | 0.50 | 0.67 | 0.61 | 0.65 | 0.58 | 142.39 | 0.34 | 27.19 |
| 13.5957 + 0.0225 ASAP4 + 0.0252 ENONCS + -10.2473 FASA+ + -4.6064 dens + 3.1092 npr1 + 0.565 vsurf_ID2 | 0.50 | 0.67 | 0.62 | 0.65 | 0.59 | 142.40 | 0.34 | 27.19 |
| 19.8244 + 0.0273 ASAP4 + -0.0052 ASA_P + 0.02378 ENONCS + -10.9202 FASA+ + 1.9829 npr1 + -3.25263 vsurf_CW1 | 0.50 | 0.67 | 0.61 | 0.65 | 0.58 | 142.42 | 0.34 | 27.18 |
| 14.0332 + 0.02783 ASAP4 + 0.0268 ENONCS + -9.7209 FASA+ + 2.1422 npr1 + -2.0197 vsurf_CW2 + -0.0018 vsurf_D3 | 0.50 | 0.67 | 0.61 | 0.65 | 0.58 | 142.43 | 0.34 | 27.17 |
| 9.6766 + 0.0231 ASAP4 + -0.0034 ASA_P + 0.0246 ENONCS + -10.3999 FASA+ + 2.6266 npr1 + 0.6351 vsurf_ID2 | 0.50 | 0.67 | 0.62 | 0.65 | 0.59 | 142.43 | 0.34 | 27.17 |
| 13.1954 + 0.0270 ASAP4 + 0.02721 ENONCS + -9.4842 FASA+ + 2.0004 npr1 + -1.7952 vsurf_CW2 + -0.0063 vsurf_D8 | 0.50 | 0.67 | 0.61 | 0.65 | 0.58 | 142.48 | 0.34 | 27.15 |
| 13.165 + 0.0281 ASAP4 + -0.0002 CASA- + 0.0264 ENONCS + -9.9210 FASA+ + 2.3416 npr1 + -1.7589 vsurf_CW2 | 0.50 | 0.67 | 0.61 | 0.65 | 0.58 | 142.50 | 0.34 | 27.14 |
| 25.4617 + 0.0251 ASAP4 + -0.0062 ASA_P + 0.0167 ENONCS + -14.7965 FASA+ + -4.1913 vsurf_CW1 + 0.6166 vsurf_ID2 | 0.50 | 0.67 | 0.61 | 0.65 | 0.58 | 142.54 | 0.34 | 27.12 |
| 12.0128 + -0.0029 ASAN7 + 0.02747 ASAP4 + 0.0276 ENONCS + -9.0488 FASA+ + 2.4874 npr1 + -1.5441 vsurf_CW2 | 0.50 | 0.67 | 0.61 | 0.65 | 0.58 | 142.54 | 0.34 | 27.12 |
| 39.4846 + -0.0197 ASA+ + 0.0323 ASAP4 + -0.0121 ASAP6 + 0.0274 ENONCS + -11.2883 vsurf_CW1 + 0.0335 vsurf_HB5 | 0.50 | 0.67 | 0.62 | 0.65 | 0.59 | 142.57 | 0.34 | 27.11 |

| | | | | | | | | |
|---|---|---|---|---|---|---|---|---|
| 8.43708 + 0.0220 ASAP4 + 0.0255 ENONCS + -8.8848 FASA+ + 3.0004 npr1 + -5.6255 vsurf_CW6 + 0.6903 vsurf_ID2 | 0.50 | 0.67 | 0.62 | 0.65 | 0.59 | 142.59 | 0.34 | 27.10 |
| 6.8163 + 0.0203 ASAP4 + 0.0256 ENONCS + -8.02079 FASA+ + 2.8207 npr1 + 0.0047 vsurf_D6 + 0.5825 vsurf_ID2 | 0.50 | 0.67 | 0.61 | 0.65 | 0.58 | 142.60 | 0.34 | 27.09 |
| 12.1905 + 0.0268 ASAP4 + 0.0268 ENONCS + -8.6924 FASA+ + 2.2033npr1 + -1.7254 vsurf_CW2 + 0.0325 vsurf_DW13 | 0.50 | 0.67 | 0.62 | 0.65 | 0.59 | 142.61 | 0.34 | 27.09 |
| 27.9143 + -0.0039 ASA + 0.0284 ASAP4 + 0.0259 ENONCS + -10.0718 FASA+ + 1.8164 npr1 + -6.0067 vsurf_CW1 | 0.50 | 0.67 | 0.62 | 0.65 | 0.59 | 142.62 | 0.34 | 27.09 |
| 13.81 + 0.0277 ASAP4 + 0.0270 ENONCS + -9.5378 FASA+ + 2.1238 npr1 + -1.9563 vsurf_CW2 + -0.0008 vsurf_D1 | 0.50 | 0.67 | 0.61 | 0.65 | 0.58 | 142.62 | 0.34 | 27.08 |
| 18.4438 + 0.0268 ASAP4 + 0.0229 ENONCS + 12.332 FASA- + -0.8126 rgyr + -6.08402 vsurf_CW1 + 0.1903 vsurf_ID8 | 0.50 | 0.67 | 0.61 | 0.65 | 0.58 | 142.63 | 0.34 | 27.08 |

Table S5 GA/MLR equations for model 2

| Equation | RMSE | $R^2$ | $Q^2$ | adj$R^2$ | x_adj$R^2$ | AIC | LOF | F |
|---|---|---|---|---|---|---|---|---|
| -1.4803 + 0.0169 ASAP4 + 0.0134 ENONCS + 12.3791 FASA- + -2.1774 vsurf_CW2 + -1.1349 vsurf_EWmin3 + -1.5860 vsurf_HB8 | 0.47 | 0.71 | 0.67 | 0.68 | 0.64 | 133.98 | 0.29 | 33.17 |
| -1.4801 + 0.0169 ASAP4 + 0.0134 ENONCS + 12.3791 FASA- + -2.1773 vsurf_CW2 + -1.1348 vsurf_EWmin3 + -1.5859 vsurf_W8 | 0.47 | 0.71 | 0.67 | 0.68 | 0.64 | 133.99 | 0.29 | 33.17 |
| 3.3263 + 0.0171 ASAP4 + -0.0226 ASAP6 + 0.0109 ENONCS + 13.9402 FASA- + -2.0114 vsurf_CW2 + 0.5432 vsurf_ID2 | 0.47 | 0.70 | 0.66 | 0.68 | 0.64 | 134.31 | 0.29 | 33.00 |
| 3.83209 + 0.0167 ASAP4 + -0.0178 ASAP6 + 0.0127 ENONCS + 13.4613 FASA- + -2.1945 vsurf_CW2 + 0.3399 vsurf_ID7 | 0.47 | 0.70 | 0.66 | 0.68 | 0.63 | 134.70 | 0.29 | 32.80 |
| 10.9 + 0.0171 ASAP4 + 0.0136 ENONCS + -12.2378 FASA+ + -2.2035 vsurf_CW2 + -1.1268 vsurf_EWmin3 + -1.5748 vsurf_HB8 | 0.47 | 0.70 | 0.66 | 0.68 | 0.64 | 135.20 | 0.29 | 32.54 |
| 17.2454 + 0.0172 ASAP4 + -0.0224 ASAP6 + 0.0110 ENONCS + -13.813 FASA+ + -2.0431 vsurf_CW2 + 0.5418 vsurf_ID2 | 0.47 | 0.70 | 0.66 | 0.68 | 0.63 | 135.46 | 0.29 | 32.41 |

| | | | | | | | | |
|---|---|---|---|---|---|---|---|---|
| 3.9667 + 0.0153 ASAP4 + 0.0155 ENONCS + 12.5953 FASA- + -2.1771 vsurf_CW2 + -0.2106 vsurf_HB8 + 0.3704 vsurf_ID7 | 0.47 | 0.70 | 0.65 | 0.68 | 0.63 | 135.63 | 0.29 | 32.31 |
| 3.9667 + 0.0153 ASAP4 + 0.0155 ENONCS + 12.5954 FASA- + -2.1771 vsurf_CW2 + 0.3704 vsurf_ID7 + -0.2106 vsurf_W8 | 0.47 | 0.70 | 0.65 | 0.68 | 0.63 | 135.63 | 0.29 | 32.31 |
| 17.264 + 0.0169 ASAP4 + -0.0175 ASAP6 + 0.0128ENONCS + -13.3265 FASA+ + -2.2242 vsurf_CW2 + 0.3385 vsurf_ID7 | 0.47 | 0.70 | 0.65 | 0.68 | 0.63 | 135.86 | 0.30 | 32.20 |
| 3.52157 + 0.0167 ASAP4 + -0.0197 ASAP6 + 0.0109 ENONCS + 14.0233 FASA- + -2.1072 vsurf_CW2 + 0.3390 vsurf_ID6 | 0.47 | 0.70 | 0.65 | 0.68 | 0.63 | 135.87 | 0.30 | 32.19 |
| 3.9378 + 0.0154 ASAP4 + 0.0155 ENONCS + 12.5714 FASA- + -2.1568 vsurf_CW2 + -32.5012 vsurf_CW8 + 0.3651 vsurf_ID7 | 0.47 | 0.70 | 0.65 | 0.68 | 0.62 | 135.99 | 0.30 | 32.13 |
| 3.7930 + 0.0156 ASAP4 + 0.0146 ENONCS + 12.8574 FASA- + -2.0748 vsurf_CW2 + -0.2859 vsurf_HB8 + 0.2903 vsurf_ID8 | 0.47 | 0.70 | 0.65 | 0.68 | 0.62 | 136.17 | 0.30 | 32.04 |
| 3.7930 + 0.0156 ASAP4 + 0.0146 ENONCS + 12.8575 FASA- + -2.0747 vsurf_CW2 + 0.2903 vsurf_ID8 + -0.2859 vsurf_W8 | 0.47 | 0.70 | 0.65 | 0.68 | 0.62 | 136.17 | 0.30 | 32.04 |
| 3.3509 + 0.0170 ASAP4 + -0.0216 ASAP6 + 0.0111 ENONCS + 14.0486 FASA- + -2.0297 vsurf_CW2 + 0.4910 vsurf_ID1 | 0.47 | 0.70 | 0.65 | 0.68 | 0.63 | 136.32 | 0.30 | 31.97 |
| 2.5156 + -0.2402 AM1_HOMO + 0.0158 ASAP4 + 0.0157 ENONCS + 11.7798 FASA- + -2.5307 vsurf_CW2 + 0.3956 vsurf_ID7 | 0.47 | 0.70 | 0.65 | 0.68 | 0.62 | 136.41 | 0.30 | 31.92 |
| 3.7777 + 0.0146 ASAP4 + 0.0157 ENONCS + 12.9922 FASA- + -2.1572 vsurf_CW2 + -0.0797 vsurf_HB7 + 0.4339 vsurf_ID7 | 0.47 | 0.70 | 0.65 | 0.68 | 0.62 | 136.49 | 0.30 | 31.88 |
| 3.7551 + 0.0157 ASAP4 + 0.0147 ENONCS + 12.8164 FASA- + -2.0466 vsurf_CW2 + -44.4324 vsurf_CW8 + 0.2833 vsurf_ID8 | 0.47 | 0.70 | 0.64 | 0.68 | 0.62 | 136.61 | 0.30 | 31.82 |
| 3.2289 + 0.0173 ASAP4 + -0.0230 ASAP6 + 0.0106 ENONCS + 14.0164 FASA- + -1.9501 vsurf_CW2 + 0.4477 vsurf_ID3 | 0.47 | 0.70 | 0.65 | 0.67 | 0.63 | 136.70 | 0.30 | 31.77 |
| 16.535 + 0.0155 ASAP4 + 0.0156 ENONCS + -12.4686 FASA+ + -2.2046 vsurf_CW2 + -0.2091 vsurf_HB8 + 0.3688 vsurf_ID7 | 0.47 | 0.70 | 0.65 | 0.67 | 0.62 | 136.72 | 0.30 | 31.76 |
| 16.535 + 0.0155 ASAP4 + 0.0156 ENONCS + -12.4686 FASA+ + -2.2046 vsurf_CW2 + 0.3688 vsurf_ID7 + -0.2091 vsurf_W8 | 0.47 | 0.70 | 0.65 | 0.67 | 0.62 | 136.72 | 0.30 | 31.76 |

| | | | | | | | | |
|---|---|---|---|---|---|---|---|---|
| 17.5464 + 0.0168 ASAP4 + -0.0194 ASAP6 + 0.0110 ENONCS + -13.932 FASA+ + -2.1413 vsurf_CW2 + 0.3428 vsurf_ID6 | 0.47 | 0.70 | 0.65 | 0.67 | 0.63 | 136.73 | 0.30 | 31.76 |
| 16.4816 + 0.0156 ASAP4 + 0.0156 ENONCS + -12.4426 FASA+ + -2.1847 vsurf_CW2 + -32.1719 vsurf_CW8 + 0.3635 vsurf_ID7 | 0.47 | 0.70 | 0.65 | 0.67 | 0.62 | 137.09 | 0.30 | 31.58 |
| 14.2416 + -0.2426 AM1_HOMO + 0.0160 ASAP4 + 0.0158 ENONCS + -11.6489 FASA+ + -2.5584 vsurf_CW2 + 0.3943 vsurf_ID7 | 0.47 | 0.69 | 0.65 | 0.67 | 0.62 | 137.36 | 0.30 | 31.44 |
| 3.46518 + 0.0175 ASAP4 + -0.0216 ASAP6 + 0.0108 ENONCS + 13.8087 FASA- + -2.0303 vsurf_CW2 + 0.3261 vsurf_ID5 | 0.47 | 0.69 | 0.65 | 0.67 | 0.62 | 137.37 | 0.30 | 31.43 |
| 17.3811 + 0.0172 ASAP4 + -0.0213 ASAP6 + 0.0112 ENONCS + -13.9251 FASA+ + -2.0617 vsurf_CW2 + 0.4900 vsurf_ID1 | 0.48 | 0.69 | 0.65 | 0.67 | 0.62 | 137.41 | 0.30 | 31.41 |
| 16.6098 + 0.0159 ASAP4 + 0.01479 ENONCS + -12.7073 FASA+ + -2.1018 vsurf_CW2 + -0.2835 vsurf_HB8 + 0.2866 vsurf_ID8 | 0.48 | 0.69 | 0.64 | 0.67 | 0.62 | 137.44 | 0.30 | 31.40 |
| 16.7541 + 0.0148 ASAP4 + 0.0158 ENONCS + -12.8829 FASA+ + -2.1844 vsurf_CW2 + -0.0801 vsurf_HB7 + 0.4325 vsurf_ID7 | 0.48 | 0.69 | 0.64 | 0.67 | 0.62 | 137.47 | 0.30 | 31.38 |
| 3.2857 + 0.0176 ASAP4 + -0.0224 ASAP6 + 0.0106 ENONCS + 13.9634 FASA- + -1.9613 vsurf_CW2 + 0.3679 vsurf_ID4 | 0.48 | 0.69 | 0.65 | 0.67 | 0.62 | 137.64 | 0.30 | 31.30 |
| 17.2337 + 0.0174 ASAP4 + -0.0227 ASAP6 + 0.0106 ENONCS + -13.9063 FASA+ + -1.9823 vsurf_CW2 + 0.4495 vsurf_ID3 | 0.48 | 0.69 | 0.65 | 0.67 | 0.62 | 137.69 | 0.30 | 31.27 |
| 3.81089 + 0.0132 ASAP4 + 0.0134 ENONCS + 13.2664 FASA- + -2.2608 vsurf_CW2 + 0.2812 vsurf_ID6 + 0.1815 vsurf_ID8 | 0.48 | 0.69 | 0.64 | 0.67 | 0.62 | 137.72 | 0.30 | 31.26 |
| 3.7763 + 0.0134 ASAP4 + 0.0135 ENONCS + 13.0249 FASA- + -2.2318 vsurf_CW2 + 0.2835 vsurf_ID5 + 0.2220 vsurf_ID8 | 0.48 | 0.69 | 0.64 | 0.67 | 0.62 | 137.73 | 0.30 | 31.25 |
| 3.7029 + 0.0169 ASAP4 + -0.0184 ASAP6 + 0.0115 ENONCS + 13.7889 FASA- + -2.0988 vsurf_CW2 + 0.2197 vsurf_ID8 | 0.48 | 0.69 | 0.64 | 0.67 | 0.61 | 138.05 | 0.30 | 31.09 |
| 3.9537 + 0.0138 ASAP4 + 0.01447 ENONCS + 12.7887 FASA- + -2.2702 vsurf_CW2 + 0.1720 vsurf_ID5 + 0.3045 vsurf_ID7 | 0.48 | 0.69 | 0.64 | 0.67 | 0.62 | 138.06 | 0.30 | 31.09 |
| 3.8553 + 0.0136 ASAP4 + 0.0144 ENONCS + 12.8671 FASA- + -2.2318 vsurf_CW2 + 0.2196 vsurf_ID3 + 0.3074 vsurf_ID7 | 0.48 | 0.69 | 0.64 | 0.67 | 0.62 | 138.14 | 0.30 | 31.05 |

| | | | | | | | | |
|---|---|---|---|---|---|---|---|---|
| -1.2384 + 0.0100 ENONCS + 14.4532 FASA- + -2.1107 vsurf_CW2 + -0.9061 vsurf_EWmin3 + -1.3269 vsurf_HB8 + 0.2457 vsurf_ID8 | 0.48 | 0.69 | 0.64 | 0.67 | 0.62 | 138.15 | 0.30 | 31.04 |
| -1.2381 + 0.0100 ENONCS + 14.4531 FASA- + -2.1105 vsurf_CW2 + -0.9060 vsurf_EWmin3 + 0.2457 vsurf_ID8 + -1.3268 vsurf_W8 | 0.48 | 0.69 | 0.64 | 0.67 | 0.62 | 138.16 | 0.30 | 31.04 |
| 3.8938 + 0.0136 ASAP4 + 0.0144 ENONCS + 12.866 FASA- + -2.2372 vsurf_CW2 + 0.2598 vsurf_ID2 + 0.2741 vsurf_ID7 | 0.48 | 0.69 | 0.64 | 0.67 | 0.62 | 138.20 | 0.30 | 31.02 |
| 3.7125 + 0.0132 ASAP4 + 0.0136 ENONCS + 13.1081 FASA- + -2.1831 vsurf_CW2 + 0.4000 vsurf_ID2 + 0.1824 vsurf_ID8 | 0.48 | 0.69 | 0.64 | 0.67 | 0.61 | 138.23 | 0.30 | 31.01 |
| 3.5055 + 0.0147 ASAP4 + 0.0147 ENONCS + 13.4735 FASA- + -2.0232 vsurf_CW2 + -0.1083 vsurf_HB7 + 0.3453 vsurf_ID8 | 0.48 | 0.69 | 0.64 | 0.67 | 0.61 | 138.23 | 0.30 | 31.00 |
| 4.4934 + 0.0147 ASAP4 + 0.0153 ENONCS + 12.7133 FASA- + -2.2006 vsurf_CW2 + 0.1012 vsurf_EWmin3 + 0.3807 vsurf_ID7 | 0.48 | 0.69 | 0.63 | 0.67 | 0.61 | 138.30 | 0.30 | 30.97 |
| 3.1753 + -0.0181 ASAP6 + 0.0086 ENONCS + 15.601 FASA- + -0.5373 dipole + -2.02207 vsurf_CW2 + 0.7827 vsurf_ID2 | 0.48 | 0.69 | 0.65 | 0.67 | 0.62 | 138.31 | 0.30 | 30.96 |
| -0.8490 + 0.0107 ENONCS + 14.232 FASA- + -2.1837 vsurf_CW2 + -0.8564 vsurf_EWmin3 + -1.2036 vsurf_HB8 + 0.3071 vsurf_ID7 | 0.48 | 0.69 | 0.65 | 0.67 | 0.62 | 138.38 | 0.30 | 30.93 |
| -0.8488 + 0.0107 ENONCS + 14.2318 FASA- + -2.1836 vsurf_CW2 + -0.8563 vsurf_EWmin3 + 0.3071 vsurf_ID7 + -1.2035 vsurf_W8 | 0.48 | 0.69 | 0.65 | 0.67 | 0.62 | 138.38 | 0.30 | 30.93 |
| 4.2733 + 0.0140 ASAP4 + 0.0146 ENONCS + 13.2435 FASA- + -2.4522 vsurf_CW2 + 0.3266 vsurf_ID7 + 0.05844 vsurf_Wp5 | 0.48 | 0.69 | 0.64 | 0.67 | 0.61 | 138.40 | 0.30 | 30.92 |
| 3.6467 + 0.0134 ASAP4 + 0.0134 ENONCS + 13.1311 FASA- + -2.1744 vsurf_CW2 + 0.3036 vsurf_ID4 + 0.2237 vsurf_ID8 | 0.48 | 0.69 | 0.64 | 0.67 | 0.61 | 138.46 | 0.30 | 30.89 |
| 17.2445 + 0.0177 ASAP4 + -0.0222 ASAP6 + 0.0106 ENONCS + -13.8682 FASA+ + -1.9935 vsurf_CW2 + 0.3725 vsurf_ID4 | 0.48 | 0.69 | 0.65 | 0.67 | 0.62 | 138.50 | 0.30 | 30.87 |
| 3.7076 + 0.0132 ASAP4 + 0.0137 ENONCS + 13.2219 FASA- + -2.2058 vsurf_CW2 + 0.3801 vsurf_ID1 + 0.1955 vsurf_ID8 | 0.48 | 0.69 | 0.64 | 0.67 | 0.61 | 138.59 | 0.30 | 30.82 |
| 16.7897 + 0.0136 ASAP4 + 0.0135 ENONCS + -12.9264 FASA+ + -2.2601 vsurf_CW2 + 0.2900 vsurf_ID5 + 0.2181 vsurf_ID8 | 0.48 | 0.69 | 0.64 | 0.67 | 0.61 | 138.59 | 0.30 | 30.82 |

| | | | | | | | | |
|---|---|---|---|---|---|---|---|---|
| 17.0699 + 0.0134 ASAP4 + 0.0134 ENONCS + -13.1681 FASA+ + -2.2899 vsurf_CW2 + 0.2865 vsurf_ID6 + 0.1770 vsurf_ID8 | 0.48 | 0.69 | 0.64 | 0.67 | 0.61 | 138.64 | 0.30 | 30.80 |
| 3.4433 + 0.0137 ASAP4 + 0.0150 ENONCS + 12.7794 FASA- + 0.7342 npr2 + -2.2580 vsurf_CW2 + 0.3780 vsurf_ID7 | 0.48 | 0.69 | 0.64 | 0.67 | 0.61 | 138.65 | 0.30 | 30.79 |
| 3.9655 + 0.0137 ASAP4 + 0.0142 ENONCS + 12.9644 FASA- + -2.2737 vsurf_CW2 + 0.1623 vsurf_ID6 + 0.2669 vsurf_ID7 | 0.48 | 0.69 | 0.64 | 0.67 | 0.61 | 138.67 | 0.30 | 30.79 |
| 3.9191 + 0.0137 ASAP4 + 0.0145 ENONCS + 12.9137 FASA- + -2.2533 vsurf_CW2 + 0.21264 vsurf_ID1 + 0.2924 vsurf_ID7 | 0.48 | 0.69 | 0.64 | 0.67 | 0.61 | 138.72 | 0.30 | 30.76 |
| -28.7011 + 0.0139 ASAP4 + 0.0150 ENONCS + 32.668 FASA+ + 45.3441 FASA- + -2.1992 vsurf_CW2 + 0.3757 vsurf_ID7 | 0.48 | 0.69 | -0.32 | 0.67 | -0.42 | 138.73 | 0.30 | 30.75 |
| 3.6351 + 0.0132 ASAP4 + 0.0134 ENONCS + 13.1558 FASA- + -2.1575 vsurf_CW2 + 0.3382 vsurf_ID3 + 0.2109 vsurf_ID8 | 0.48 | 0.69 | 0.64 | 0.67 | 0.61 | 138.84 | 0.31 | 30.70 |
| 16.7052 + 0.0138 ASAP4 + 0.0145 ENONCS + -12.7598 FASA+ + -2.2587 vsurf_CW2 + 0.2233 vsurf_ID3 + 0.3047 vsurf_ID7 | 0.48 | 0.69 | 0.64 | 0.67 | 0.61 | 139.08 | 0.31 | 30.58 |
| 4.0867 + 0.0108 ASAP4 + 0.0131 ENONCS + 13.5817 FASA- + -0.3488 dipole + -2.2744 vsurf_CW2 + 0.4407 vsurf_ID6 | 0.48 | 0.69 | 0.64 | 0.67 | 0.61 | 139.09 | 0.31 | 30.58 |
| 16.7075 + 0.0139 ASAP4 + 0.0145 ENONCS + -12.7451 FASA+ + -2.2677 vsurf_CW2 + 0.1910 vsurf_ID4 + 0.3102 vsurf_ID7 | 0.48 | 0.69 | 0.64 | 0.67 | 0.61 | 139.11 | 0.31 | 30.57 |
| 16.7404 + 0.0138 ASAP4 + 0.0145 ENONCS + -12.7512 FASA+ + -2.2646 vsurf_CW2 + 0.2606 vsurf_ID2 + 0.2721 vsurf_ID7 | 0.48 | 0.69 | 0.64 | 0.67 | 0.61 | 139.19 | 0.31 | 30.53 |
| 4.1239 + -0.0746 AM1_dipole + 0.0108 ASAP4 + 0.0132 ENONCS + 13.5299 FASA- + -2.2816 vsurf_CW2 + 0.4676 vsurf_ID6 | 0.48 | 0.69 | 0.64 | 0.67 | 0.61 | 139.25 | 0.31 | 30.50 |
| 17.5484 + 0.0142 ASAP4 + 0.0147 ENONCS + -13.1747 FASA+ + -2.4901 vsurf_CW2 + 0.3225 vsurf_ID7 + 0.0614 vsurf_Wp5 | 0.48 | 0.69 | 0.63 | 0.67 | 0.61 | 139.25 | 0.31 | 30.50 |
| 17.4559 + 0.0171 ASAP4 + -0.0182 ASAP6 + 0.0117 ENONCS + -13.6419 FASA+ + -2.1279 vsurf_CW2 + 0.2164 vsurf_ID8 | 0.48 | 0.69 | 0.64 | 0.67 | 0.61 | 139.30 | 0.31 | 30.47 |
| 16.7936 + 0.0135 ASAP4 + 0.0137 ENONCS + -12.9805 FASA+ + -2.2100 vsurf_CW2 + 0.4012 vsurf_ID2 + 0.1788 vsurf_ID8 | 0.48 | 0.69 | 0.64 | 0.67 | 0.61 | 139.32 | 0.31 | 30.46 |

| | | | | | | | | |
|---|---|---|---|---|---|---|---|---|
| 17.1808 + 0.0149 ASAP4 + 0.0153 ENONCS + -12.5928 FASA+ + -2.2281 vsurf_CW2 + 0.1006 vsurf_EWmin3 + 0.3790 vsurf_ID7 | 0.48 | 0.69 | 0.63 | 0.67 | 0.60 | 139.32 | 0.31 | 30.46 |
| 3.5576 + 0.0151 ASAP4 + 0.0140 ENONCS + 12.9432 FASA- + -1.9907 vsurf_CW2 + -0.1901 vsurf_HB8 + 0.4932 vsurf_ID2 | 0.48 | 0.69 | 0.64 | 0.67 | 0.61 | 139.32 | 0.31 | 30.46 |
| 3.5576 + 0.0151 ASAP4 + 0.0140 ENONCS + 12.9432 FASA- + -1.9907 vsurf_CW2 + 0.4932 vsurf_ID2 + -0.1901 vsurf_W8 | 0.48 | 0.69 | 0.64 | 0.67 | 0.61 | 139.32 | 0.31 | 30.46 |
| 12.1186 + 0.0172 ASAP4 + 0.0141 ENONCS + -12.176 FASA+ + -2.018 vsurf_CW2 + -190.742 vsurf_CW8 + -0.8161 vsurf_EWmin3 | 0.48 | 0.69 | 0.63 | 0.67 | 0.61 | 139.33 | 0.31 | 30.46 |
| 16.9521 + 0.0149 ASAP4 + 0.0147 ENONCS + -13.3411 FASA+ + -2.0513 vsurf_CW2 + -0.1080 vsurf_HB7 + 0.3415 vsurf_ID8 | 0.48 | 0.69 | 0.63 | 0.66 | 0.61 | 139.39 | 0.31 | 30.43 |
| 16.7597 + 0.0135 ASAP4 + 0.0135 ENONCS + -13.0228 FASA+ + -2.2016 vsurf_CW2 + 0.3087 vsurf_ID4 + 0.2200 vsurf_ID8 | 0.48 | 0.69 | 0.64 | 0.66 | 0.61 | 139.41 | 0.31 | 30.42 |
| 3.9312 + 0.0107 ASAP4 + 0.0135 ENONCS + 13.3423 FASA- + -0.3627 dipole + -2.1574 vsurf_CW2 + 0.6524 vsurf_ID2 | 0.48 | 0.69 | 0.64 | 0.66 | 0.61 | 139.42 | 0.31 | 30.41 |
| 16.9259 + 0.0139 ASAP4 + 0.0143 ENONCS + -12.8753 FASA+ + -2.3017 vsurf_CW2 + 0.1708 vsurf_ID6 + 0.2597 vsurf_ID7 | 0.48 | 0.69 | 0.63 | 0.66 | 0.61 | 139.54 | 0.31 | 30.35 |
| 3.5401 + 0.0151 ASAP4 + 0.0140 ENONCS + 12.9194 FASA- + -1.9756 vsurf_CW2 + -29.0219 vsurf_CW8 + 0.4839 vsurf_ID2 | 0.48 | 0.69 | 0.63 | 0.66 | 0.61 | 139.67 | 0.31 | 30.29 |
| 4.0657 + 0.0138 ASAP4 + 0.0131 ENONCS + 13.8306 FASA- + -2.4284 vsurf_CW2 + 0.3117 vsurf_ID6 + 0.0752 vsurf_Wp5 | 0.48 | 0.69 | 0.63 | 0.66 | 0.61 | 139.67 | 0.31 | 30.29 |
| 16.9038 + 0.0135 ASAP4 + 0.0138 ENONCS + -13.0952 FASA+ + -2.2331 vsurf_CW2 + 0.3810 vsurf_ID1 + 0.1919 vsurf_ID8 | 0.48 | 0.69 | 0.63 | 0.66 | 0.61 | 139.68 | 0.31 | 30.28 |
| 16.8143 + 0.0139 ASAP4 + 0.0146 ENONCS + -12.8001 FASA+ + -2.2808 vsurf_CW2 + 0.2137 vsurf_ID1 + 0.2903 vsurf_ID7 | 0.48 | 0.69 | 0.63 | 0.66 | 0.61 | 139.70 | 0.31 | 30.28 |
| 3.9528 + -0.0756 AM1_dipole + 0.0108 ASAP4 + 0.0136 ENONCS + 13.2685 FASA- + -2.1550 vsurf_CW2 + 0.6867 vsurf_ID2 | 0.48 | 0.69 | 0.63 | 0.66 | 0.61 | 139.73 | 0.31 | 30.26 |
| 4.071 + 0.0140 ASAP4 + 0.0147 ENONCS + 12.8097 FASA- + -2.2694 vsurf_CW2 + 0.3393 vsurf_ID7 + 0.0302 vsurf_ID8 | 0.48 | 0.69 | 0.63 | 0.66 | 0.60 | 139.75 | 0.31 | 30.25 |

| | | | | | | | | |
|---|---|---|---|---|---|---|---|---|
| 4.1203 + 0.0138 ASAP4 + 0.0148 ENONCS + 12.8075 FASA- + -0.0380 dipole + -2.2772 vsurf_CW2 + 0.3695 vsurf_ID7 | 0.48 | 0.69 | 0.63 | 0.66 | 0.61 | 139.75 | 0.31 | 30.25 |
| 3.2663 + -0.1237 AM1_dipole + -0.0175 ASAP6 + 0.0091 ENONCS + 15.6863 FASA- + -2.0701 vsurf_CW2 + 0.8325 vsurf_ID1 | 0.48 | 0.69 | 0.64 | 0.66 | 0.61 | 139.76 | 0.31 | 30.24 |
| 4.1023 + -0.0036 AM1_dipole + 0.0139 ASAP4 + 0.0148 ENONCS + 12.7895 FASA- + -2.2744 vsurf_CW2 + 0.3710 vsurf_ID7 | 0.48 | 0.69 | 0.63 | 0.66 | 0.60 | 139.79 | 0.31 | 30.23 |
| 16.768 + 0.0134 ASAP4 + 0.0135 ENONCS + -13.0372 FASA+ + -2.1845 vsurf_CW2 + 0.3415 vsurf_ID3 + 0.2072 vsurf_ID8 | 0.48 | 0.69 | 0.63 | 0.66 | 0.61 | 139.86 | 0.31 | 30.20 |
| 3.21 + -0.0167 ASAP6 + 0.0090 ENONCS + 15.7525 FASA- + -0.5573 dipole + -2.0637 vsurf_CW2 + 0.7610 vsurf_ID1 | 0.48 | 0.69 | 0.64 | 0.66 | 0.62 | 139.87 | 0.31 | 30.19 |
| 18.6973 + -0.1159 AM1_dipole + -0.0185 ASAP6 + 0.0088 ENONCS + -15.3617 FASA+ + -2.0532 vsurf_CW2 + 0.8426 vsurf_ID2 | 0.48 | 0.69 | 0.64 | 0.66 | 0.61 | 139.90 | 0.31 | 30.18 |
| 17.6413 + 0.0111 ASAP4 + 0.0132 ENONCS + -13.4742 FASA+ + -0.3352 dipole + -2.3026 vsurf_CW2 + 0.4409 vsurf_ID6 | 0.48 | 0.69 | 0.63 | 0.66 | 0.61 | 140.00 | 0.31 | 30.13 |
| 18.7201 + -0.0177 ASAP6 + 0.0087 ENONCS + -15.4384 FASA+ + -0.5220 dipole + -2.0509 vsurf_CW2 + 0.7761 vsurf_ID2 | 0.48 | 0.69 | 0.64 | 0.66 | 0.62 | 140.09 | 0.31 | 30.08 |
| 3.4943 + -0.1100 AM1_dipole + -0.0144 ASAP6 + 0.0087 ENONCS + 15.6116 FASA- + -2.1719 vsurf_CW2 + 0.5353 vsurf_ID6 | 0.48 | 0.68 | 0.64 | 0.66 | 0.61 | 140.13 | 0.31 | 30.07 |
| 17.6306 + -0.0719 AM1_dipole + 0.0111 ASAP4 + 0.0132 ENONCS + -13.4279 FASA+ + -2.3098 vsurf_CW2 + 0.4671 vsurf_ID6 | 0.48 | 0.68 | 0.63 | 0.66 | 0.61 | 140.13 | 0.31 | 30.06 |
| 17.9553 + 0.0139 ASAP4 + 0.0131 ENONCS + -13.8029 FASA+ + -2.4701 vsurf_CW2 + 0.3139 vsurf_ID6 + 0.0777 vsurf_Wp5 | 0.48 | 0.68 | 0.63 | 0.66 | 0.61 | 140.20 | 0.31 | 30.03 |
| 3.7154 + 0.0148 ASAP4 + 0.0136 ENONCS + 13.1628 FASA- + -2.0947 vsurf_CW2 + -0.1470 vsurf_HB8 + 0.3212 vsurf_ID6 | 0.48 | 0.68 | 0.63 | 0.66 | 0.61 | 140.22 | 0.31 | 30.02 |
| 3.7154 + 0.0148 ASAP4 + 0.0136 ENONCS + 13.1628 FASA- + -2.0947 vsurf_CW2 + 0.3212 vsurf_ID6 + -0.1470 vsurf_W8 | 0.48 | 0.68 | 0.63 | 0.66 | 0.61 | 140.22 | 0.31 | 30.02 |
| 3.4225 + -0.0139 ASAP6 + 0.0087 ENONCS + 15.6775 FASA- + -0.4973 dipole + -2.1566 vsurf_CW2 + 0.4921 vsurf_ID6 | 0.48 | 0.68 | 0.64 | 0.66 | 0.61 | 140.27 | 0.31 | 30.00 |

| | | | | | | | | |
|---|---|---|---|---|---|---|---|---|
| 2.6214 + -0.1765 AM1_HOMO + 0.0151 ASAP4 + 0.0137 ENONCS + 12.5915 FASA- + -2.3537 vsurf_CW2 + 0.3549 vsurf_ID6 | 0.48 | 0.68 | 0.64 | 0.66 | 0.61 | 140.28 | 0.31 | 29.99 |
| 16.4855 + 0.0152 ASAP4 + 0.0141 ENONCS + -12.8304 FASA+ + -2.0202 vsurf_CW2 + -0.1885 vsurf_HB8 + 0.4924 vsurf_ID2 | 0.48 | 0.68 | 0.63 | 0.66 | 0.60 | 140.29 | 0.31 | 29.99 |
| 16.4855 + 0.0152 ASAP4 + 0.01411 ENONCS + -12.8304 FASA+ + -2.0202 vsurf_CW2 + 0.4924 vsurf_ID2 + -0.1885 vsurf_W8 | 0.48 | 0.68 | 0.63 | 0.66 | 0.60 | 140.29 | 0.31 | 29.99 |
| 4.5599 + 0.0150 ASAP4 + 0.0144 ENONCS + 13.0224 FASA- + -2.0813 vsurf_CW2 + 0.1514 vsurf_EWmin3 + 0.2832 vsurf_ID8 | 0.48 | 0.68 | 0.62 | 0.66 | 0.59 | 140.31 | 0.31 | 29.98 |
| 3.9322 + 0.0106 ASAP4 + 0.0137 ENONCS + 13.5494 FASA- + -0.3853 dipole + -2.1882 vsurf_CW2 + 0.6423 vsurf_ID1 | 0.48 | 0.68 | 0.63 | 0.66 | 0.61 | 140.33 | 0.31 | 29.97 |
| 2.9607 + 0.0133 ASAP4 + 0.0134 ENONCS + 13.3293 FASA- + 0.9231 npr2 + -2.1633 vsurf_CW2 + 0.3784 vsurf_ID6 | 0.48 | 0.68 | 0.63 | 0.66 | 0.61 | 140.34 | 0.31 | 29.96 |
| 3.0113 + -0.0188 ASAP6 + 0.0082 ENONCS + 15.7388 FASA- + -0.5703 dipole + -1.9406 vsurf_CW2 + 0.7067 vsurf_ID3 | 0.48 | 0.68 | 0.64 | 0.66 | 0.61 | 140.37 | 0.31 | 29.95 |
| 3.6971 + 0.0148 ASAP4 + 0.0136 ENONCS + 13.1409 FASA- + -2.0818 vsurf_CW2 + -22.775 vsurf_CW8 + 0.3191 vsurf_ID6 | 0.48 | 0.68 | 0.63 | 0.66 | 0.61 | 140.37 | 0.31 | 29.95 |
| 3.04313 + -0.1252 AM1_dipole + -0.0198 ASAP6 + 0.0081 ENONCS + 15.6719 FASA- + -1.9335 vsurf_CW2 + 0.7698 vsurf_ID3 | 0.48 | 0.68 | 0.64 | 0.66 | 0.61 | 140.44 | 0.31 | 29.92 |
| 3.4355 + 0.0116 ENONCS + 14.594 FASA- + -0.5546 dipole + -2.0072 vsurf_CW2 + -0.1893 vsurf_HB8 + 0.7404 vsurf_ID2 | 0.48 | 0.68 | 0.64 | 0.66 | 0.62 | 140.52 | 0.31 | 29.87 |

Table S6: GA/MLR equations for model 5

| Equation | RMSE | $R^2$ | $Q^2$ | adj$R^2$ | x_adj$R^2$ | AIC | LOF | F |
|---|---|---|---|---|---|---|---|---|
| 5.3407 + 1.1855 F1 + 0.6009 F3-5 + 0.8497 F7-8 + 1.3077 F9 + -6.3703 FASA+ + 3.6759 FASA_H | 0.40 | 0.76 | 0.72 | 0.74 | 0.70 | 102.52 | 0.22 | 42.21 |
| 9.0166 + 1.1855 F1 + 0.6009 F3-5 + 0.8497 F7-8 + 1.3077 F9 + -6.3704 FASA+ + -3.6759 FASA_P | 0.40 | 0.76 | 0.72 | 0.74 | 0.70 | 102.52 | 0.22 | 42.21 |

| | | | | | | | | |
|---|---|---|---|---|---|---|---|---|
| 2.8760 + 1.2039 F1 + 0.6275 F3-5 + 0.8789 F7-8 + 1.3137 F9 + 5.6060 FASA- + -3.5525 FASA_P | 0.41 | 0.75 | 0.71 | 0.73 | 0.69 | 105.79 | 0.22 | 40.14 |
| 3.6758 + -0.0082 ASA+ + 0.0079 ASA_H + 1.2543 F1 + 0.5913 F3-5 + 0.9372 F7-8 + 1.3861 F9 | 0.40 | 0.76 | 0.71 | 0.74 | 0.69 | 102.73 | 0.22 | 42.07 |
| 25.1332 + -0.0100 ASA_P + 0.0256 DASA + 0.9359 F1 + 0.7601 F7-8 + 1.2585 F9 + -35.8841 FASA+ | 0.40 | 0.76 | 0.71 | 0.74 | 0.69 | 103.54 | 0.22 | 41.56 |
| 5.9950 + 1.1335 F1 + 0.7985 F7-8 + 1.3380 F9 + -7.8317 FASA+ + -3.9089 FASA_P + -0.0338 S | 0.41 | 0.75 | 0.71 | 0.73 | 0.69 | 105.83 | 0.22 | 40.12 |
| 4.6164 + 1.1750 F1 + 0.7712 F7-8 + 1.3432 F9 + -8.3843 FASA+ + 4.3054 FASA_H + 0.9976 std_dim2 | 0.41 | 0.75 | 0.71 | 0.73 | 0.69 | 106.35 | 0.23 | 39.80 |
| 8.9218 + 1.1750 F1 + 0.7712 F7-8 + 1.3432 F9 + -8.3843 FASA+ + -4.3054 FASA_P + 0.9976 std_dim2 | 0.41 | 0.75 | 0.71 | 0.73 | 0.69 | 106.35 | 0.23 | 39.80 |
| -0.0608 + 0.0072 ASA_H + -0.0023 CASA+ + 1.2948 F1 + 0.9290 F7-8 + 1.4497 F9 + 0.0130 Weight | 0.40 | 0.76 | 0.71 | 0.74 | 0.68 | 102.53 | 0.22 | 42.20 |
| 7.1681 + 0.0074 ASA_H + 1.1889 F1 + 0.7709 F7-8 + 1.3477 F9 + -6.7431 FASA+ + -0.5160 std_dim1 | 0.40 | 0.77 | 0.70 | 0.75 | 0.68 | 101.43 | 0.21 | 42.92 |
| 0.1213 + 0.0077 ASA_H + -0.0034 CASA- + 1.0906 F1 + 1.0189 F7-8 + 1.3941 F9 + 2.9501 FCASA- | 0.42 | 0.74 | 0.70 | 0.72 | 0.68 | 109.12 | 0.23 | 38.12 |
| 8.0310 + -0.0043 ASA_P + 1.1577 F1 + 0.7836 F3-5 + 0.8716 F7-8 + 1.2025 F9 + -5.5468 FASA+ | 0.42 | 0.74 | 0.70 | 0.72 | 0.68 | 108.97 | 0.23 | 38.21 |
| 2.0269 + -0.0037 DASA + 1.2779 F1 + 0.7711 F3-5 + 0.9619 F7-8 + 1.3313 F9 + 3.2057 FASA_H | 0.41 | 0.74 | 0.70 | 0.72 | 0.68 | 108.74 | 0.23 | 38.34 |
| 0.4924 + 0.0076 ASA_H + 1.1990 F1 + 0.7831 F7-8 + 1.3526 F9 + 6.4798 FASA- + -0.5311 std_dim1 | 0.40 | 0.76 | 0.70 | 0.74 | 0.68 | 103.29 | 0.22 | 41.72 |
| 13.3925 + 0.01307 DASA + 1.0594 F1 + 0.7340 F7-8 + 1.3329 F9 + -22.5323 FASA+ + 4.7447 FASA_H | 0.41 | 0.75 | 0.70 | 0.73 | 0.68 | 106.64 | 0.23 | 39.62 |
| 18.1372 + 0.0130 DASA + 1.0594 F1 + 0.7340 F7-8 + 1.3329 F9 + -22.5323 FASA+ + -4.7447 FASA_P | 0.41 | 0.75 | 0.70 | 0.73 | 0.68 | 106.64 | 0.23 | 39.62 |

| | | | | | | | | |
|---|---|---|---|---|---|---|---|---|
| 3.3553 + 0.0054 ASA_H + 1.3588 F1 + 0.4634 F3-5 + 1.064 F7-8 + 1.4242 F9 + -0.5286 std_dim1 | 0.41 | 0.75 | 0.70 | 0.73 | 0.68 | 106.64 | 0.23 | 39.62 |
| -0.1607 + -0.0084 ASA+ + 0.0090 ASA_H + 1.2500 F1 + 0.9421 F7-8 + 1.4769 F9 + -0.0293 S | 0.41 | 0.75 | 0.70 | 0.73 | 0.67 | 106.80 | 0.23 | 39.52 |
| 8.1251 + 0.0081 ASA_H + 1.2138 F1 + 0.7784 F7-8 + 1.3547 F9 + -7.6737 FASA+ + -0.6216 rgyr | 0.40 | 0.76 | 0.70 | 0.74 | 0.67 | 103.99 | 0.22 | 41.27 |
| 9.4918 + 1.1882 F1 + 0.7677 F7-8 + 1.2828 F9 + -8.3443 FASA+ + -4.1267 FASA_P + 0.9502 std_dim3 | 0.42 | 0.74 | 0.70 | 0.72 | 0.67 | 109.78 | 0.24 | 37.73 |
| 4.3611 + -0.0039 ASA_P + 1.3545 F1 + 0.9630 F3-5 + 1.0920 F7-8 + 1.2844 F9 + -0.6244 vsurf_Wp6 | 0.42 | 0.74 | 0.70 | 0.72 | 0.67 | 111.30 | 0.24 | 36.84 |
| 9.8257 + 1.1408 F1 + 0.5255 F4 + 0.7596 F7-8 + 1.2965 F9 + -7.4592 FASA+ + -3.8699 FASA_P | 0.42 | 0.74 | 0.69 | 0.72 | 0.67 | 110.98 | 0.24 | 37.02 |
| 2.7433 + -0.0041 ASA_P + 1.1794 F1 + 0.8032 F3-5 + 0.9032 F7-8 + 1.2145 F9 + 4.7268 FASA- | 0.42 | 0.74 | 0.69 | 0.72 | 0.67 | 111.80 | 0.24 | 36.54 |
| 3.8387 + 0.0057 ASA_H + 1.4056 F1 + 0.5528 F3-5 + 1.1120 F7-8 + 1.437 F9 + -0.6394 rgyr | 0.42 | 0.74 | 0.69 | 0.72 | 0.67 | 109.12 | 0.23 | 38.12 |
| 21.7603 + -0.0091 ASAN5 + 1.0519 F1 + 0.6099 F7-8 + 1.3223 F9 + -8.6024 FASA+ + -4.3488 vsurf_CW1 | 0.42 | 0.74 | 0.69 | 0.72 | 0.67 | 109.46 | 0.23 | 37.92 |
| 1.2042 + 1.4036 F1 + 0.6466 F3-5 + 1.1267 F7-8 + 1.4390 F9 + 3.1727 FASA_H + 0.8153 npr1 | 0.42 | 0.74 | 0.69 | 0.72 | 0.67 | 111.47 | 0.24 | 36.74 |
| 2.2514 + -0.0070 ASA+ + 0.0097 ASA_H + 1.3750 F1 + 0.9755 F7-8 + 1.5040 F9 + 1.3877 npr1 | 0.41 | 0.75 | 0.69 | 0.73 | 0.67 | 107.65 | 0.23 | 39.00 |
| 2.0302 + -0.0094 ASA+ + 0.00972 ASA_H + 1.2881 F1 + 0.9196 F7-8 + 1.475 F9 + 0.8950 std_dim2 | 0.41 | 0.75 | 0.69 | 0.73 | 0.67 | 107.86 | 0.23 | 38.88 |
| 8.8911 + 0.0043 ASA_H + -0.0041 ENONS + 1.3841 F1 + 1.0197 F7-8 + 1.4863 F9 + -9.4871 FASA+ | 0.41 | 0.75 | 0.69 | 0.73 | 0.67 | 105.92 | 0.22 | 40.06 |
| 2.9753 + 0.0088 ASA_H + -0.0056 DASA + 1.2767 F1 + 0.8354 F7-8 + 1.4180 F9 + -0.4711 std_dim1 | 0.41 | 0.75 | 0.69 | 0.74 | 0.67 | 105.20 | 0.22 | 40.51 |

| | | | | | | | | |
|---|---|---|---|---|---|---|---|---|
| 2.2606 + 0.0062 ASA_H + -0.0005 CASA+ + 1.2704 F1 + 0.5921 F3-5 + 1.0420 F7-8 + 1.4266 F9 | 0.42 | 0.74 | 0.69 | 0.72 | 0.67 | 111.37 | 0.24 | 36.80 |
| 14.0528 + 0.0013 DCASA + 1.0986 F1 + 0.7317 F7-8 + 1.3311 F9 + -14.2192 FASA+ + -4.8941 FASA_P | 0.41 | 0.75 | 0.69 | 0.73 | 0.67 | 107.86 | 0.23 | 38.87 |
| 0.5554 + 0.0081 ASA_H + 1.2268 F1 + 0.7973 F7-8 + 1.3633 F9 + 7.1848 FASA- + -0.6058 rgyr | 0.41 | 0.75 | 0.69 | 0.73 | 0.67 | 107.42 | 0.23 | 39.14 |
| 2.4386 + 0.0122 ASA- + -0.0075 ASA_P + 1.2730 F1 + 0.8097 F7-8 + 1.3957 F9 + 1.2662 npr1 | 0.41 | 0.75 | 0.69 | 0.73 | 0.66 | 106.96 | 0.23 | 39.42 |
| 0.8922+ 0.0087 ASA_H + -0.0005 CASA+ + 1.3935 F1 + 1.0680 F7-8 + 1.5429 F9 + 1.5899 npr1 | 0.42 | 0.74 | 0.69 | 0.72 | 0.66 | 111.75 | 0.24 | 36.58 |
| 1.3952 + 0.0071 ASA_H + -0.0011 DCASA + 1.3427 F1 + 0.8880 F7-8 + 1.4684 F9 + 1.42061 npr1 | 0.41 | 0.74 | 0.69 | 0.72 | 0.66 | 108.78 | 0.23 | 38.33 |
| 4.7089 + 1.2035 F1 + 0.7397 F7-8 + 1.3539 F9 + -8.1531 FASA+ + 5.4634 FASA_H + 0.0029 vsurf_HB3 | 0.42 | 0.74 | 0.68 | 0.72 | 0.66 | 111.47 | 0.24 | 36.74 |
| 10.1723 + 1.2035 F1 + 0.7397 F7-8 + 1.3539 F9 + -8.1531 FASA+ + -5.4634 FASA_P + 0.0029 vsurf_HB3 | 0.42 | 0.74 | 0.68 | 0.72 | 0.66 | 111.47 | 0.24 | 36.74 |
| -0.5445 + 0.0045 ASA_H + -0.0041 ENONS + 1.3903 F1 + 1.028 F7-8 + 1.4884 F9 + 9.1887 FASA- | 0.42 | 0.74 | 0.68 | 0.72 | 0.66 | 108.96 | 0.23 | 38.21 |
| 3.0080 + 0.0053 ASA_H + 1.2702 F1 + 0.6655 F3-5 + 0.9739 F7-8 + 1.3987 F9 + -0.5274 FCASA+ | 0.42 | 0.74 | 0.68 | 0.71 | 0.66 | 111.81 | 0.24 | 36.54 |
| 2.4411 + -0.0103 ASA+ + 0.0119 ASA_H + 1.3085 F1 + 0.9066 F7-8 + 1.4536 F9 + 2.7393 vsurf_CW5 | 0.42 | 0.74 | 0.68 | 0.72 | 0.66 | 108.79 | 0.23 | 38.32 |
| -0.1924 + 1.2452 F1 + 0.9062 F7-8 + 1.3371 F9 + -8.1485 FASA+ + 3.76603 FASA_H + 4.6275 vsurf_R | 0.42 | 0.73 | 0.68 | 0.71 | 0.66 | 111.86 | 0.24 | 36.51 |
| 2.3083 + 0.0058 ASA_H + -0.0059 DASA + 1.2493 F1 + 0.7763 F7-8 + 1.3791 F9 + 1.1790 npr1 | 0.41 | 0.75 | 0.68 | 0.73 | 0.66 | 107.30 | 0.23 | 39.22 |
| -0.4470 + -0.0099 ASA_P + 1.1523 F1 + 0.8376 F7-8 + 1.3272 F9 + 11.2791 FASA- + 0.9781 FCASA+ | 0.42 | 0.74 | 0.68 | 0.72 | 0.66 | 111.81 | 0.24 | 36.54 |

| | | | | | | | | |
|---|---|---|---|---|---|---|---|---|
| 11.0126 + 0.0092 ASA+ + -0.0090 ASA_P + 1.0937 F1 + 0.7398 F7-8 + 1.3411 F9 + -13.3046 FASA+ | 0.42 | 0.74 | 0.68 | 0.72 | 0.66 | 109.05 | 0.23 | 38.16 |
| 3.2390 + 0.0064 ASA_H + 1.3586 F1 + 0.3739 F4 + 1.0359 F7-8 + 1.4574 F9 + -0.5968 std_dim1 | 0.42 | 0.74 | 0.68 | 0.72 | 0.66 | 110.87 | 0.24 | 37.08 |
| 11.6046 + -0.0107 ASA- + 0.0098 ASA_H + 1.1053 F1 + 0.7620 F7-8 + 1.3546 F9 + -14.0886 ASA+ | 0.41 | 0.75 | 0.68 | 0.73 | 0.66 | 107.46 | 0.23 | 39.12 |
| -1.6722 + -0.0024 CASA- + 1.1889 F1 + 0.9599 F7-8 + 1.4048 F9 + 5.0324 FASA_H + 3.1976 FCASA- | 0.41 | 0.75 | 0.68 | 0.73 | 0.66 | 107.51 | 0.23 | 39.09 |
| 6.2836 + 1.1639 F1 + 0.7644 F7-8 + 1.3660 F9 + -10.243 FASA+ + 4.7864 FASA_H + 0.4794 FCASA+ | 0.41 | 0.74 | 0.68 | 0.73 | 0.66 | 108.72 | 0.23 | 38.36 |
| 11.07 + 1.1639 F1 + 0.7644 F7-8 + 1.3660 F9 + -10.243 FASA+ + -4.7864 FASA_P + 0.4794 FCASA+ | 0.41 | 0.74 | 0.68 | 0.73 | 0.66 | 108.72 | 0.23 | 38.36 |
| 3.4368 + -0.0084 ASA+ + 0.0089 ASA_H + 1.2469 F1 + 0.4578 F4 + 0.8950 F7-8 + 1.4287 F9 | 0.42 | 0.74 | 0.68 | 0.72 | 0.66 | 111.09 | 0.24 | 36.96 |
| 2.5060 + -0.0088 ASA+ + 0.0096 ASA_H + 1.3053 F1 + 0.9262 F7-8 + 1.4421 F9 + 0.7939 std_dim3 | 0.42 | 0.74 | 0.68 | 0.72 | 0.66 | 110.29 | 0.24 | 37.43 |
| 6.1719 + 0.0042 ASA_H + 1.1758 F1 + 0.7413 F7-8 + 1.3244 F9 + -6.3264 FASA+ + 1.1085 npr1 | 0.41 | 0.75 | 0.68 | 0.73 | 0.65 | 108.07 | 0.23 | 38.75 |
| 2.3539 + -0.0069 ASA+ + 0.0101 ASA_H + 1.3180 F1 + 0.9266 F7-8 + 1.4794 F9 + 1.5155 glob | 0.42 | 0.74 | 0.68 | 0.72 | 0.65 | 110.49 | 0.24 | 37.31 |
| -0.0859 + 0.0043 ASA_H + 1.1882 F1 + 0.7557 F7-8 + 1.3304 F9 + 5.9866 FASA- + 1.1592 npr1 | 0.42 | 0.74 | 0.68 | 0.72 | 0.65 | 109.84 | 0.24 | 37.69 |
| -0.1556 + 0.0055 ASA_H + -0.0023 CASA+ + 1.2583 F1 + 1.0385 F7-8 + 1.4488 F9 + 0.0158 vol | 0.42 | 0.74 | 0.68 | 0.72 | 0.65 | 110.72 | 0.24 | 37.17 |
| 8.7697 + -0.0085 ASA_P + 1.1580 F1 + 0.7957 F7-8 + 1.3605 F9 + -8.3278 FASA+ + 0.0060 VSA | 0.42 | 0.74 | 0.68 | 0.72 | 0.65 | 108.80 | 0.23 | 38.31 |
| 6.4703 + 0.0046 ASA_H + 1.1179 F1 + 0.6830 F7-8 + 1.2939 F9 + -6.6547 FASA+ + 1.2944 glob | 0.41 | 0.74 | 0.67 | 0.73 | 0.65 | 108.59 | 0.23 | 38.44 |

| | | | | | | | | |
|---|---|---|---|---|---|---|---|---|
| 2.5642 + 0.0130 ASA- + -0.0076 ASA_P + 1.2053 F1 + 0.7375 F7-8 + 1.3561 F9 + 1.3904 glob | 0.42 | 0.74 | 0.67 | 0.72 | 0.65 | 108.87 | 0.23 | 38.27 |
| 2.4089 + 0.0063 ASA_H + -0.0061 DASA + 1.1952 F1 + 0.7233 F7-8 + 1.3532 F9 + 1.3390 glob | 0.41 | 0.74 | 0.67 | 0.73 | 0.65 | 108.55 | 0.23 | 38.46 |
| 2.9907 + 0.0067 ASA_H + -0.0078 DASA + -0.0033 ENONS + 1.4488 F1 + 1.0356 F7-8 + 1.5434 F9 | 0.42 | 0.74 | 0.67 | 0.72 | 0.65 | 109.87 | 0.24 | 37.68 |
| 7.8865 + -0.0242 ASAP7 + 0.0047 ASA_H + 1.3265 F1 + 0.7564 F7-8 + 1.2700 F9 + -8.2009 FASA+ | 0.41 | 0.75 | 0.67 | 0.73 | 0.65 | 108.47 | 0.23 | 38.51 |
| 0.5301 + 0.0048 ASA_H + -0.0064 DASA + 1.14243 F1 + 0.7369 F7-8 + 1.3480 F9 + -0.0217 S | 0.42 | 0.74 | 0.67 | 0.72 | 0.65 | 108.82 | 0.23 | 38.30 |
| -0.1252 + 0.0047 ASA_H + 1.1270 F1 + 0.6937 F7-8 + 1.2979 F9 + 6.3488 FASA- + 1.3564 glob | 0.42 | 0.74 | 0.67 | 0.72 | 0.65 | 110.39 | 0.24 | 37.37 |
| 7.7501 + 0.0076 ASA_H + 1.1581 F1 + 0.7595 F7-8 + 1.3149 F9 + -7.3137 FASA+ + -0.0027 vsurf_D1 | 0.41 | 0.75 | 0.67 | 0.73 | 0.65 | 108.25 | 0.23 | 38.64 |
| 3.2660 + 0.0041 ASA_H + -0.0057 DASA + 1.1680 F1 + 0.4019 F3-5 + 0.7672 F7-8 + 1.3037 F9 | 0.42 | 0.74 | 0.67 | 0.72 | 0.65 | 108.92 | 0.23 | 38.24 |
| 2.1475 + -0.0098 ASA+ + 0.0120 ASA_H + 1.2590 F1 + 0.8357 F7-8 + 1.474 F9 + 0.7269 vsurf_CW3 | 0.42 | 0.74 | 0.67 | 0.72 | 0.65 | 111.71 | 0.24 | 36.60 |
| 3.1256 + -0.0114 ASA+ + 0.0110 ASA_H + 1.2668 F1 + 0.8235 F7-8 + 1.4320 F9 + 0.0027 vsurf_HB3 | 0.42 | 0.74 | 0.67 | 0.72 | 0.65 | 110.60 | 0.24 | 37.25 |
| 1.1927 + 0.0075 ASA_H + -0.0012 CASA+ + 1.1697 F1 + 0.8116 F7-8 + 1.3962 F9 + 1.2960 FCASA- | 0.42 | 0.74 | 0.67 | 0.72 | 0.64 | 111.34 | 0.24 | 36.81 |
| 3.3018 + -0.0089 ASA+ + 0.0116 ASA_H + 1.2775 F1 + 0.8173 F7-8 + 1.4240 F9 + -0.1576 vsurf_IW6 | 0.42 | 0.74 | 0.67 | 0.72 | 0.64 | 111.04 | 0.24 | 36.99 |
| 2.9309 + -0.0113 ASA+ + 0.0114 ASA_H + 1.2578 F1 + 0.8296 F7-8 + 1.4510 F9 + 0.0021 vsurf_HB2 | 0.42 | 0.74 | 0.67 | 0.72 | 0.64 | 111.10 | 0.24 | 36.95 |
| 3.1378 + -0.0116 ASA+ + 0.0112 ASA_H + 1.2639 F1 + 0.8169 F7-8 + 1.4531 F9 + 0.0023 vsurf_W3 | 0.42 | 0.74 | 0.67 | 0.72 | 0.64 | 111.17 | 0.24 | 36.91 |

| | | | | | | | | |
|---|---|---|---|---|---|---|---|---|
| 2.4756 + 0.0110 ASA- + -0.0082 ASA_P + 1.2507 F1 + 0.8396 F7-8 + 1.3985 F9 + 0.6691 std_dim3 | 0.42 | 0.74 | 0.67 | 0.72 | 0.64 | 110.62 | 0.24 | 37.23 |
| 3.7898 + -0.0178 ASA+ + 0.0111 ASA_H + 1.3026 F1 + 0.9465 F7-8 + 1.4524 F9 + 1.0109 FCASA+ | 0.42 | 0.74 | 0.67 | 0.72 | 0.64 | 111.71 | 0.24 | 36.60 |
| 7.7874 + 0.0085 ASA_H + -0.0008 CASA- + 1.0576 F1 + 0.7438 F7-8 + 1.3404 F9 + -9.6477 FASA+ | 0.41 | 0.75 | 0.67 | 0.73 | 0.64 | 108.16 | 0.23 | 38.69 |
| -0.2698 + -0.0248 ASAP7 + 0.0049 ASA_H + 1.3400 F1 + 0.7683 F7-8 + 1.2727 F9 + 7.9616 FASA- | 0.42 | 0.74 | 0.67 | 0.72 | 0.64 | 110.79 | 0.24 | 37.13 |
| 7.6933 + 0.0080 ASA_H + 1.1433 F1 + 0.7679 F7-8 + 1.3405 F9 + -7.9352 FASA+ + -0.0017 vsurf_Wp1 | 0.42 | 0.74 | 0.67 | 0.72 | 0.64 | 111.15 | 0.24 | 36.92 |
| 7.8986 + 0.0054 ASA_H + -0.0031ASA_P + 1.1340 F1 + 0.7400 F7-8 + 1.3559 F9 + -8.2193* FASA+ | 0.42 | 0.74 | 0.67 | 0.72 | 0.64 | 111.00 | 0.24 | 37.01 |
| 7.8986 + -0.0031 ASA + 0.0086 ASA_H + 1.1340 F1 + 0.7400 F7-8 + 1.3559 F9 + -8.2193 FASA+ | 0.42 | 0.74 | 0.67 | 0.72 | 0.64 | 111.00 | 0.24 | 37.01 |
| 3.1472 + 0.0089 ASA_H + -0.0059 DASA + 1.2975 F1 + 0.8486 F7-8 + 1.4299 F9 + -0.4454 rgyr | 0.42 | 0.74 | 0.67 | 0.72 | 0.64 | 111.15 | 0.24 | 36.92 |
| 2.2251 + 0.0051 ASA_H + -0.0067 DASA + 1.1706 F1 + 0.7181 F7-8 + 1.3451 F9 + 0.5490 std_dim2 | 0.42 | 0.74 | 0.67 | 0.72 | 0.64 | 111.49 | 0.24 | 36.73 |
| 8.3882 + -0.0090 ASA_P + 1.2520 F1 + 0.8221 F7-8 + 1.3710 F9 + -7.5527 FASA+ + 0.0060 Weight | 0.41 | 0.75 | 0.67 | 0.73 | 0.64 | 108.41 | 0.23 | 38.54 |
| 2.9380 + 0.0196 ASAP1 + 0.0062 ASA_H + -0.0083 DASA + 1.1690 F1 + 0.6875 F7-8 + 1.4053 F9 | 0.42 | 0.74 | 0.67 | 0.72 | 0.64 | 111.21 | 0.24 | 36.89 |
| 0.5381 + 0.0077 ASA_H + 1.1719 F1 + 0.7780 F7-8 + 1.3238 F9 + 6.8687 FASA- + -0.0027 vsurf_D1 | 0.42 | 0.74 | 0.67 | 0.72 | 0.64 | 111.16 | 0.24 | 36.92 |
| 2.8640 + -0.0096 ASA_P + 1.3357 F1 + 0.9850 F7-8 + 1.4803 F9 + 1.2622 FCASA- + 0.6352 std_dim2 | 0.42 | 0.74 | 0.66 | 0.72 | 0.64 | 111.04 | 0.24 | 36.99 |
| 3.2340 + 0.0093 ASA_H + -0.0064 DASA + 1.2469 F1 + 0.8134 F7-8 + 1.3858 F9 + -0.0025 vsurf_D1 | 0.42 | 0.74 | 0.66 | 0.72 | 0.64 | 109.92 | 0.24 | 37.64 |

| | | | | | | | | |
|---|---|---|---|---|---|---|---|---|
| 5.5838 + 1.1683 F1 + 0.7516 F7-8 + 1.3662 F9 + -8.5465 FASA+ + 4.2047 FASA_H + 0.0031 VSA | 0.42 | 0.74 | 0.66 | 0.72 | 0.64 | 111.40 | 0.24 | 36.78 |
| 9.7885 + 1.1683 F1 + 0.7516 F7-8 + 1.3662 F9 + -8.5465 FASA+ + -4.2047 FASA_P + 0.0031 VSA | 0.42 | 0.74 | 0.66 | 0.72 | 0.64 | 111.40 | 0.24 | 36.78 |
| 7.7902 + 0.0083 ASA_H + 1.1323 F1 + 0.7353 F7-8 + 1.3514 F9 + -8.1973 FASA+ + -0.0030 vsurf_S | 0.42 | 0.74 | 0.66 | 0.72 | 0.64 | 111.62 | 0.24 | 36.65 |
| 8.3362 + -0.0083 ASA_P + 1.1774 F1 + 0.7962 F7-8 + 1.359 F9 + -8.0110 FASA+ + 0.0031 vsurf_V | 0.42 | 0.74 | 0.66 | 0.72 | 0.63 | 109.98 | 0.24 | 37.61 |
| 7.5593 + 0.0071 ASA_H + 1.1345 F1 + 0.7374 F7-8 + 1.312 F9 + -7.4897 FASA+ + -0.0043 vsurf_D3 | 0.42 | 0.74 | 0.66 | 0.72 | 0.63 | 111.06 | 0.24 | 36.98 |
| 8.7081 + -0.0083 ASA_P + 1.2030 F1 + 0.8288 F7-8 + 1.3548 F9 + -8.2441 FASA+ + 0.0063 vol | 0.42 | 0.74 | 0.66 | 0.72 | 0.63 | 110.20 | 0.24 | 37.48 |
| 5.1981 + 1.2184 F1 + 0.7656 F7-8 + 1.3783 F9 + -8.1431 FASA+ + 4.4223 FASA_H + 0.0030 Weight | 0.42 | 0.74 | 0.66 | 0.72 | 0.63 | 110.49 | 0.24 | 37.31 |
| 6.3906 + 0.0075 ASA_H + -0.0004 CASA+ + 1.1109 F1 + 0.7306 F7-8 + 1.3519 F9 + -7.0819 FASA+ | 0.42 | 0.74 | 0.66 | 0.72 | 0.63 | 111.80 | 0.24 | 36.55 |
| 4.9573 + 0.0034 ASA_H + 1.088 F1 + 0.7178 F7-8 + 1.3030 F9 + -6.5849 FASA+ + -0.0171 S | 0.42 | 0.74 | 0.66 | 0.72 | 0.63 | 111.67 | 0.24 | 36.62 |
| 6.7053 + 0.0029 ASA_H + 1.1158 F1 + 0.3338 F3-5 + 0.74811 F7-8 + 1.2719 F9 + -5.8752 FASA+ | 0.42 | 0.74 | 0.66 | 0.72 | 0.63 | 111.49 | 0.24 | 36.73 |
| 5.5476 + 1.1929 F1 + 0.7706 F7-8 + 1.3660 F9 + -8.5173 FASA+ + 4.2256 FASA_H + 0.0033 vol | 0.42 | 0.74 | 0.65 | 0.72 | 0.63 | 111.78 | 0.24 | 36.56 |
| 3.3630 + -0.0121 ASA+ + 0.0089 ASA_H + 1.3113 F1 + 0.8529 F7-8 + 1.4315 F9 + 0.0045 Weight | 0.42 | 0.73 | 0.64 | 0.71 | 0.61 | 111.93 | 0.24 | 36.47 |
| 2.9759 + -0.0091 ASA_P + -0.0015 CASA- + 1.1921 F1 + 0.9092 F7-8 + 1.3508 F9 + 2.9639 FCASA- | 0.42 | 0.74 | 0.62 | 0.72 | 0.59 | 110.36 | 0.24 | 37.38 |
| 4.4292 + -0.0073 ASA_P + 0.0007 CASA- + 1.3723 F1 + 0.6956 F3-5 + 1.0702 F7-8 + 1.4374 F9 | 0.42 | 0.74 | 0.61 | 0.72 | 0.58 | 110.65 | 0.24 | 37.22 |

Table S7: Validation values of models 1-8 according to Golbraikh and Tropsha [33, 34].

| Model | 1 | 2 | 3 | 4 | 5 | 6 | 7 | 8 |
|---|---|---|---|---|---|---|---|---|
| $Q^2$ | 0.67 | 0.67 | 0.66 | 0.66 | 0.72 | 0.63 | 0.62 | 0.62 |
| $R_{ext}^2$ ($P^2$) | 0.52 | 0.53 | 0.54 | 0.54 | 0.54 | 0.66 | 0.68 | 0.73 |
| $\frac{(R2 - R02)}{R2}$ | 0.02 | 0.00 | 0.08 | 0.08 | 0.08 | 0 | 0 | 0 |
| k | 1.02 | 1.04 | 1.03 | 1.03 | 1.03 | 1 | 1 | 1 |
| K‘ | 0.97 | 0.95 | 0.96 | 0.97 | 0.96 | 0.99 | 0.99 | 0.99 |
| $\lvert R02 - R\grave{}02 \rvert$ | 0.64 | 0.38 | 0.06 | 0.06 | 0.06 | 0.17 | 0.15 | 0.10 |

Table S8: Y- randomized validation values for models 1-5

| Model | Replicate | $R^2$ | $Q^2$ | RMSE |
|---|---|---|---|---|
| Model 1 | 1 | 0.29 | 0.19 | 0.74 |
| | 2 | 0.32 | 0.22 | 0.72 |
| | 3 | 0.28 | 0.13 | 0.77 |
| | 4 | 0.23 | 0.19 | 0.74 |
| | 5 | 0.30 | 0.20 | 0.73 |
| Model 2 | 1 | 0.22 | 0.15 | 0.74 |
| | 2 | 0.27 | 0.18 | 0.73 |
| | 3 | 0.28 | 0.19 | 0.72 |
| | 4 | 0.20 | 0.09 | 0.75 |
| | 5 | 0.25 | 0.13 | 0.65 |
| Model 3 | 1 | 0.04 | -0.16 | 0.79 |
| | 2 | 0.04 | -0.13 | 0.80 |
| | 3 | 0.15 | 0.01 | 0.75 |
| | 4 | 0.11 | -0.06 | 0.79 |
| | 5 | 0.19 | 0.06 | 0.76 |
| Model 4 | 1 | 0.05 | -0.07 | 0.80 |
| | 2 | 0.11 | 0.02 | 0.76 |
| | 3 | 0.14 | 0.02 | 0.78 |
| | 4 | 0.09 | 0.02 | 0.80 |
| | 5 | 0.11 | -0.02 | 0.82 |
| Model 5 | 1 | 0.31 | 0.20 | 0.59 |
| | 2 | 0.34 | 0.24 | 0.70 |
| | 3 | 0.26 | 0.17 | 0.68 |
| | 4 | 0.37 | 0.28 | 0.71 |
| | 5 | 0.22 | 0.13 | 0.77 |

Table S9: Applicability domain (AD) calculations for the test sets of models 1-5 according to Roy et al. [35]. The descriptor values for each model, on which the AD calculations are based, are included.

Model 1 test set

| compound | ASAP4 | ENONCS | FASA+ | npr1 | vsurf_CW2 | vsurf_ID2 | pIC50_Tbr | AD Info. |
|---|---|---|---|---|---|---|---|---|
| **2** | 22.88568 | 44.20015 | 0.572666 | 0.655046 | 2.026807 | 1.06693 | 7.201 | Inside AD |
| **3** | 35.43919 | 43.078 | 0.603196 | 0.560596 | 1.889391 | 1.104301 | 6.979 | Inside AD |
| **7** | 22.93634 | 6.587632 | 0.64951 | 0.477132 | 1.895549 | 1.297377 | 6.04 | Inside AD |
| **8** | 34.93615 | 51.0996 | 0.579584 | 0.376275 | 2.222167 | 1.425896 | 6.496 | Inside AD |
| **14** | 0 | 38.07964 | 0.60874 | 0.46218 | 1.969641 | 0.537138 | 5.93 | Inside AD |
| **25** | 0 | 41.59878 | 0.665519 | 0.302223 | 1.883426 | 0.966754 | 4.627 | Inside AD |
| **27** | 0 | 41.59878 | 0.631827 | 0.175341 | 1.993618 | 0.776929 | 4.97 | Inside AD |
| **37** | 0 | 35.56729 | 0.571087 | 0.491172 | 1.944233 | 0.283745 | 6.321 | Inside AD |
| **49** | 0 | 63.49114 | 0.62379 | 0.288459 | 2.438907 | 0.909478 | 5.368 | Inside AD |
| **51** | 0 | 41.30069 | 0.594261 | 0.27525 | 2.01381 | 0.839873 | 6.164 | Inside AD |
| **54** | 0 | 43.15465 | 0.611733 | 0.28808 | 1.982432 | 1.250651 | 5.746 | Inside AD |
| **60** | 2.795183 | 38.76179 | 0.608446 | 0.354595 | 1.848828 | 1.609684 | 5.681 | Inside AD |
| **72** | 1.133659 | 26.11522 | 0.582402 | 0.679125 | 1.870574 | 1.136925 | 6.788 | Inside AD |
| **73** | 37.13449 | 35.80936 | 0.592159 | 0.408659 | 2.006488 | 0.939815 | 7.142 | Inside AD |
| **77** | 17.33099 | 71.91664 | 0.54154 | 0.677785 | 1.890656 | 0.682982 | 7.114 | Inside AD |
| **78** | 16.63682 | 42.88255 | 0.572957 | 0.684844 | 1.850475 | 0.777458 | 6.588 | Inside AD |
| **80** | 0.974567 | 41.34584 | 0.641433 | 0.454726 | 1.945057 | 0.416341 | 5.85 | Inside AD |
| **81** | 11.89614 | 34.11543 | 0.615075 | 0.481765 | 2.09767 | 0.785576 | 5.704 | Inside AD |
| **83** | 0 | 29.22077 | 0.631182 | 0.497556 | 1.786121 | 0.713247 | 6.084 | Inside AD |
| **84** | 7.671603 | 31.39807 | 0.653324 | 0.428094 | 1.801258 | 0.597414 | 5.496 | Inside AD |
| **86** | 9.986183 | 0 | 0.594943 | 0.570648 | 2.093368 | 0.824407 | 5.767 | Inside AD |
| **88** | 0 | 88.71912 | 0.580841 | 0.399596 | 2.120035 | 0.594585 | 6.593 | Inside AD |
| **90** | 0 | 117.1137 | 0.528332 | 0.348395 | 2.202932 | 1.071369 | 6.7959 | Outside AD |
| **94** | 1.618329 | 33.97212 | 0.650008 | 0.567519 | 2.328978 | 1.045125 | 5.556 | Inside AD |
| **96** | 0 | 39.99709 | 0.628295 | 0.534834 | 2.34578 | 0.94823 | 5.2688 | Inside AD |
| **98** | 0 | 40.5323 | 0.639398 | 0.536641 | 2.088417 | 1.379285 | 5.9431 | Inside AD |
| **100** | 0 | 40.39557 | 0.599116 | 0.477676 | 2.013404 | 1.217446 | 5.7645 | Inside AD |

| | | | | | | | | |
|---|---|---|---|---|---|---|---|---|
| **106** | 0 | 37.51329 | 0.6515 | 0.340451 | 1.95176 | 1.134455 | 6.041 | Inside AD |
| **109** | 0.415315 | 45.15794 | 0.610009 | 0.568478 | 2.011134 | 1.392915 | 6.0362 | Inside AD |
| **112** | 0 | 40.26962 | 0.613234 | 0.454529 | 2.299089 | 0.971666 | 6.1938 | Inside AD |
| **115** | 9.672211 | 73.85074 | 0.570782 | 0.526709 | 2.159821 | 0.366849 | 6.2676 | Inside AD |
| **117** | 5.712963 | 68.76923 | 0.589627 | 0.448385 | 2.462004 | 1.112109 | 5.2976 | Inside AD |
| **118** | 0 | 68.70251 | 0.57702 | 0.474379 | 2.468189 | 0.803484 | 5.3224 | Inside AD |
| **121** | 0 | 52.60903 | 0.587718 | 0.32994 | 1.929043 | 1.523964 | 6.39794 | Inside AD |
| **123** | 8.271566 | 32.56201 | 0.613171 | 0.507173 | 1.889885 | 1.304049 | 6.21467 | Inside AD |
| **125** | 0 | 36.3628 | 0.596699 | 0.333583 | 2.019777 | 1.42107 | 6.055517 | Inside AD |
| **127** | 18.47471 | 38.49365 | 0.601494 | 0.492174 | 2.033653 | 0.599168 | 7.09691 | Inside AD |
| **129** | 0 | 68.0767 | 0.562324 | 0.620494 | 1.953196 | 0.970292 | 6.376751 | Inside AD |
| **32** | 0 | 47.78886 | 0.669899 | 0.315484 | 2.166342 | 0.025653 | 5.956 | Inside AD |
| **65** | 0 | 33.32108 | 0.642793 | 0.285412 | 2.219891 | 1.271223 | 5.921 | Inside AD |
| **111** | 0 | 8.412981 | 0.660172 | 0.493876 | 2.215082 | 1.357627 | 5.6216 | Inside AD |
| **113** | 36.90745 | 47.9493 | 0.592391 | 0.458333 | 2.224065 | 1.155503 | 5.1373 | Inside AD |
| **119** | 15.37618 | 81.55946 | 0.588226 | 0.367356 | 1.957351 | 1.137325 | 5.337242 | Inside AD |

Model 2 test set

| compound | ASAP4 | ENONCS | FASA- | vsurf_CW2 | vsurf_EWmin3 | vsurf_HB8 | pIC50_Tbr | AD Info. |
|---|---|---|---|---|---|---|---|---|
| **2** | 31.19218 | 45.8219 | 0.433305 | 2.074778 | -5.44163 | 0.109462 | 7.201 | Inside AD |
| **3** | 25.50916 | 43.12248 | 0.400739 | 1.911512 | -5.84208 | 0.305172 | 6.979 | Inside AD |
| **7** | 32.27361 | 12.00667 | 0.353601 | 1.869522 | -5.28037 | 0.027525 | 6.04 | Inside AD |
| **8** | 35.07656 | 52.34947 | 0.416996 | 2.219821 | -6.10811 | 0.375 | 6.496 | Inside AD |
| **10** | 0 | 44.58005 | 0.376423 | 2.013416 | -5.23149 | 0 | 5.174 | Inside AD |
| **14** | 0 | 39.78829 | 0.397198 | 1.983167 | -5.2636 | 0 | 5.93 | Inside AD |
| **16** | 0 | 42.92602 | 0.393604 | 1.983631 | -5.09041 | 0.000374 | 4.866 | Inside AD |
| **17** | 0 | 44.32691 | 0.36958 | 2.028338 | -5.1568 | 0 | 4.207 | Inside AD |
| **25** | 0 | 40.47197 | 0.33999 | 1.890907 | -5.18296 | 0 | 4.627 | Inside AD |
| **27** | 0 | 41.95494 | 0.366856 | 1.987984 | -5.18321 | 0 | 4.97 | Inside AD |
| **30** | 0 | 38.23995 | 0.386312 | 2.223136 | -5.33426 | 0 | 5.795 | Inside AD |

| | | | | | | | | |
|---|---|---|---|---|---|---|---|---|
| **32** | 0 | 47.65345 | 0.325825 | 2.166884 | -5.3217 | 0 | 5.956 | Inside AD |
| **34** | 0 | 45.76171 | 0.395704 | 1.977555 | -5.26201 | 0 | 6.411 | Inside AD |
| **37** | 0 | 33.18797 | 0.406769 | 1.93798 | -5.32239 | 0.070786 | 6.321 | Inside AD |
| **47** | 0 | 44.29522 | 0.372858 | 2.199081 | -4.88008 | 0 | 4.582 | Inside AD |
| **49** | 1.37093 | 65.93874 | 0.389234 | 2.2719 | -5.28888 | 0.000372 | 5.368 | Inside AD |
| **51** | 0 | 39.10281 | 0.407172 | 2.016006 | -5.25237 | 0.042736 | 6.164 | Inside AD |
| **54** | 0 | 41.98426 | 0.390135 | 2.018521 | -5.46583 | 0.062695 | 5.746 | Inside AD |
| **60** | 0.020816 | 30.16848 | 0.370181 | 1.802282 | -5.77637 | 0.258792 | 5.681 | Inside AD |
| **64** | 0 | 44.9625 | 0.404165 | 1.982064 | -5.28009 | 0 | 4.967 | Inside AD |
| **65** | 10.09115 | 32.10581 | 0.386808 | 2.16488 | -5.25278 | 0.00064 | 5.921 | Inside AD |
| **66** | 0.149646 | 36.49195 | 0.378849 | 1.925833 | -5.12022 | 1.14E-07 | 4.781 | Inside AD |
| **71** | 1.107578 | 24.15586 | 0.408658 | 1.875293 | -5.6271 | 0.219786 | 6.755 | Inside AD |
| **73** | 31.63815 | 52.81615 | 0.414802 | 2.065726 | -5.72129 | 0.227817 | 7.142 | Inside AD |
| **75** | 15.17969 | 27.76629 | 0.428722 | 2.009684 | -5.27659 | 0 | 6.694 | Inside AD |
| **79** | 0 | 46.36615 | 0.424645 | 2.248965 | -5.2849 | 0 | 6.178 | Inside AD |
| **81** | 7.976002 | 32.1309 | 0.37849 | 2.114218 | -5.80459 | 0.196365 | 5.704 | Inside AD |
| **85** | 8.656025 | 1.16E-07 | 0.405172 | 2.053519 | -5.59064 | 0.0811 | 5.377 | Inside AD |
| **87** | 0.241443 | 63.26138 | 0.43959 | 2.117366 | -5.82999 | 0.249781 | 5.597 | Inside AD |
| **94** | 10.65459 | 33.16004 | 0.365597 | 2.311938 | -6.58529 | 0.970033 | 5.556 | Inside AD |
| **97** | 9.061178 | 38.30212 | 0.375243 | 2.212685 | -6.7001 | 0.973165 | 5.3936 | Inside AD |
| **107** | 26.9096 | 42.53354 | 0.405047 | 2.242193 | -6.01175 | 0.494692 | 6.0362 | Inside AD |
| **109** | 17.72386 | 43.36472 | 0.41021 | 2.080272 | -6.53793 | 0.859507 | 6.0362 | Inside AD |
| **111** | 0 | 8.526318 | 0.338851 | 2.250813 | -5.28421 | 0 | 5.6216 | Inside AD |
| **112** | 5.066218 | 39.87518 | 0.416057 | 2.382447 | -5.41264 | 0.000162 | 6.1938 | Inside AD |
| **116** | 6.295662 | 40.9731 | 0.384084 | 2.341011 | -6.42711 | 1.065806 | 4.8116 | Inside AD |
| **119** | 12.95804 | 80.07457 | 0.412457 | 1.927289 | -5.57595 | 0.24032 | 5.337242 | Inside AD |
| **123** | 12.73744 | 38.78348 | 0.387244 | 1.938247 | -5.47556 | 0.024499 | 6.21467 | Inside AD |
| **126** | 3.936389 | 24.25307 | 0.394227 | 1.969981 | -5.20037 | 0 | 5.920819 | Inside AD |
| **127** | 20.03294 | 37.41629 | 0.390794 | 1.991145 | -6.04709 | 0.345919 | 7.09691 | Inside AD |

Model 3 test set

| compound | F1 | F2 | F3 | F5 | F7 | F8 | F9 | S | pIC50_Tbr | AD Info. |
|---|---|---|---|---|---|---|---|---|---|---|
| **2** | 1 | 1 | 0 | 1 | 0 | 0 | 1 | -134.702 | 7.201 | Inside AD |
| **3** | 1 | 1 | 0 | 1 | 0 | 0 | 1 | -136.419 | 6.979 | Inside AD |
| **5** | 0 | 1 | 0 | 0 | 0 | 0 | 1 | -122.827 | 6.164 | Inside AD |
| **6** | 0 | 1 | 0 | 1 | 0 | 0 | 1 | -134.739 | 5.849 | Inside AD |
| **17** | 1 | 1 | 0 | 0 | 0 | 0 | 0 | -124.361 | 4.207 | Inside AD |
| **21** | 1 | 1 | 0 | 1 | 0 | 0 | 1 | -112.549 | 6.195 | Inside AD |
| **22** | 1 | 1 | 0 | 1 | 0 | 0 | 1 | -115.82 | 5.89 | Inside AD |
| **28** | 0 | 1 | 0 | 0 | 0 | 0 | 0 | -120.911 | 3.467 | Inside AD |
| **29** | 1 | 1 | 0 | 0 | 0 | 0 | 0 | -124.299 | 5.581 | Inside AD |
| **31** | 1 | 1 | 0 | 0 | 0 | 0 | 0 | -122.104 | 4.749 | Inside AD |
| **32** | 1 | 1 | 0 | 0 | 0 | 0 | 0 | -122.521 | 5.956 | Inside AD |
| **34** | 1 | 1 | 0 | 0 | 0 | 0 | 0 | -124.588 | 6.411 | Inside AD |
| **42** | 1 | 1 | 0 | 0 | 0 | 0 | 0 | -126.037 | 5.703 | Inside AD |
| **44** | 0 | 1 | 0 | 1 | 0 | 0 | 0 | -136.973 | 4.507 | Inside AD |
| **46** | 1 | 1 | 1 | 0 | 0 | 0 | 0 | -134.378 | 6.523 | Inside AD |
| **52** | 1 | 1 | 0 | 1 | 0 | 0 | 0 | -134.43 | 4.989 | Inside AD |
| **57** | 1 | 1 | 0 | 1 | 0 | 0 | 0 | -134.815 | 6.648 | Inside AD |
| **59** | 1 | 1 | 0 | 0 | 0 | 0 | 0 | -124.516 | 5.609 | Inside AD |
| **62** | 1 | 1 | 0 | 0 | 0 | 0 | 0 | -125.774 | 4.921 | Inside AD |
| **63** | 1 | 1 | 0 | 0 | 0 | 0 | 0 | -124.132 | 4.66 | Inside AD |
| **72** | 1 | 1 | 0 | 1 | 0 | 0 | 0 | -134.32 | 6.788 | Inside AD |
| **73** | 1 | 1 | 0 | 1 | 0 | 1 | 0 | -135.04 | 7.142 | Inside AD |
| **78** | 1 | 1 | 0 | 0 | 0 | 1 | 0 | -136.633 | 6.588 | Inside AD |
| **83** | 1 | 1 | 0 | 1 | 0 | 0 | 0 | -130.706 | 6.084 | Inside AD |
| **84** | 1 | 1 | 0 | 1 | 0 | 0 | 0 | -130.741 | 5.496 | Inside AD |
| **85** | 1 | 1 | 0 | 0 | 0 | 0 | 0 | -136.992 | 5.377 | Inside AD |
| **86** | 1 | 1 | 1 | 0 | 0 | 0 | 0 | -135.603 | 5.767 | Inside AD |
| **90** | 1 | 1 | 1 | 0 | 1 | 0 | 0 | -135.195 | 6.7959 | Inside AD |
| **91** | 0 | 0 | 1 | 0 | 0 | 0 | 1 | -119.118 | 6.294 | Outside AD |

| | | | | | | | | | | |
|---|---|---|---|---|---|---|---|---|---|---|
| **98** | 1 | 1 | 0 | 1 | 0 | 0 | 0 | -143.953 | 5.9431 | Inside AD |
| **99** | 1 | 1 | 0 | 1 | 0 | 0 | 0 | -142.138 | 6.0862 | Inside AD |
| **107** | 1 | 1 | 0 | 1 | 0 | 0 | 0 | -141.244 | 6.0362 | Inside AD |
| **109** | 1 | 1 | 0 | 1 | 0 | 0 | 0 | -130.599 | 6.0362 | Inside AD |
| **111** | 0 | 1 | 0 | 0 | 1 | 0 | 0 | -126.781 | 5.6216 | Inside AD |
| **113** | 1 | 1 | 0 | 1 | 0 | 0 | 0 | -138.039 | 5.1373 | Inside AD |
| **115** | 1 | 1 | 1 | 0 | 0 | 0 | 0 | -126.629 | 6.2676 | Inside AD |
| **116** | 0 | 1 | 1 | 0 | 0 | 0 | 0 | -131.322 | 4.8116 | Inside AD |
| **117** | 1 | 1 | 1 | 0 | 0 | 0 | 0 | -130.158 | 5.2976 | Inside AD |
| **121** | 1 | 1 | 0 | 1 | 0 | 0 | 0 | -131.559 | 6.39794 | Inside AD |
| **123** | 1 | 1 | 0 | 1 | 0 | 0 | 0 | -133.926 | 6.21467 | Inside AD |
| **124** | 1 | 1 | 0 | 1 | 0 | 0 | 0 | -134.767 | 6.443698 | Inside AD |
| **125** | 1 | 1 | 0 | 1 | 0 | 0 | 0 | -133.748 | 6.055517 | Inside AD |
| **129** | 1 | 1 | 0 | 1 | 1 | 0 | 0 | -133.341 | 6.376751 | Inside AD |
| **127** | 1 | 1 | 0 | 1 | 0 | 0 | 0 | -124.131 | 7.09691 | Inside AD |

Model 4 test set

| compound | F1 | F2 | F3-5 | F7-8 | F9 | S | pIC50_Tbr | AD Info. |
|---|---|---|---|---|---|---|---|---|
| **2** | 1 | 1 | 1 | 0 | 1 | -134.702 | 7.201 | Inside AD |
| **3** | 1 | 1 | 1 | 0 | 1 | -136.419 | 6.979 | Inside AD |
| **5** | 0 | 1 | 0 | 0 | 1 | -122.827 | 6.164 | Inside AD |
| **6** | 0 | 1 | 1 | 0 | 1 | -134.739 | 5.849 | Inside AD |
| **17** | 1 | 1 | 0 | 0 | 0 | -124.361 | 4.207 | Inside AD |
| **21** | 1 | 1 | 1 | 0 | 1 | -112.549 | 6.195 | Inside AD |
| **22** | 1 | 1 | 1 | 0 | 1 | -115.82 | 5.89 | Inside AD |
| **28** | 0 | 1 | 0 | 0 | 0 | -120.911 | 3.467 | Inside AD |
| **29** | 1 | 1 | 0 | 0 | 0 | -124.299 | 5.581 | Inside AD |
| **31** | 1 | 1 | 0 | 0 | 0 | -122.104 | 4.749 | Inside AD |
| **32** | 1 | 1 | 0 | 0 | 0 | -122.521 | 5.956 | Inside AD |
| **34** | 1 | 1 | 0 | 0 | 0 | -124.588 | 6.411 | Inside AD |

| | | | | | | | | |
|---|---|---|---|---|---|---|---|---|
| **42** | 1 | 1 | 0 | 0 | 0 | -126.037 | 5.703 | Inside AD |
| **44** | 0 | 1 | 1 | 0 | 0 | -136.973 | 4.507 | Inside AD |
| **46** | 1 | 1 | 1 | 0 | 0 | -134.378 | 6.523 | Inside AD |
| **52** | 1 | 1 | 1 | 0 | 0 | -134.43 | 4.989 | Inside AD |
| **57** | 1 | 1 | 1 | 0 | 0 | -134.815 | 6.648 | Inside AD |
| **59** | 1 | 1 | 0 | 0 | 0 | -124.516 | 5.609 | Inside AD |
| **62** | 1 | 1 | 0 | 0 | 0 | -125.774 | 4.921 | Inside AD |
| **63** | 1 | 1 | 0 | 0 | 0 | -124.132 | 4.66 | Inside AD |
| **72** | 1 | 1 | 1 | 0 | 0 | -134.32 | 6.788 | Inside AD |
| **73** | 1 | 1 | 1 | 1 | 0 | -135.04 | 7.142 | Inside AD |
| **78** | 1 | 1 | 0 | 1 | 0 | -136.633 | 6.588 | Inside AD |
| **83** | 1 | 1 | 1 | 0 | 0 | -130.706 | 6.084 | Inside AD |
| **84** | 1 | 1 | 1 | 0 | 0 | -130.741 | 5.496 | Inside AD |
| **85** | 1 | 1 | 0 | 0 | 0 | -136.992 | 5.377 | Inside AD |
| **86** | 1 | 1 | 1 | 0 | 0 | -135.603 | 5.767 | Inside AD |
| **90** | 1 | 1 | 1 | 1 | 0 | -135.195 | 6.7959 | Inside AD |
| **91** | 0 | 0 | 1 | 0 | 1 | -119.118 | 6.294 | Outside AD |
| **98** | 1 | 1 | 1 | 0 | 0 | -143.953 | 5.9431 | Inside AD |
| **99** | 1 | 1 | 1 | 0 | 0 | -142.138 | 6.0862 | Inside AD |
| **107** | 1 | 1 | 1 | 0 | 0 | -141.244 | 6.0362 | Inside AD |
| **109** | 1 | 1 | 1 | 0 | 0 | -130.599 | 6.0362 | Inside AD |
| **111** | 0 | 1 | 0 | 1 | 0 | -126.781 | 5.6216 | Inside AD |
| **113** | 1 | 1 | 1 | 0 | 0 | -138.039 | 5.1373 | Inside AD |
| **115** | 1 | 1 | 1 | 0 | 0 | -126.629 | 6.2676 | Inside AD |
| **116** | 0 | 1 | 1 | 0 | 0 | -131.322 | 4.8116 | Inside AD |
| **117** | 1 | 1 | 1 | 0 | 0 | -130.158 | 5.2976 | Inside AD |
| **121** | 1 | 1 | 1 | 0 | 0 | -131.559 | 6.39794 | Inside AD |
| **123** | 1 | 1 | 1 | 0 | 0 | -133.926 | 6.21467 | Inside AD |
| **124** | 1 | 1 | 1 | 0 | 0 | -134.767 | 6.443698 | Inside AD |
| **125** | 1 | 1 | 1 | 0 | 0 | -133.748 | 6.055517 | Inside AD |
| **129** | 1 | 1 | 1 | 1 | 0 | -133.341 | 6.376751 | Inside AD |

| | | | | | | | | |
|---|---|---|---|---|---|---|---|---|
| **127** | 1 | 1 | 1 | 0 | 0 | -124.131 | 7.09691 | Inside AD |

Model 5 test set

| compound | F1 | F3-5 | F7-8 | F9 | FASA+ | FASA_H | pIC50_Tbr | AD Info. |
|---|---|---|---|---|---|---|---|---|
| **2** | 1 | 1 | 0 | 1 | 0.572666 | 0.624685 | 7.201 | Inside AD |
| **3** | 1 | 1 | 0 | 1 | 0.603196 | 0.666645 | 6.979 | Inside AD |
| **5** | 0 | 0 | 0 | 1 | 0.617093 | 0.58236 | 6.164 | Inside AD |
| **6** | 0 | 1 | 0 | 1 | 0.600926 | 0.595556 | 5.849 | Inside AD |
| **17** | 1 | 0 | 0 | 0 | 0.62551 | 0.685184 | 4.207 | Inside AD |
| **21** | 1 | 1 | 0 | 1 | 0.588926 | 0.603349 | 6.195 | Inside AD |
| **22** | 1 | 1 | 0 | 1 | 0.594313 | 0.594821 | 5.89 | Inside AD |
| **28** | 0 | 0 | 0 | 0 | 0.687585 | 0.702185 | 3.467 | Inside AD |
| **29** | 1 | 0 | 0 | 0 | 0.681422 | 0.830634 | 5.581 | Inside AD |
| **31** | 1 | 0 | 0 | 0 | 0.670478 | 0.767222 | 4.749 | Inside AD |
| **32** | 1 | 0 | 0 | 0 | 0.669899 | 0.763364 | 5.956 | Inside AD |
| **34** | 1 | 0 | 0 | 0 | 0.603052 | 0.768768 | 6.411 | Inside AD |
| **42** | 1 | 0 | 0 | 0 | 0.627634 | 0.743803 | 5.703 | Inside AD |
| **44** | 0 | 1 | 0 | 0 | 0.631539 | 0.75148 | 4.507 | Inside AD |
| **46** | 1 | 1 | 0 | 0 | 0.589434 | 0.700023 | 6.523 | Inside AD |
| **52** | 1 | 1 | 0 | 0 | 0.588006 | 0.657847 | 4.989 | Inside AD |
| **57** | 1 | 1 | 0 | 0 | 0.578762 | 0.694482 | 6.648 | Inside AD |
| **59** | 1 | 0 | 0 | 0 | 0.613145 | 0.756504 | 5.609 | Inside AD |
| **62** | 1 | 0 | 0 | 0 | 0.682925 | 0.844676 | 4.921 | Inside AD |
| **63** | 1 | 0 | 0 | 0 | 0.677116 | 0.843556 | 4.66 | Inside AD |
| **72** | 1 | 1 | 0 | 0 | 0.582402 | 0.669497 | 6.788 | Inside AD |
| **73** | 1 | 1 | 1 | 0 | 0.592159 | 0.636215 | 7.142 | Inside AD |
| **78** | 1 | 0 | 1 | 0 | 0.572957 | 0.68332 | 6.588 | Inside AD |
| **83** | 1 | 1 | 0 | 0 | 0.631182 | 0.723927 | 6.084 | Inside AD |
| **84** | 1 | 1 | 0 | 0 | 0.653324 | 0.707169 | 5.496 | Inside AD |
| **85** | 1 | 0 | 0 | 0 | 0.598871 | 0.570153 | 5.377 | Inside AD |

| | | | | | | | | |
|---|---|---|---|---|---|---|---|---|
| **86** | 1 | 1 | 0 | 0 | 0.594943 | 0.5785 | 5.767 | Inside AD |
| **90** | 1 | 1 | 1 | 0 | 0.528332 | 0.636959 | 6.7959 | Inside AD |
| **91** | 0 | 1 | 0 | 1 | 0.533971 | 0.609399 | 6.294 | Inside AD |
| **98** | 1 | 1 | 0 | 0 | 0.639398 | 0.738523 | 5.9431 | Inside AD |
| **99** | 1 | 1 | 0 | 0 | 0.628924 | 0.738573 | 6.0862 | Inside AD |
| **107** | 1 | 1 | 0 | 0 | 0.597786 | 0.675473 | 6.0362 | Inside AD |
| **109** | 1 | 1 | 0 | 0 | 0.610009 | 0.719412 | 6.0362 | Inside AD |
| **115** | 1 | 1 | 0 | 0 | 0.570782 | 0.754577 | 6.2676 | Inside AD |
| **116** | 0 | 1 | 0 | 0 | 0.627984 | 0.656871 | 4.8116 | Inside AD |
| **117** | 1 | 1 | 0 | 0 | 0.589627 | 0.676432 | 5.2976 | Inside AD |
| **121** | 1 | 1 | 0 | 0 | 0.587718 | 0.686159 | 6.39794 | Inside AD |
| **123** | 1 | 1 | 0 | 0 | 0.613171 | 0.748509 | 6.21467 | Inside AD |
| **124** | 1 | 1 | 0 | 0 | 0.629814 | 0.745626 | 6.443698 | Inside AD |
| **125** | 1 | 1 | 0 | 0 | 0.596699 | 0.650484 | 6.055517 | Inside AD |
| **127** | 1 | 1 | 0 | 0 | 0.601494 | 0.692768 | 7.09691 | Inside AD |
| **129** | 1 | 1 | 1 | 0 | 0.562324 | 0.665742 | 6.376751 | Inside AD |
| **111** | 0 | 0 | 1 | 0 | 0.660172 | 0.628941 | 5.6216 | Inside AD |
| **113** | 1 | 1 | 0 | 0 | 0.592391 | 0.694526 | 5.1373 | Inside AD |
